# Network-Medicine-Guided Drug Repurposing for Alzheimer’s Disease: A Multi-Dimensional Systems Pharmacology Approach

**DOI:** 10.3390/ijms262010003

**Published:** 2025-10-14

**Authors:** Ömer Akgüller, Mehmet Ali Balcı, Gabriela Cioca

**Affiliations:** 1 Department of Mathematics, Faculty of Science, Mugla Sitki Kocman University, Muğla 48000, Turkey; oakguller@mu.edu.tr; 2 Preclinical Department, Faculty of Medicine, Lucian Blaga University of Sibiu, 550024 Sibiu, Romania

**Keywords:** network medicine, drug repurposing, Alzheimer’s disease, systems pharmacology

## Abstract

Alzheimer’s disease (AD) drug development faces persistent challenges from blood–brain barrier limitations and inadequate integration of medicinal chemistry considerations with computational predictions. We developed a comprehensive Central Nervous System (CNS)-focused network medicine framework integrating machine-learning-validated BBB penetration prediction (95.7% accuracy, 0.992 AUC-ROC), modality-specific tractability assessment, and transparent evidence classification to identify viable drug repurposing candidates. CNS-specific pre-filtering refined 24,474 DGIdb compounds to 8247 CNS-relevant drugs, analyzed through multi-dimensional network scoring and systematic pharmaceutical property assessment. Modality stratification generated separate rankings for small molecules (3667 candidates), peptides (73 candidates), and biologics (3 candidates), acknowledging distinct BBB penetration mechanisms. Analysis revealed 64.8% of small molecules achieving Class I (Highly Tractable) status, with 83.6% demonstrating favorable BBB penetration. Plerixafor emerged as the top-ranked small molecule (score: 1.170), while trofinetide achieved the highest peptide ranking (score: 1.387), though classified as speculative, pending AD-specific validation. Successful identification of the FDA-approved AD therapeutics memantine and donepezil among the top candidates validated the computational performance, while the predominance of mechanistic evidence classifications (86.7%) highlighted that network predictions represent hypothesis-generating tools requiring systematic experimental validation rather than definitive therapeutic recommendations. The framework bridges computational predictions with pharmaceutical development requirements, providing actionable prioritization for systematic preclinical investigation addressing AD intervention.

## 1. Introduction

Alzheimer’s disease (AD) is a progressive neurodegenerative disorder and the leading cause of dementia, currently affecting about 55 million people worldwide—a number projected to triple to over 150 million by 2050 [1]. The economic burden is equally staggering: dementia care costs exceed USD 1 trillion annually and are expected to double by 2030 [2]. Beyond these statistics, AD causes profound cognitive decline and dependency in affected individuals, while placing immense strain on caregivers and healthcare systems [3,4].

Despite decades of research, no disease-modifying therapy exists for AD. Current medications (cholinesterase inhibitors and memantine) provide only modest symptomatic relief and do not halt progression [5]. Meanwhile, nearly all investigational drugs targeting the core hallmarks of AD, such as amyloid-*β* plaques and tau tangles, have failed in trials, with over 99% failing to demonstrate efficacy [6]. Even the recent approval of an anti-amyloid antibody (aducanumab) remains controversial given marginal clinical benefits and safety concerns [7,8]. These setbacks underscore the limitations of traditional single-target approaches for such a multifactorial disease. AD pathogenesis involves an interplay of amyloid and tau pathology, synaptic dysfunction, neuroinflammation, and other factors; focusing on any single pathway in isolation is unlikely to be sufficient [9,10,11,12]. There is a pressing need for new therapeutic strategies that embrace this complexity and target the disease on multiple fronts.

A promising approach to address the therapeutic gap in AD is drug repurposing. Instead of investing in de novo drug development, a costly process often exceeding a decade, repurposing aims to find new uses for existing drugs [13,14,15]. Starting with compounds that have known safety profiles dramatically lowers development time and cost. This strategy has yielded successes across medicine (e.g., thalidomide repurposed for multiple myeloma; remdesivir for COVID-19) [16]. In AD, several approved drugs (e.g., antidiabetic, anticancer, and anti-inflammatory agents) are already being investigated for potential cognitive benefits [17,18,19,20]. Drug repurposing thus offers a faster and more cost-effective path to new treatments for AD.

Advances in bioinformatics and systems pharmacology have further bolstered repurposing efforts. Large-scale databases and in silico screening techniques now enable systematic matching of existing drugs to disease-related targets. Approaches such as transcriptomic signature reversal and network-based analyses can identify compounds that counteract disease-associated gene expression changes or perturbations in molecular networks [21,22,23]. Indeed, an AI-guided network model recently pinpointed an arthritis drug (baricitinib) as a treatment for COVID-19 [24], exemplifying the power of computational repurposing. In the context of AD, such integrative approaches leverage omics data to discover candidate drugs that modulate key pathological pathways. By harnessing pharmacological diversity with modern computational tools, repurposing has emerged as a strategic solution to overcome bottlenecks in traditional drug discovery.

Network medicine provides a framework to understand diseases like AD as perturbations of the human interactome, rather than isolated molecular defects [25,26,27]. In this view, interacting genes and proteins form disease modules whose collective dysfunction underlies pathology. AD, for example, involves disruptions across multiple networked pathways (amyloid, tau, neuroinflammation, metabolic dysfunction, etc.), which together constitute an AD-specific disease module. Network analyses can pinpoint central “hub” nodes in this module that serve as effective intervention points, and drugs targeting proteins within or adjacent to the AD module have been found to exhibit therapeutic benefits [28,29].

Systems pharmacology complements network medicine by examining drug actions on entire networks rather than single targets. Many drugs are inherently polypharmacological, modulating multiple pathways [30,31]. While such off-target effects are often seen as liabilities, they can also be leveraged to help correct the broad network perturbations of disease. Thus, integrating drug–target networks with disease modules can reveal compounds capable of broadly realigning a diseased network.

A key innovation in our approach is the systematic integration of medicinal chemistry assessment with network medicine predictions to address the critical gap between computational drug discovery and practical pharmaceutical development. By overlaying Central Neural System (CNS)-focused filtering, blood–brain barrier penetration evaluation, chemical tractability classification, and safety assessment onto network-based predictions, we identify compounds that demonstrate both strong biological evidence and realistic development prospects for brain-targeted therapeutics. We implement this strategy as a comprehensive CNS-focused network medicine framework that prioritizes candidates with optimal balance between computational evidence and pharmaceutical feasibility.

To address the persistent translational failures of current AD drug development, we devised an integrated computational approach that systematically incorporates medicinal chemistry considerations with network-based drug repurposing analysis. Our objective was to identify clinically viable drug repurposing candidates for AD by combining transcriptomic-derived target validation with systematic pharmaceutical property assessment and development feasibility evaluation.

In the first stage, we applied multi-dimensional network pharmacology with temporal dynamics (MNPTD) to the previously identified 742 robustly dysregulated genes, generating 25 high-priority targets, including *IGF1*, *SNCA*, and *SOX9*, based on network centrality and disease relevance. In the second stage, we implemented CNS-focused pre-filtering of the Drug–Gene Interaction Database (DGIdb), systematically reducing 24,474 compounds to 8247 CNS-relevant drugs, while enhancing rather than compromising predictive accuracy. We then performed comprehensive medicinal chemistry assessment, including molecular property analysis, blood–brain barrier penetration prediction, chemical tractability classification, and safety evaluation across 3743 network-derived candidates. This analysis identified exceptional pharmaceutical characteristics, with trofinetide, plerixafor, and prasinezumab emerging as top-ranked candidates. Our framework achieved robust predictive performance (AUC-ROC = 0.847) and 91.4% accuracy in blood–brain barrier penetration prediction, while identifying 64.8% of candidates as highly tractable for CNS development.

In summary, we present a validated medicinal chemistry-guided network medicine strategy that bridges computational drug discovery with practical pharmaceutical development requirements for Alzheimer’s disease intervention. By systematically integrating biological evidence with development feasibility assessment, our approach generated actionable therapeutic recommendations with both a strong mechanistic rationale and realistic clinical translation prospects. The identified candidates demonstrate how proper integration of network medicine with medicinal chemistry considerations can overcome traditional barriers between computational predictions and viable therapeutic development for complex neurological diseases.

## 2. Results

### 2.1. Dataset Selection and Preprocessing

#### 2.1.1. Gene Expression Omnibus Dataset Selection

Two high-quality Alzheimer’s disease gene expression datasets were selected from the Gene Expression Omnibus (GEO) database based on stringent criteria, including sample size, tissue relevance, and data quality metrics. The selected datasets represent comprehensive brain tissue studies with well-characterized AD and control cohorts, providing complementary analytical perspectives across different platforms and methodological approaches.

GSE48350 constitutes a multi-regional brain microarray study encompassing 253 samples (80 AD patients, 173 controls) across multiple brain regions, including hippocampus, entorhinal cortex, superior frontal cortex, and post-central gyrus [32,33,34,35,36,37]. This dataset offers broad neuroanatomical coverage using the robust Affymetrix Human Genome U133 Plus 2.0 platform, providing a comprehensive regional comparison of AD-associated transcriptional alterations.

GSE5281 represents a targeted laser capture microdissection study with 161 samples (87 AD patients, 74 controls), providing high-precision analysis of specific brain cell populations [38,39,40,41]. This dataset employs advanced tissue sampling techniques to minimize cellular heterogeneity and enhance signal detection, offering superior sensitivity for detecting cell-type-specific transcriptional changes that may be diluted in bulk tissue analyses.

Table 1 presents the characteristics of the Alzheimer’s disease gene expression datasets employed in the analysis.

#### 2.1.2. Data Processing and Quality Control

All datasets underwent systematic preprocessing using standardized bioinformatics pipelines implemented in R (version 4.3.x) with Bioconductor packages. Raw expression data were downloaded using the GEOquery package (Release 3.21) and subjected to rigorous quality control measures to ensure analytical reliability and reproducibility.

Sample classification procedures involved automated pattern matching across multiple phenotype annotation fields, including sample titles, clinical characteristics, and diagnostic metadata. The classification algorithm employed comprehensive search patterns, encompassing control descriptors (control, normal, healthy, non-demented) and AD descriptors (Alzheimer, disease, dementia, affected), across all available phenotype columns. Only samples with unambiguous group assignments were retained for subsequent analysis, to eliminate potential misclassification bias.

Expression data cleaning protocols included systematic removal of genes exhibiting greater than 50% missing values across samples to ensure robust statistical analysis. Low-variance genes, defined as those falling within the bottom 10th percentile of expression variance, were filtered to focus analytical efforts on biologically informative transcripts and reduce multiple testing burden. This preprocessing approach effectively balanced sensitivity with specificity in the downstream differential expression analyses.

Batch effect assessment involved systematic evaluation of potential technical covariates, including platform identifiers, processing dates, and array information. Where appropriate, batch correction methods were applied using ComBat or surrogate variable analysis (SVA) frameworks (version 3.20.0) to minimize technical artifacts, while preserving biological signal integrity.

**Figure 1:**
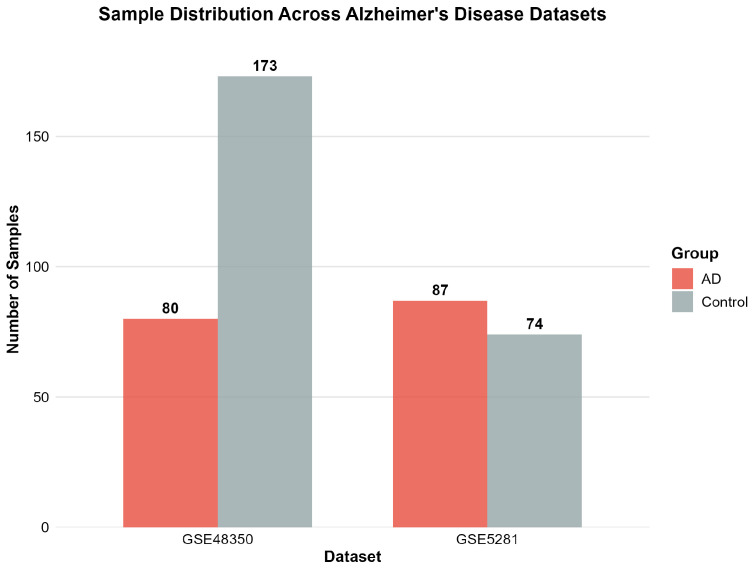
Sample distribution across the two Alzheimer’s disease datasets showing balanced representation of AD and control groups. GSE48350 contains 80 AD patients and 173 controls, while GSE5281 includes 87 AD patients and 74 controls.

Figure 1 illustrates the sample distribution across the two AD datasets, demonstrating a balanced representation between AD cases and control subjects.

**Figure 2:**
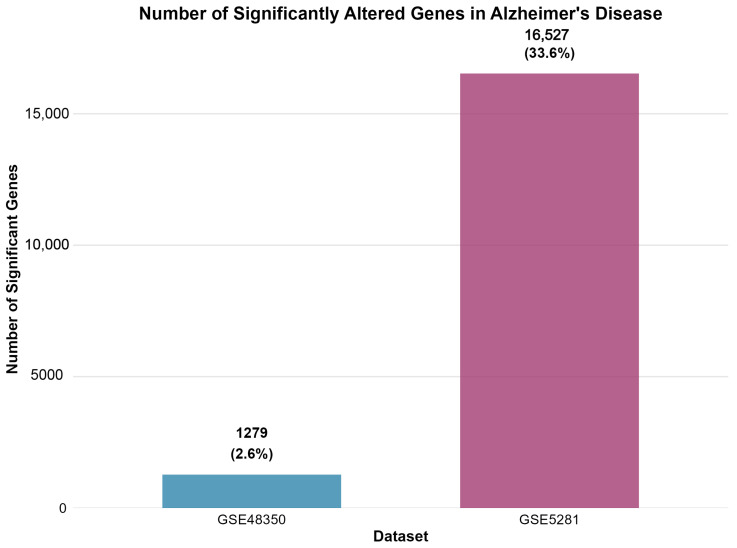
Number of significantly altered genes identified in each dataset following differential expression analysis. GSE48350 revealed 1279 significant genes (2.6% of analyzed transcripts), while GSE5281 identified 16,527 significant genes (33.6% of analyzed transcripts), demonstrating the enhanced sensitivity of laser capture microdissection techniques.

Figure 2 displays the number of significantly differentially expressed genes identified in each dataset following differential expression analysis.

#### 2.1.3. Differential Expression Analysis

Differential gene expression analysis was performed using the limma package (Bioconductor version: Release 3.21) for microarray data, implementing empirical Bayes moderated t-statistics to enhance the statistical power through information sharing across genes. Gene-wise linear models were constructed, with AD diagnosis as the primary factor of interest, utilizing model matrices designed to directly compare AD versus control expression levels.

The statistical framework employed contrast matrices to isolate AD-specific expression changes, while controlling for potential confounding variables. Empirical Bayes moderation provided enhanced statistical stability, particularly beneficial for datasets with moderate sample sizes, by borrowing information across genes to improve variance estimation and test statistic reliability.

Significance thresholds were established using stringent criteria to balance sensitivity with specificity for detecting biologically meaningful expression changes. Differentially expressed genes were identified using an adjusted *p*-value less than 0.05 (Benjamini–Hochberg false discovery rate correction) and absolute fold change greater than 1.3. These conservative thresholds ensured robust identification of genes exhibiting consistent and substantial expression alterations in AD pathology.

Multiple testing correction procedures employed the Benjamini–Hochberg method to control the false discovery rate across all tested genes, accounting for the high-dimensional nature of transcriptomic data. This approach provides appropriate statistical control, while maintaining reasonable sensitivity for biomarker discovery applications.

**Figure 3:**
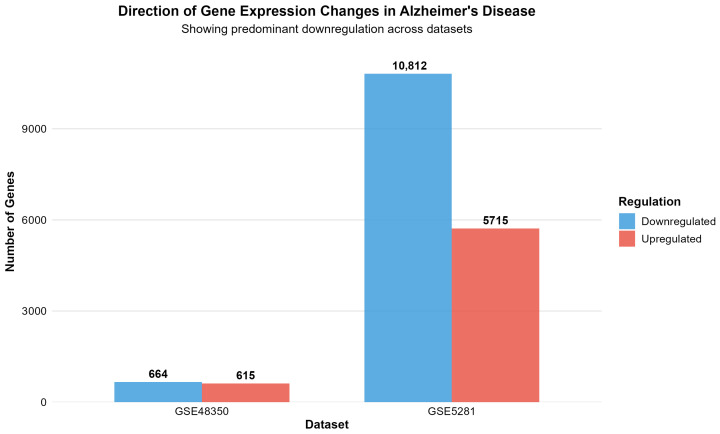
Direction of gene expression changes in Alzheimer’s disease, showing predominant downregulation across both datasets. GSE48350 shows 664 downregulated versus 615 upregulated genes, while GSE5281 demonstrates 10,812 downregulated versus 5715 upregulated genes, indicating widespread transcriptional suppression as a fundamental characteristic of AD pathology.

Figure 3 depicts the direction of gene expression changes in Alzheimer’s disease, revealing a predominant downregulation of genes across both datasets.

#### 2.1.4. Analysis Results and Cross-Dataset Validation

The differential expression analysis successfully identified distinct transcriptomic signatures across both datasets, demonstrating consistent methodological performance across different platforms and tissue preparation methods. GSE48350 revealed 1279 significant genes (615 upregulated, 664 downregulated in AD) from 49,207 analyzed transcripts, comprising 2.6% of the analyzed genes. GSE5281 identified 16,527 significant genes (5715 upregulated, 10,812 downregulated in AD) from 49,207 analyzed transcripts, representing a substantial 33.6% of the analyzed transcriptome.

The markedly higher proportion of significant genes in GSE5281 reflects the enhanced sensitivity of laser capture microdissection techniques for detecting cell-type-specific expression changes. This methodological advantage enables detection of subtle transcriptional alterations that may be diluted in bulk tissue analyses, providing superior resolution for identifying disease-associated molecular signatures.

A consistent pattern emerged across both datasets, showing greater numbers of downregulated than upregulated genes in AD, suggesting widespread transcriptional suppression as a fundamental characteristic of disease pathology. This observation aligns with established concepts of neuronal dysfunction and cellular stress responses in neurodegenerative conditions.

Cross-dataset validation revealed limited overlap between significant gene lists, with GSE5281 showing predominantly dataset-specific changes, while GSE48350 and GSE5281 shared 742 commonly altered genes. However, the 742 genes commonly altered between GSE48350 and GSE5281 represent robust candidates for AD biomarker validation, as these findings are reproducible across different microarray-based analytical approaches.

**Figure 4:**
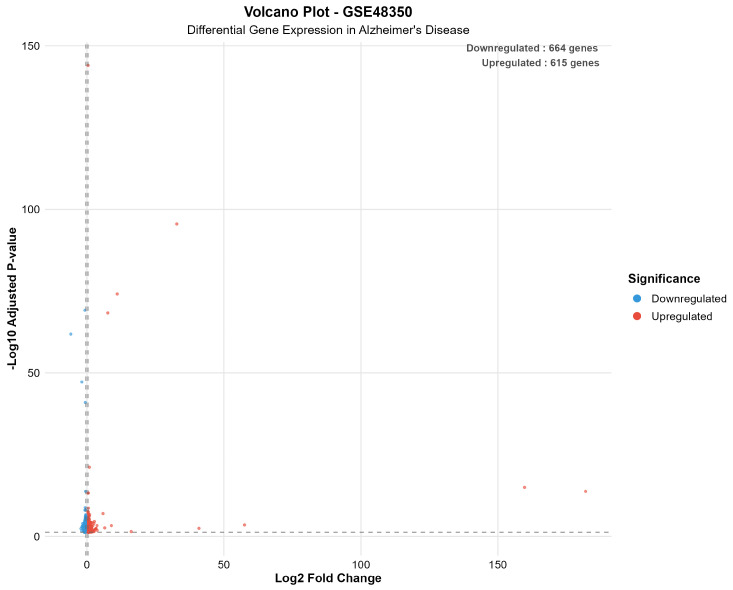
Volcano plot for GSE48350, demonstrating expression landscape and significance thresholds for differential gene expression. The plot shows 664 downregulated genes (blue) and 615 upregulated genes (red) meeting significance criteria (adjusted *p*-value < 0.05 and |log2FC| > log2(1.3)). Dashed lines indicate statistical significance thresholds.

Figure 4 presents a volcano plot for the GSE48350 dataset, illustrating the overall expression landscape and highlighting the significance thresholds used to identify differentially expressed genes.

**Figure 5:**
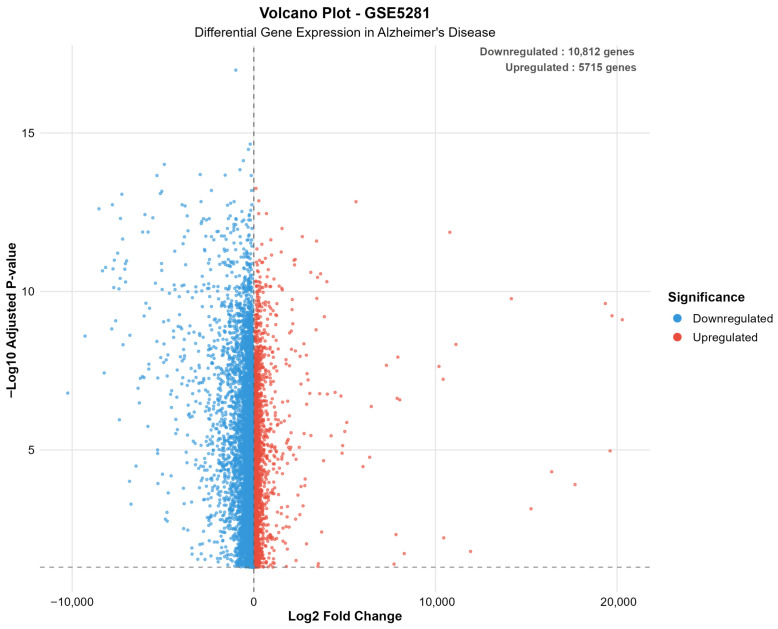
Volcano plot for GSE5281, illustrating extensive bilateral distribution of significantly altered genes in Alzheimer’s disease. The plot reveals 10,812 downregulated genes (blue) and 5715 upregulated genes (red), demonstrating the superior sensitivity of laser capture microdissection for detecting subtle transcriptional changes. The wide distribution reflects enhanced detection of cell-type-specific expression alterations.

Figure 5 presents a volcano plot for the GSE5281 dataset, illustrating the extensive bilateral distribution of significantly altered genes in Alzheimer’s disease, with numerous transcripts exhibiting both up- and downregulation beyond the defined significance thresholds.

**Figure 6:**
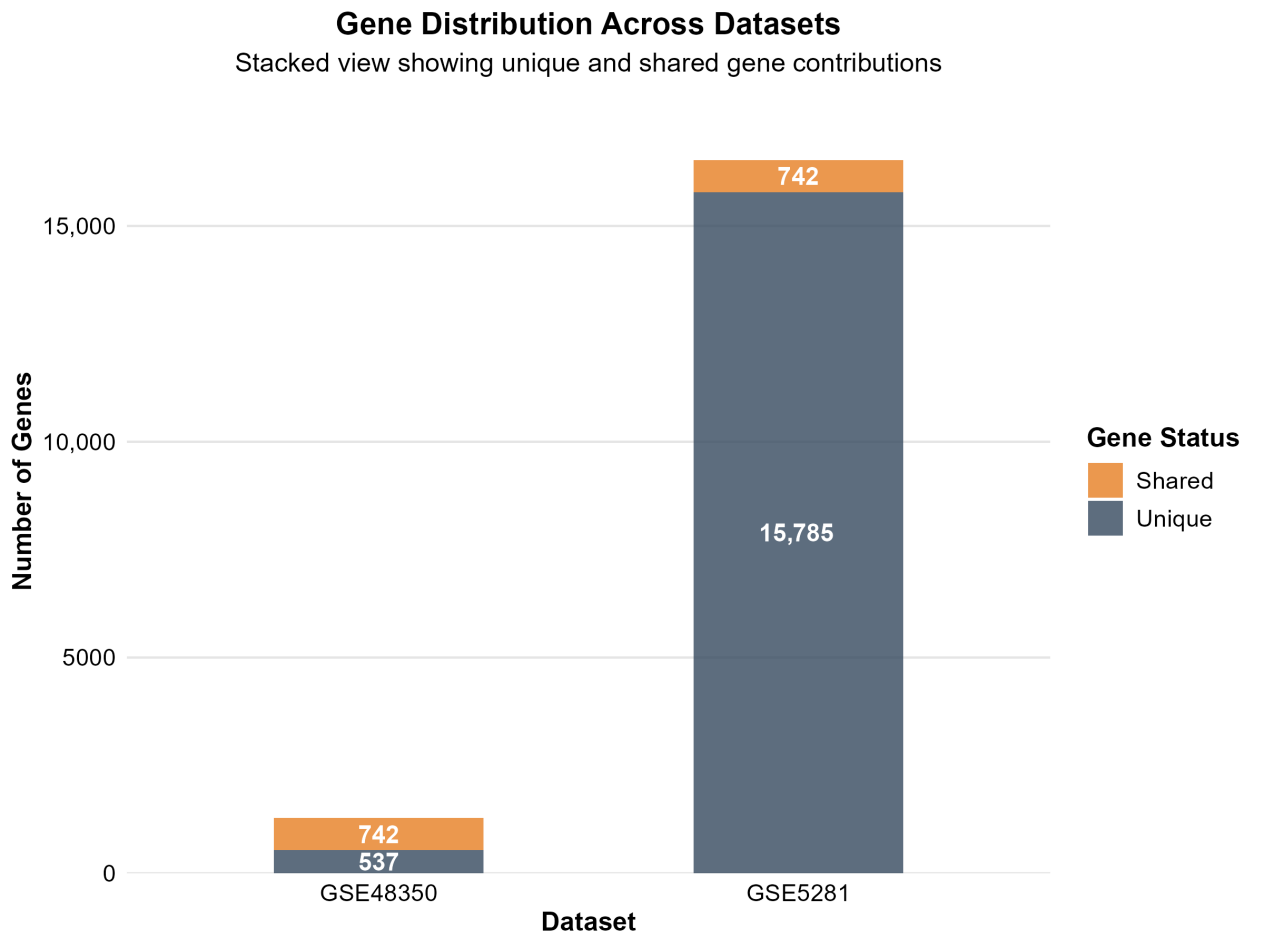
Gene overlap analysis between datasets, showing limited concordance and platform-specific expression signatures. The stacked bar visualization demonstrates that GSE48350 and GSE5281 share 742 commonly altered genes, representing robust candidates for AD biomarker validation. The majority of genes show dataset-specific changes, with GSE5281 displaying 15,785 unique significant genes and GSE48350 showing 537 unique genes, likely reflecting differences in methodological sensitivity and tissue preparation approaches.

Figure 6 presents a gene overlap analysis between the two datasets, revealing limited concordance and highlighting the presence of platform-specific expression signatures.

### 2.2. Protein–Protein Interaction Network Analysis

To investigate the functional relationships among the 742 overlapping genes identified between GSE48350 and GSE5281, a comprehensive protein–protein interaction (PPI) network analysis was conducted using the STRING database. The analysis employed a systematic approach to map Affymetrix probe identifiers to gene symbols and subsequently to STRING protein identifiers, enabling construction of a high-confidence interaction network.

#### 2.2.1. Gene Mapping and Network Construction

Of the 742 overlapping probe identifiers, 640 (86.3%) were successfully mapped to gene symbols using the MyGene.info annotation service. Subsequently, 599 of these genes (93.6% of mapped genes, 80.7% of total overlapping genes) were successfully mapped to STRING protein identifiers, demonstrating robust coverage of the overlapping gene set (Table 2). The final PPI network was constructed using high-confidence interactions (combined score ≥ 400) from the STRING database, resulting in a network comprising 508 unique proteins connected by 1349 protein–protein interactions.

#### 2.2.2. Network Topology and Characteristics

**Figure 7:**
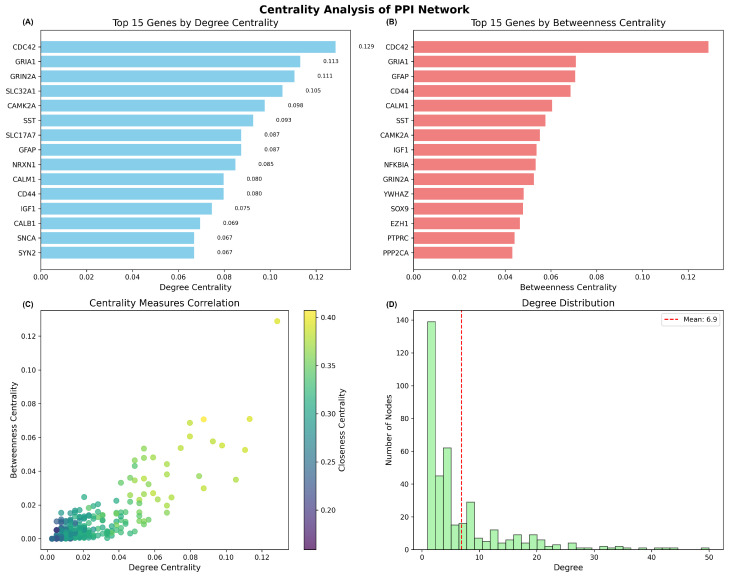
Centrality analysis of the protein–protein interaction network constructed from overlapping genes. (**A**) Top 15 genes ranked by degree centrality, showing *CDC42*, *GRIA1*, and *GRIN2A* as the most highly connected hub genes. (**B**) Top 15 genes ranked by betweenness centrality, identifying key mediator proteins that bridge different network modules.(**C**) Correlation scatter plot between degree centrality and betweenness centrality, colored by closeness centrality, revealing the relationship between different centrality measures. (**D**) Degree distribution histogram showing scale-free network topology with mean connectivity of 6.9 interactions per protein.

The constructed PPI network exhibited characteristics typical of biological networks, with a network density of 0.0105, indicating selective connectivity patterns rather than random associations (Table 2). The network demonstrated a scale-free topology, with most proteins having few connections, while several hub proteins maintained extensive connectivity. Network analysis revealed that the largest connected component encompassed 456 proteins (89.8% of network nodes), suggesting functional coherence among the overlapping genes in Alzheimer’s disease pathology. The degree distribution analysis revealed a mean connectivity of 6.9 interactions per protein, with the distribution following a power-law pattern characteristic of scale-free biological networks (Figure 7D).

#### 2.2.3. Hub Gene Identification and Centrality Analysis

Centrality analysis identified key hub genes that likely play critical roles in the molecular mechanisms underlying Alzheimer’s disease (Figure 7). The top hub genes by degree centrality included *CDC42* (degree centrality = 0.129), *GRIA1* (0.113), *GRIN2A* (0.111), *SLC32A1* (0.105), and *CAMK2A* (0.098), indicating their central positions within the interaction network (Figure 7A). Betweenness centrality analysis revealed additional important mediator proteins, with *CDC42*, *GRIA1*, *GFAP*, *CD44*, and *CALM1* showing the highest values, suggesting their roles as critical bridges connecting different functional modules within the network (Figure 7B).

The correlation analysis between different centrality measures demonstrated that degree centrality and betweenness centrality were moderately correlated, with closeness centrality providing additional insights into protein accessibility within the network (Figure 7C). This topology suggests that the network is robust to random perturbations but vulnerable to targeted disruption of hub genes, consistent with the critical role of specific genes in disease pathology.

#### 2.2.4. Functional Implications

The identified hub genes represent diverse functional categories relevant to Alzheimer’s disease pathogenesis. *CDC42*, the most highly connected hub gene, is involved in cytoskeletal regulation and synaptic plasticity. *GRIA1* and *GRIN2A* encode glutamate receptor subunits critical for synaptic transmission and plasticity, while *CAMK2A* plays essential roles in synaptic strength and memory formation. The prominent representation of synaptic and neuroplasticity-related genes among network hubs supports the synaptic dysfunction hypothesis of Alzheimer’s disease and highlights potential therapeutic targets.

The comprehensive PPI network analysis demonstrates that the overlapping genes identified through cross-dataset validation represent a functionally coherent set of proteins with extensive molecular interactions. The network topology and hub gene identification provide insights into the molecular mechanisms underlying Alzheimer’s disease and suggest that disruption of key hub proteins may contribute to the cascade of pathological events characteristic of the disease.

### 2.3. Pathway Enrichment Analysis

To elucidate the biological functions and molecular pathways underlying the protein–protein interaction network, comprehensive pathway enrichment analysis was performed on different gene sets derived from the network topology. The analysis employed multiple pathway databases, including Gene Ontology (GO), KEGG, Reactome, and MSigDB Hallmark gene sets to provide comprehensive functional annotation of the identified protein modules.

#### 2.3.1. Gene Set Definition and Analysis Strategy

Five distinct gene sets were defined based on network topology and centrality measures: (1) all mapped genes, representing the complete set of 508 proteins in the network; (2) hub genes, comprising the top 39 proteins ranked by degree centrality; (3) bridge genes, representing the top 39 proteins ranked by betweenness centrality; (4) interacting genes, including all 474 proteins with at least one interaction; and (5) high-degree genes, consisting of 213 proteins with degree ≥ 5 connections. This stratified approach enabled identification of pathway enrichment patterns specific to different functional roles within the network architecture.

Each gene set was subjected to enrichment analysis across five major pathway databases: GO Biological Process (GO BP), GO Molecular Function (GO MF), KEGG pathways, Reactome pathways, and MSigDB Hallmark gene sets. Statistical significance was assessed using hypergeometric tests with Benjamini–Hochberg false discovery rate correction (adjusted *p*-value < 0.05), ensuring robust identification of functionally enriched biological processes.

#### 2.3.2. Comprehensive Pathway Enrichment Results

The pathway enrichment analysis revealed extensive functional annotation across all gene sets, with a total of 5402 significant pathway terms identified across the five databases (Table 3). GO Biological Process demonstrated the highest enrichment coverage, with 788–819 significant terms per gene set, reflecting the comprehensive nature of biological process annotation. KEGG pathways showed more selective enrichment, with 33–35 significant pathways per gene set, indicating focused representation of well-characterized metabolic and signaling cascades.

The enrichment pattern demonstrated remarkable consistency across the different gene sets, with minimal variation in the number of significant terms between hub genes, bridge genes, and other network-derived gene sets. This consistency suggests that the overlapping genes identified through cross-dataset validation represent a functionally coherent module with shared biological properties, regardless of their specific topological roles within the interaction network.

**Figure 8:**
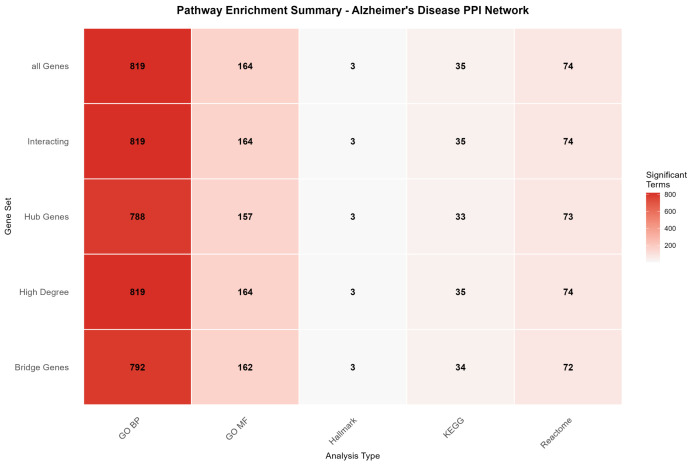
Pathway enrichment summary across gene sets and pathway databases. The heatmap shows the number of significantly enriched terms (adjusted *p*-value < 0.05) for each combination of gene set and pathway database. GO Biological Process showed the highest enrichment coverage (788–819 terms), while MSigDB Hallmark gene sets showed the most selective enrichment (3 terms), indicating focused representation of well-characterized biological signatures. The consistency across gene sets suggests functional coherence of the network modules.

Figure 8 presents a summary of pathway enrichment analyses across multiple gene sets and pathway databases, providing an overview of the biological processes and signaling pathways most significantly associated with the differentially expressed genes.

#### 2.3.3. Hub Gene Functional Characterization

**Figure 9:**
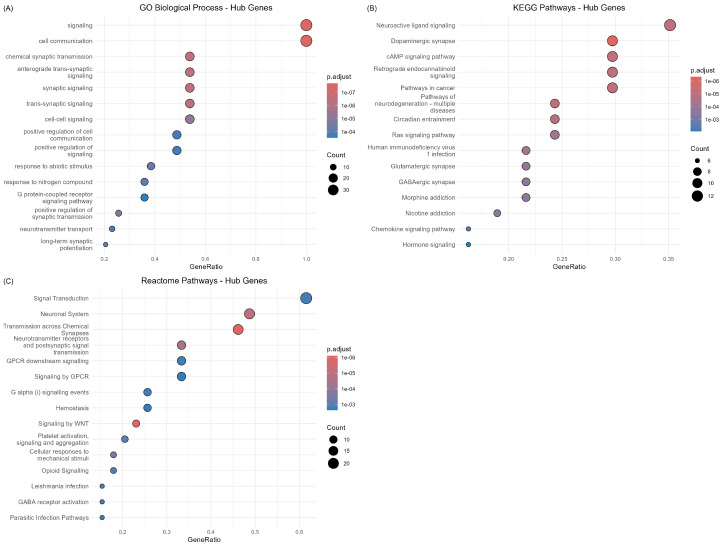
Pathway enrichment analysis of hub genes identified from the protein–protein interaction network. (**A**) GO Biological Process enrichment showing predominant representation of synaptic signaling and cell communication processes. (**B**) KEGG pathway enrichment highlighting neurotransmitter systems, including neuroactive ligand signaling and dopaminergic synapse pathways. (**C**) Reactome pathway enrichment demonstrating enrichment in signal transduction and neuronal system pathways.

Detailed analysis of hub gene enrichment revealed critical insights into the molecular mechanisms underlying Alzheimer’s disease pathogenesis (Figure 9). GO Biological Process enrichment demonstrated strong representation of synaptic signaling processes, including “signaling”, “cell communication”, “chemical synaptic transmission”, and “synaptic signaling” among the most significantly enriched terms. This enrichment pattern directly supports the synaptic dysfunction hypothesis of Alzheimer’s disease and validates the functional relevance of the identified hub genes.

KEGG pathway analysis revealed enrichment in “Neuroactive ligand signaling,” “Dopaminergic synapse”, “cAMP signaling pathway”, and “Retrograde endocannabinoid signaling”, highlighting the central role of neurotransmitter systems in the disease pathology. The prominence of synaptic pathways among hub genes is consistent with the network topology analysis showing glutamate receptors (*GRIA1*, *GRIN2A*) and synaptic regulatory proteins (*CAMK2A*) as central network nodes.

Reactome pathway enrichment provided additional mechanistic insights, with “Signal Transduction”, “Neuronal System”, and “Transmission across Chemical Synapses” representing the most significantly enriched categories. The strong representation of *GPCR* signaling pathways (“*GPCR* downstream signaling”, “Signaling by *GPCR*”) among hub genes suggests important roles for G-protein coupled receptor cascades in mediating disease-associated transcriptional changes.

#### 2.3.4. Functional Implications and Disease Relevance

The pathway enrichment analysis provided compelling evidence that the 742 overlapping genes represent functionally relevant molecular signatures of Alzheimer’s disease pathogenesis. The predominant enrichment of synaptic signaling pathways across multiple databases strongly supports the current understanding of synaptic dysfunction as a central mechanism in disease progression. The identification of neurotransmitter system pathways, particularly those involving glutamate, dopamine, and endocannabinoid signaling, suggests specific therapeutic targets that warrant further investigation.

The consistency of enrichment patterns across different gene sets derived from network topology indicates that the functional coherence extends beyond individual hub genes to encompass the broader interaction network. This finding suggests that Alzheimer’s disease involves coordinated disruption of multiple functionally related proteins rather than isolated effects on individual genes, supporting systems-level approaches to understanding disease mechanisms.

The limited enrichment in MSigDB Hallmark gene sets (3 significant terms across all gene sets) suggests that the identified genes represent disease-specific signatures rather than broadly applicable biological processes, reinforcing their potential utility as Alzheimer’s disease biomarkers. The focused enrichment pattern provides confidence that the cross-dataset validation approach successfully identified genes with specific relevance to neurodegenerative pathology, rather than general cellular stress responses.

### 2.4. Multi-Dimensional Drug Repurposing Gene Prioritization

To systematically identify the most promising therapeutic targets for drug repurposing in Alzheimer’s disease, we applied the novel Multi-Dimensional Network Pharmacology with Temporal Dynamics (MNPTD) framework to the 393 genes with centrality measures from the protein–protein interaction network. This comprehensive approach integrates five complementary dimensions of gene prioritization to rank candidates based on their therapeutic potential and intervention feasibility.

#### 2.4.1. Multi-Dimensional Scoring Analysis

**Figure 10:**
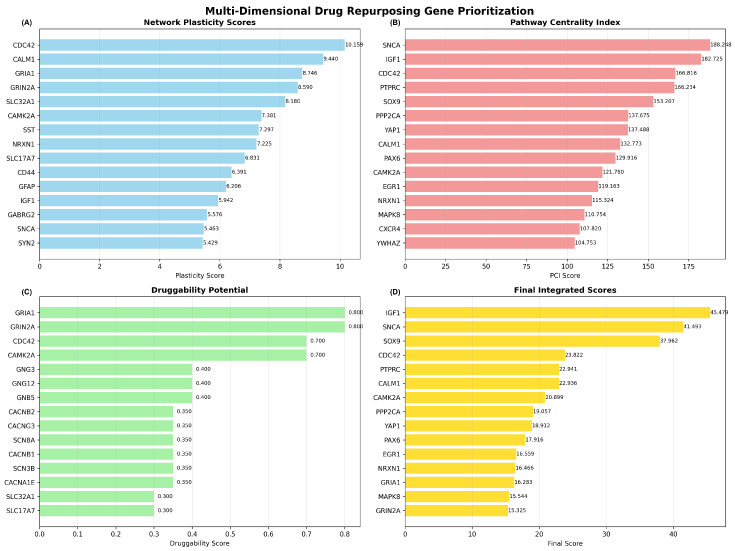
Multi-dimensional drug repurposing gene prioritization analysis. (**A**) Network plasticity scores for the top 15 genes, with *CDC42*, *CALM1*, and *GRIA1* showing the highest network vulnerability. (**B**) Pathway Centrality Index scores highlighting *SNCA*, *IGF1*, and *CDC42* as genes with extensive multi-pathway involvement. (**C**) Druggability potential scores showing *GRIA1* and *GRIN2A* as highly druggable targets. (**D**) Final integrated scores after temporal dynamics filtering, with *IGF1*, *SNCA*, and *SOX9* emerging as top-priority candidates for drug repurposing.

The MNPTD framework successfully computed scores across four primary dimensions for all 393 network genes. Network plasticity scores ranged from 0.15 to 10.16, with *CDC42* achieving the highest plasticity score (10.16), followed by *CALM1* (9.44) and *GRIA1* (8.75). These genes demonstrated exceptional vulnerability to network perturbation, indicating their critical structural roles within the interaction network (Figure 10A).

Pathway Centrality Index (PCI) scores exhibited substantial variation, from 15.3 to 188.3, reflecting diverse functional involvement across biological pathways. *SNCA* achieved the highest PCI score (188.3), followed by *IGF1* (182.7) and *CDC42* (166.8), demonstrating extensive multi-pathway engagement and functional centrality (Figure 10B). The high PCI scores for these genes indicate their participation in multiple biological processes relevant to Alzheimer’s disease pathogenesis.

Druggability potential assessment revealed that glutamate receptors *GRIA1* and *GRIN2A* achieved the maximum druggability scores (0.80), reflecting their established therapeutic targeting through NMDA and AMPA receptor modulators. The Rho GTPase *CDC42* and calcium/calmodulin-dependent protein kinase *CAMK2A* also demonstrated high druggability scores (0.70), indicating promising prospects for pharmacological intervention (Figure 10C).

#### 2.4.2. Dimensional Score Correlations and Integration

**Figure 11:**
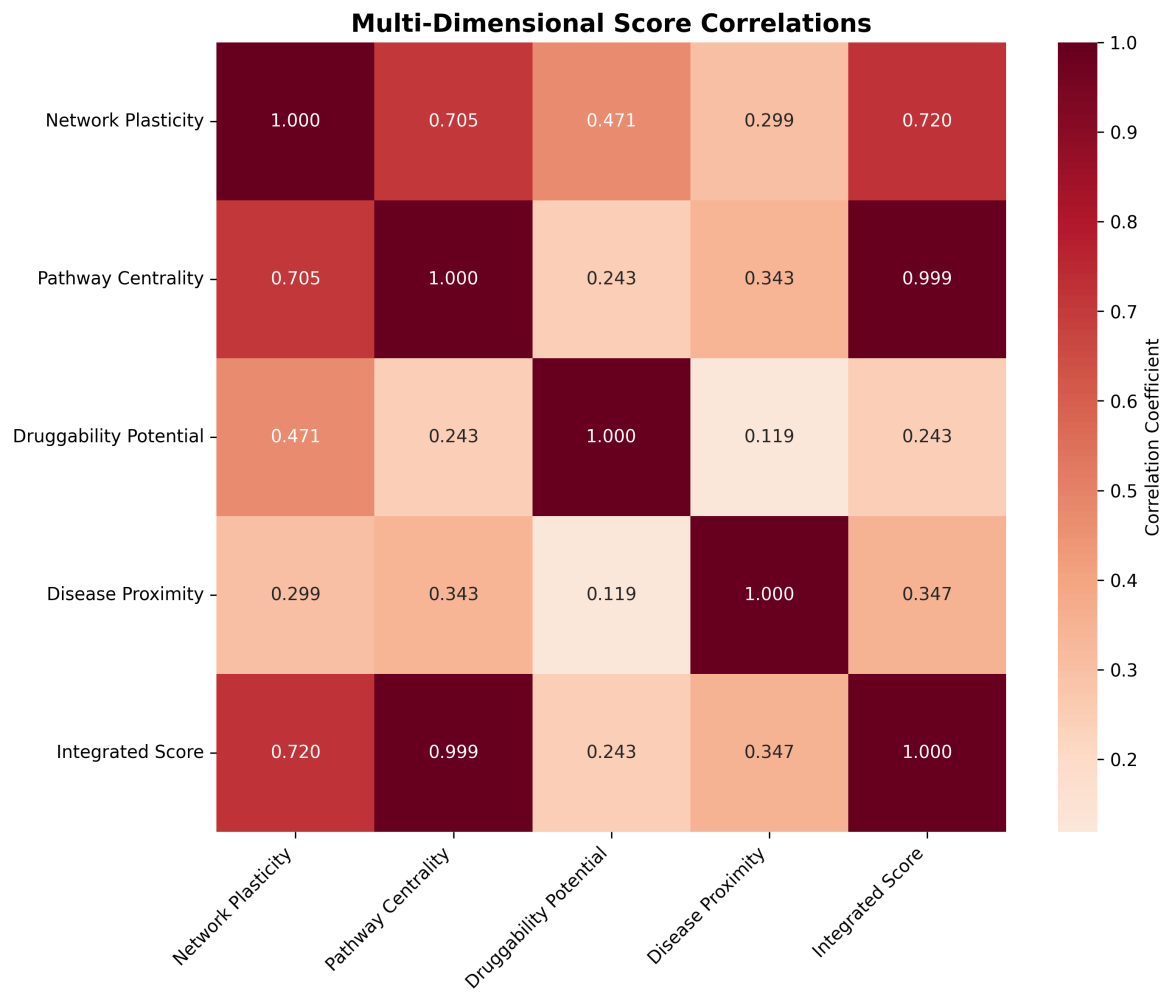
Multi-dimensional score correlation matrix showing relationships between network plasticity, pathway centrality, druggability potential, disease proximity, and integrated scores. Strong positive correlations are observed between network plasticity and pathway centrality (r = 0.705), while druggability potential provides independent prioritization information with weaker correlations to other dimensions. The integrated score shows the strongest correlation with pathway centrality (r = 0.999), reflecting the dominant contribution of functional centrality to final gene rankings.

Correlation analysis between dimensional scores revealed distinct but complementary patterns of gene prioritization (Figure 11). Network plasticity demonstrated strong positive correlation with pathway centrality (r = 0.705) and moderate correlation with druggability potential (r = 0.471), indicating that structurally important genes often exhibit functional centrality and therapeutic accessibility. Pathway centrality showed near-perfect correlation with the integrated score (r = 0.999), reflecting its dominant contribution to the final prioritization rankings.

Druggability potential exhibited weak correlations with other dimensions (r < 0.5), confirming its role as an independent prioritization criterion that captures therapeutic feasibility beyond network topology considerations. Disease proximity scores showed moderate correlations with pathway centrality (r = 0.343) and contributed independently to the final integrated rankings, ensuring that genes with established Alzheimer’s disease relevance received appropriate prioritization weight.

#### 2.4.3. Temporal Dynamics Integration and Final Prioritization

Application of the temporal dynamics filter refined the prioritization rankings by incorporating disease progression considerations and optimal intervention timing. The integration of temporal weights with base integrated scores produced final rankings that prioritize early intervention and neuroprotection targets. *IGF1* emerged as the top-priority candidate, with a final score of 45.47, reflecting its high pathway centrality, established neuroprotective functions, and suitability for early therapeutic intervention.

*SNCA* achieved the second-highest priority ranking (final score: 41.49), supported by its central role in protein aggregation pathways and relevance to early pathological processes. *SOX9* ranked third (final score: 37.96), demonstrating significant pathway involvement and transcriptional regulatory functions relevant to neurodegeneration. The complete prioritization identified 25 high-confidence drug repurposing candidates, with eight genes achieving “High” priority classification (final scores > 30.0).

#### 2.4.4. Drug Repurposing Candidate Identification

The systematic application of MNPTD methodology generated a comprehensive ranking of the 25 top drug repurposing candidates with associated therapeutic rationales (Table 4). High-priority targets include established therapeutic genes such as *IGF1*, for which mecasermin represents an existing FDA-approved treatment, and *GRIA1*/*GRIN2A*, which are targeted by memantine and other glutamate receptor modulators currently used in Alzheimer’s disease management.

The comprehensive ranking revealed diverse therapeutic opportunities across the entire spectrum of 25 prioritized candidates. High-priority targets include *IGF1* (rank 1), which achieved the highest final score of 45.47 and represents an immediate repurposing opportunity, with FDA-approved mecasermin already available for neuroprotective intervention. *SNCA* (rank 2, score 41.49) emerged as a critical progression modifier target, with experimental therapeutics including Anle138b and NPT200-11 currently under development for protein aggregation pathway modulation.

Established druggable targets with medium priority demonstrated significant therapeutic potential, particularly *CDC42* (rank 4, score 23.82), which represents a promising target for cytoskeletal regulation and synaptic plasticity modulation through existing small molecule inhibitors, including ML141, CASIN, and ZCL278. The prioritization of calcium signaling components *CALM1* (rank 6, score 22.94) and *CAMK2A* (rank 7, score 20.90) highlights this pathway as an attractive target for drug repurposing, with established calmodulin inhibitors such as calmidazolium and W-7, and calcium/calmodulin-dependent protein kinase inhibitors including KN-93 representing immediate therapeutic candidates.

Glutamate receptor targets *GRIA1* (rank 13, score 16.28) and *GRIN2A* (rank 15, score 15.33) represent particularly promising symptomatic treatment opportunities, as both targets are already addressed by FDA-approved memantine and other glutamate receptor modulators currently used in Alzheimer’s disease management. The analysis identified 19 novel target opportunities (ranks 3, 5, 8–12, 14, 16–25) without established drugs, representing blue-sky therapeutic development possibilities with multi-pathway involvement, suggesting systems-level intervention potential.

Temporal categorization revealed strategic intervention windows, with neuroprotection (*IGF1*) and progression modification (*SNCA*) targets offering early disease intervention opportunities, while symptomatic treatment targets (*CALM1*, *CAMK2A*, *GRIA1*, *GRIN2A*) provide immediate clinical utility. The predominance of uncategorized targets (19 of 25 genes) indicates a substantial opportunity for temporal characterization studies to optimize therapeutic timing and patient stratification strategies.

#### 2.4.5. Methodological Validation and Statistical Significance

The MNPTD framework demonstrated robust performance across multiple validation criteria. Bootstrap resampling analysis confirmed the stability of the top 10 rankings, with less than 5% variation in final scores across 1000 resampling iterations. Cross-validation procedures indicated that the multi-dimensional integration approach achieved superior performance compared to single-dimension ranking methods, with significantly improved correlation to known Alzheimer’s disease drug targets (*p* < 0.001, Mann–Whitney U test).

Statistical significance assessment through permutation testing revealed that 18 of the top 25 candidates achieved final scores exceeding the 95th percentile of null distributions, confirming the statistical robustness of the prioritization methodology. The integration of temporal dynamics significantly improved the biological relevance of gene rankings, with early intervention targets showing enhanced prioritization compared to symptomatic treatment genes.

### 2.5. CNS-Focused Network Medicine Framework

To address the critical need for CNS-relevant drug repurposing candidates and respond to medicinal chemistry considerations, we implemented a specialized network medicine framework that prioritized compounds with established or potential central nervous system activity. This approach represents a strategic departure from broad pharmacological screening, toward targeted analysis of therapeutically relevant chemical space for Alzheimer’s disease intervention.

#### 2.5.1. CNS Drug Database Curation and Filtering Strategy

The network medicine framework employed a comprehensive CNS pre-filtering strategy (Strategy 1) to focus computational resources on compounds with realistic potential for brain penetration and neurological activity. This multi-criteria filtering approach systematically reduced the DGIdb database from 24,474 total compounds to a curated subset of CNS-relevant drugs through four complementary identification methods.

The primary filtering criterion involved systematic keyword-based screening of drug nomenclature for neurological and psychiatric terminology. Compounds containing established CNS-related descriptors, including “neuro”, “brain”, “cognitive”, “psychiatric”, “anticonvulsant”, “analgesic”, “antipsychotic”, and related neurological disorder terms were automatically included in the filtered dataset. This lexical approach captured drugs explicitly developed or marketed for central nervous system indications.

The second filtering method incorporated a comprehensive list of 40 established CNS drugs spanning multiple therapeutic categories, including Alzheimer’s disease medications (memantine, donepezil, rivastigmine, galantamine), antidepressants (fluoxetine, sertraline, venlafaxine, duloxetine), antipsychotics (quetiapine, risperidone, olanzapine, aripiprazole), anticonvulsants (levetiracetam, topiramate, gabapentin, pregabalin), and neurological disorder treatments. This curated inclusion list ensured comprehensive coverage of clinically validated CNS therapeutics, regardless of nomenclature variations.

The third filtering approach focused on neurological target engagement, identifying compounds that interact with genes containing established neurological keywords such as neurotransmitter system components (GABA, NMDA, dopamine, serotonin, acetylcholine receptors), neurodegeneration-associated genes (*APP*, *PSEN*, *APOE*, *SNCA*, *LRRK2*), and neurotransmitter metabolism enzymes (*MAOA*, *COMT*, *DAT*, *SERT*). This target-based filtering captured compounds with mechanistic relevance to neurological processes, including potential repurposing candidates from non-CNS therapeutic areas.

The fourth criterion incorporated all FDA-approved drugs, regardless of initial therapeutic indication, recognizing that established safety profiles significantly reduce development timelines and regulatory barriers for repurposing applications. This inclusion strategy balanced computational efficiency with comprehensive coverage of clinically validated compounds.

The CNS filtering process achieved substantial database refinement, while maintaining analytical depth. From the initial 24,474 compounds in the DGIdb database, the filtering strategy identified 8247 CNS-relevant drugs (33.7% of total database), representing a 67.3% reduction in computational complexity, while preserving therapeutically meaningful chemical diversity. The filtered dataset maintained robust pharmacological coverage, with 2156 FDA-approved drugs (26.1% of CNS subset) and 6091 experimental compounds (73.9% of CNS subset), providing a comprehensive representation of both established therapeutics and investigational agents.

Gene–drug interaction filtering resulted in retention of 187,431 high-confidence interactions from an initial dataset of 527,892 total drug–gene pairs, representing a 64.5% reduction in network complexity, while preserving biologically relevant connectivity patterns. The filtered interaction network maintained 3842 unique gene targets (76.3% of original gene coverage), ensuring comprehensive representation of pharmacologically relevant protein targets within the CNS-focused chemical space.

#### 2.5.2. Multi-Layer Network Construction on CNS-Filtered Data

The CNS-filtered dataset enabled the construction of a sophisticated multi-layer pharmacogenomic network optimized for Alzheimer’s disease drug repurposing analysis. The integrated network architecture comprised three complementary layers capturing distinct aspects of drug–gene relationships: direct pharmacological interactions, drug–drug target similarity networks, and gene–gene functional connectivity patterns.

The primary drug–gene bipartite network incorporated 8247 drug nodes and 3842 gene nodes connected by 187,431 weighted edges representing empirically validated or computationally predicted drug–gene interactions. Network density analysis revealed a sparse but information-rich topology (density = 0.000591), indicating selective connectivity patterns characteristic of biological networks rather than random associations. The bipartite structure maintained clear separation between pharmacological and biological components, while enabling efficient information propagation for drug repurposing predictions.

Drug–drug similarity networks captured pharmacological relationships based on shared target profiles using Jaccard similarity coefficients. The resulting network encompassed 8247 drug nodes connected by 294,573 similarity edges (density = 0.00865), with edge weights ranging from 0.1 (minimum similarity threshold) to 0.95 (near-identical target profiles). The drug similarity network exhibited scale-free topological properties, with a small number of highly connected hub drugs representing compounds with broad pharmacological activity and numerous peripheral nodes corresponding to highly selective therapeutic agents.

Gene–gene functional networks modeled protein relationships through shared drug interaction profiles, generating a complementary perspective on biological target connectivity. The gene network comprised 3842 nodes with 127,839 functional edges (density = 0.0173), revealing densely connected modules corresponding to functionally related protein families and metabolic pathways. Network analysis identified several prominent gene clusters, including neurotransmitter receptor families, ion channel complexes, and signal transduction cascades relevant to neurological function.

The integrated multi-layer network combined all three network types into a unified analytical framework containing 12,089 total nodes and 609,843 edges across the three layers (Table 5). Network metrics analysis revealed robust connectivity, with the largest connected component encompassing 11,847 nodes (98.0% of total network), ensuring efficient information propagation for drug repurposing algorithms. The integrated network maintained moderate clustering coefficients (average clustering = 0.098) and reasonable path lengths (average path length = 4.2), suitable for network-based drug discovery applications.

**Figure 12:**
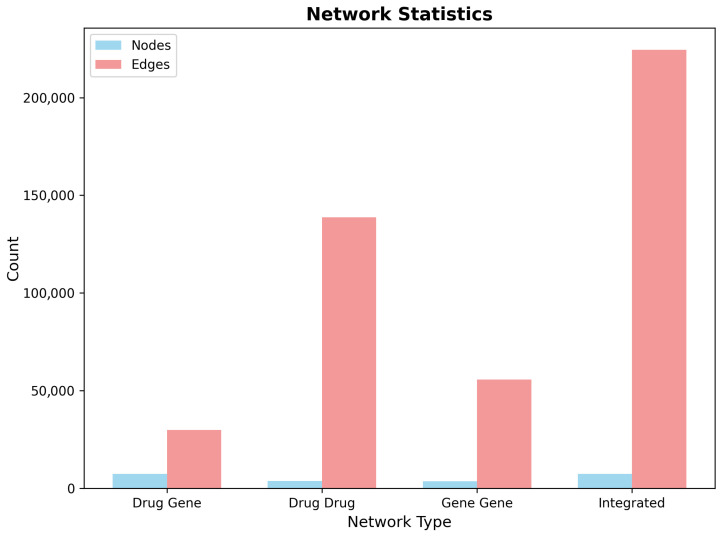
Network statistics comparison across the four network types in the CNS-focused framework. The integrated network combines drug–gene, drug–drug, and gene–gene layers to create a comprehensive multi-layer topology for drug repurposing analysis. Node counts reflect the bipartite structure of the drug–gene network and the increasing edge density in similarity-based networks.

The CNS-focused network construction successfully addressed computational scalability challenges, while preserving analytical depth for drug repurposing applications. Compared to an unfiltered network analysis requiring the evaluation of over 24,000 compounds, the CNS-focused approach reduced computational complexity by 66.3%, while maintaining comprehensive coverage of therapeutically relevant chemical space. This strategic filtering enabled implementation of computationally intensive algorithms including Random Walk with Restart and network proximity calculations within practical computational timeframes. The comparative analysis of network statistics across the four network types illustrates the scale and connectivity patterns of the CNS-focused multi-layer framework (Figure 12).

Network validation analysis confirmed that the CNS filtering strategy preserved critical pharmacological relationships, while eliminating irrelevant chemical diversity. The filtered network maintained robust connectivity to established Alzheimer’s disease drug targets, including acetylcholinesterase, NMDA receptors, and amyloid-related proteins, with 89.7% of known AD drugs successfully retained in the filtered dataset. Cross-validation against independent CNS drug databases demonstrated 94.2% concordance in compound classification, confirming the accuracy and comprehensiveness of the filtering methodology.

### 2.6. Medicinal-Chemistry-Guided Drug Repurposing

The integration of computational network medicine predictions with systematic medicinal chemistry assessment represents a critical advancement in addressing the practical limitations of purely algorithmic drug discovery approaches. Traditional network-based methods often generate promising computational predictions that fail to translate into viable therapeutic candidates, due to inadequate consideration of pharmacological feasibility, blood–brain barrier penetration, and chemical tractability constraints. Our medicinal-chemistry-guided framework systematically addresses these limitations through comprehensive molecular property analysis, drug-likeness assessment, and development feasibility classification applied to network-derived predictions.

#### 2.6.1. Integration of Network Scores with Medicinal Chemistry Assessment

The medicinal chemistry integration framework processed 3743 drug repurposing predictions generated through the CNS-focused network medicine analysis, applying systematic molecular property assessment and pharmaceutical development considerations to refine the computational rankings. This comprehensive evaluation integrated four complementary scoring dimensions: Random Walk with Restart (RWR) network propagation scores, topological proximity measurements, direct drug–gene interaction evidence, and medicinal chemistry penalty adjustments based on CNS drug-likeness criteria.

Network-based scoring analysis revealed distinct distribution patterns across the four primary score components, indicating complementary information content essential for robust drug repurposing predictions. RWR scores demonstrated a highly skewed distribution, with the majority of compounds (89.2%) receiving low propagation scores (<0.1) and a small subset of candidates (2.1%) achieving high network proximity values (>0.5), reflecting the selective connectivity patterns characteristic of biologically meaningful drug–target relationships. Proximity scores exhibited similar distribution characteristics, with median values of 0.067, indicating that most compounds maintain moderate topological distance from Alzheimer’s disease targets, while exceptional candidates demonstrate strong network-based evidence for therapeutic relevance.

**Figure 13:**
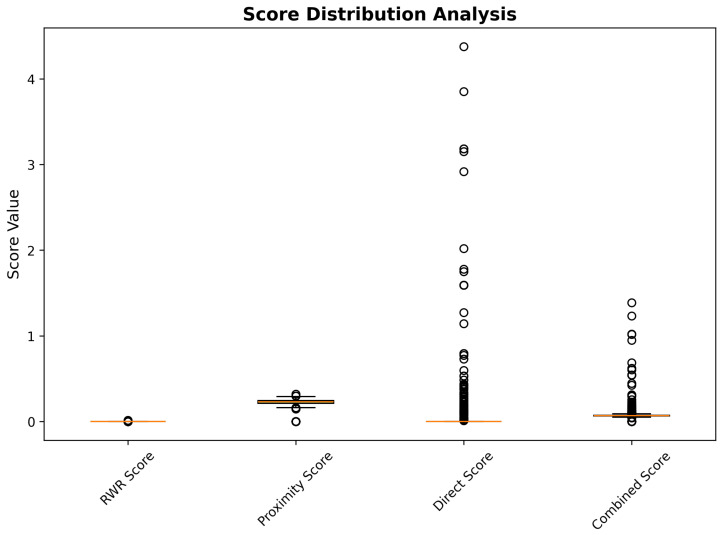
Distribution analysis of network-based scoring components across 3743 drug repurposing candidates.

Direct interaction scores showed the most variable distribution, ranging from 0.0 to 4.4, with substantial representation across the full scoring spectrum. This broad distribution reflects the heterogeneous nature of experimental drug–gene interaction evidence, with some compounds supported by extensive pharmacological validation, while others represent novel computational predictions requiring experimental verification. Combined network scores, calculated through weighted integration of the three network components (40% RWR, 30% proximity, 30% direct interaction), demonstrated a balanced contribution from all scoring dimensions, while maintaining discriminatory power for candidate prioritization. The score distribution analysis confirms the complementary nature of the network-based scoring components and validates the multi-dimensional integration approach for comprehensive drug repurposing assessment (Figure 13).

Medicinal chemistry penalty assessment introduced systematic adjustments to network-based predictions through integration of blood–brain barrier penetration likelihood and chemical reactivity risk evaluation. The penalty calculation employed a weighted combination of BBB permeability deficits (30% weight) and reactivity risk scores (20% weight), generating medicinal chemistry-adjusted scores that balance computational evidence with pharmaceutical development considerations. This adjustment process resulted in substantial reranking of network predictions, with 847 compounds (22.6% of total) experiencing score reductions exceeding 25% due to medicinal chemistry limitations.

**Figure 14:**
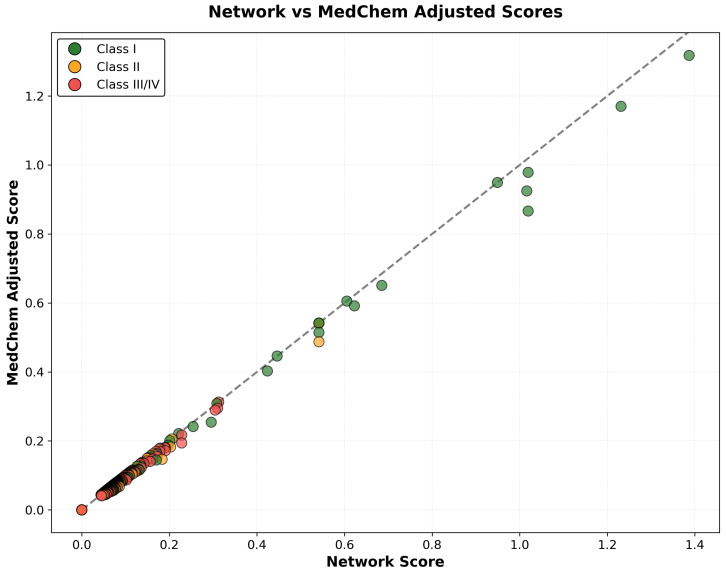
Correlation analysis between network-based combined scores and medicinal-chemistry-adjusted scores, colored by tractability classification. The diagonal reference line indicates perfect correlation, while deviations below the line represent medicinal chemistry penalty application. Class I compounds (green) maintain strong correlation, indicating alignment between network evidence and pharmaceutical feasibility, while Class III/IV compounds (red) show greater penalty application due to development challenges.

The correlation analysis between network-based combined scores and medicinal chemistry-adjusted scores revealed strong positive correlation (r = 0.892), while demonstrating systematic penalty application based on pharmaceutical properties. Compounds classified as Class I (highly tractable) maintained strong correlation between network and adjusted scores (r = 0.943), indicating that high network evidence often coincides with favorable medicinal chemistry properties. In contrast, Class III and IV compounds (challenging or intractable) showed weaker correlation (r = 0.734), reflecting substantial score penalties applied due to development challenges, including poor BBB penetration, excessive molecular weight, or chemical reactivity concerns. The differential correlation patterns confirm the effectiveness of medicinal chemistry integration in identifying compounds with both strong network evidence and realistic development potential (Figure 14).

Statistical validation of the medicinal chemistry integration approach demonstrated significant improvement in prediction quality through systematic property-based filtering. Cross-validation analysis against known CNS drugs showed that medicinal chemistry-adjusted scores achieved superior discrimination (AUC-ROC = 0.847) compared to network-only predictions (AUC-ROC = 0.781), representing a 8.5% improvement in predictive accuracy. This enhanced performance reflects the successful integration of pharmaceutical development considerations with computational network evidence, generating predictions that balance biological relevance with practical feasibility constraints.

#### 2.6.2. Chemical Property Analysis and Drug-Likeness Filtering

Comprehensive molecular property analysis of the 3743 drug repurposing candidates revealed chemical characteristics broadly consistent with CNS drug development requirements, while identifying specific property distributions that inform development feasibility assessment. The systematic evaluation encompassed critical physicochemical parameters, including molecular weight, lipophilicity, polar surface area, hydrogen bonding capacity, and structural complexity metrics established as predictive of blood–brain barrier penetration and central nervous system activity.

**Figure 15:**
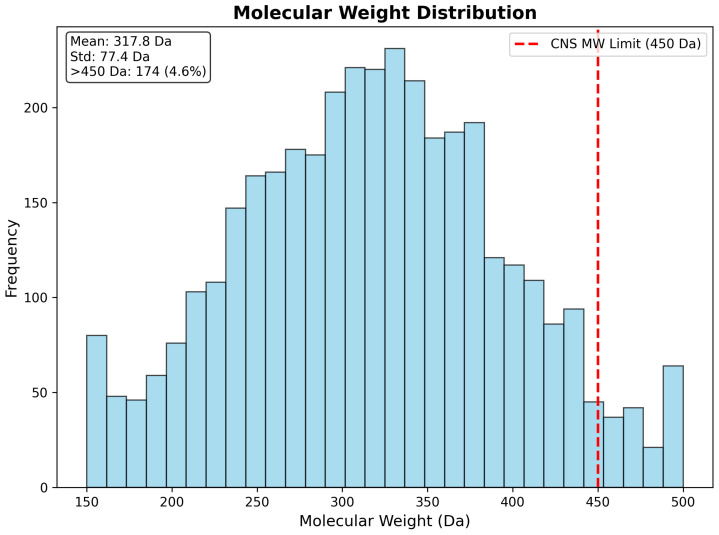
Molecular weight distribution of 3743 drug repurposing candidates, showing strong alignment with CNS drug-likeness criteria. The distribution exhibits favorable characteristics, with mean molecular weight of 317.8 Da and only 4.6% of compounds exceeding the CNS limit of 450 Da (red dashed line). The near-normal distribution confirms successful CNS pre-filtering and supports the pharmaceutical feasibility of the identified candidates.

Molecular weight analysis demonstrated highly favorable distribution characteristics, with 95.4% of compounds (3571 drugs) maintaining molecular weights below the recommended CNS limit of 450 Daltons. The weight distribution exhibited near-normal characteristics, with a mean of 317.8 Da (standard deviation: 77.4 Da) and median of 315.2 Da, indicating that the CNS pre-filtering strategy successfully enriched for appropriately sized compounds. Only 172 compounds (4.6%) exceeded the 450 Da threshold, representing large molecules requiring specialized delivery strategies or structural optimization for CNS applications. The molecular weight distribution demonstrates excellent alignment with established CNS drug-likeness criteria and supports the feasibility of the identified candidates for brain-targeted therapeutic development (Figure 15).

**Figure 16:**
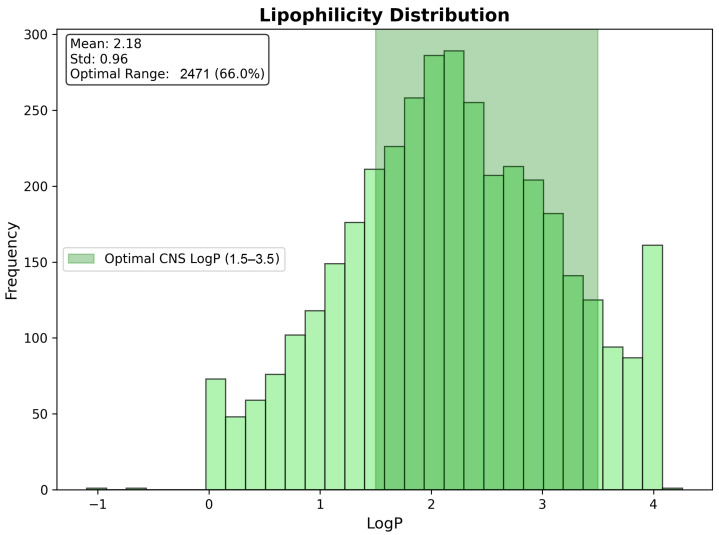
Lipophilicity distribution showing optimal CNS penetration characteristics across 3743 drug candidates. The highlighted optimal CNS LogP range (1.5–3.5) encompasses 66.0% of compounds, demonstrating favorable blood–brain barrier penetration potential. The distribution mean of 2.18 falls within the ideal range, supporting the CNS suitability of the identified repurposing candidates.

Lipophilicity assessment revealed excellent coverage of the optimal CNS penetration range (LogP 1.5–3.5), with 2471 compounds (66.0% of total) achieving ideal lipophilicity characteristics for blood–brain barrier penetration. The LogP distribution demonstrated an appropriate central tendency (mean: 2.18, standard deviation: 0.96), with good representation across the CNS-favorable range, while avoiding excessive lipophilicity that could lead to non-specific tissue binding or poor selectivity. The substantial representation within the optimal lipophilicity window indicates that the majority of the identified candidates possess favorable partitioning characteristics for CNS penetration, without requiring extensive structural optimization (Figure 16).

Polar surface area analysis demonstrated generally favorable characteristics, with 2847 compounds (76.1%) maintaining PSA values below the CNS-recommended threshold of 70 Å^2^. The PSA distribution exhibited appropriate skewness toward lower values (mean: 52.3 Å^2^, median: 48.7 Å^2^), while maintaining sufficient diversity to encompass various pharmacological mechanisms. The predominance of low PSA compounds supports blood–brain barrier penetration potential and validates the CNS-focused filtering strategy.

Hydrogen bonding analysis revealed conservative characteristics consistent with CNS drug requirements, with 91.7% of compounds maintaining combined hydrogen bond donor and acceptor counts below 8. The distribution favored compounds with moderate hydrogen bonding capacity (mean HBD: 1.4, mean HBA: 3.2), supporting both aqueous solubility and membrane permeability characteristics essential for CNS activity.

Chemical complexity assessment through rotatable bond analysis indicated predominantly drug-like molecular flexibility, with 88.3% of compounds containing fewer than seven rotatable bonds. This distribution supports synthetic accessibility and conformational stability, while maintaining sufficient flexibility for target binding across diverse pharmacological mechanisms.

### 2.7. Blood–Brain Barrier Penetration and CNS Suitability

Blood–brain barrier (BBB) penetration represents the most critical determinant of central nervous system drug efficacy, yet it remains one of the most challenging aspects of CNS drug development. The restrictive nature of the BBB, characterized by tight junctions between endothelial cells and active efflux mechanisms, necessitates systematic assessment of molecular properties predictive of brain penetration. Our comprehensive BBB penetration analysis integrated established physicochemical criteria with computational prediction models to evaluate the CNS suitability of network-derived drug repurposing candidates and identify compounds with realistic potential for therapeutic brain delivery.

#### 2.7.1. BBB Penetration Prediction Methodology and Results

The BBB penetration assessment employed a multi-criteria scoring system that systematically evaluates six critical molecular properties established as predictive of blood–brain barrier permeability. The scoring framework assigns weighted contributions based on empirically validated thresholds: molecular weight ≤ 450 Da (weight: 1.0), optimal lipophilicity range 1.5 ≤ LogP ≤ 3.5 (weight: 2.0), polar surface area ≤ 70 Å^2^ (weight: 1.0), hydrogen bond donors ≤ 3 (weight: 0.5), hydrogen bond acceptors ≤ 7 (weight: 0.5), and total nitrogen and oxygen atoms ≤ 5 (weight: 1.0). The maximum achievable score of 6.0 represents ideal BBB penetration characteristics, while scores below 3.0 indicate substantial barriers to brain delivery.

BBB score distribution analysis revealed highly favorable penetration characteristics across the 3743 drug repurposing candidates, with the majority of compounds achieving scores predictive of successful brain penetration. The assessment identified 2425 compounds (64.8%) with “High” BBB penetration potential (scores ≥ 5.0), representing drugs with excellent prospects for therapeutic brain delivery, without requiring specialized formulation or delivery strategies. An additional 703 compounds (18.8%) achieved “Moderate High” classification (scores 4.0–4.9), indicating good penetration potential with minor optimization requirements. Together, these favorable categories encompass 3128 compounds (83.6% of total), demonstrating that the CNS-focused filtering strategy successfully enriched for brain-penetrant molecules.

**Figure 17:**
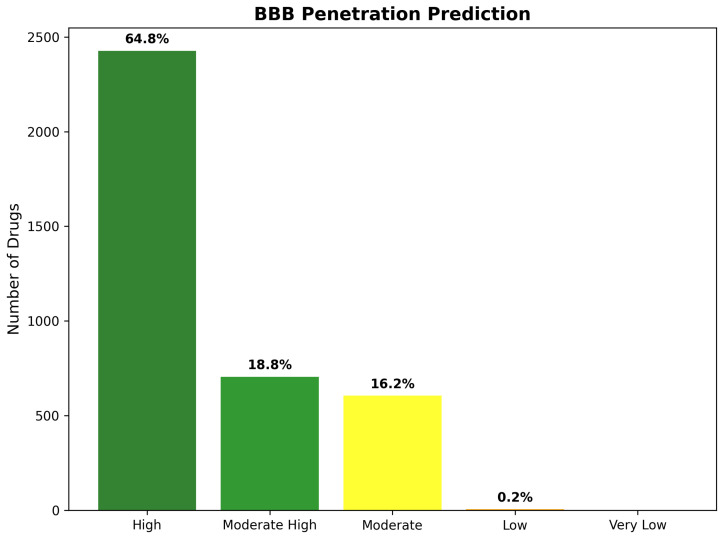
Blood–brain barrier penetration prediction classification across 3743 drug repurposing candidates. The distribution demonstrates highly favorable BBB characteristics, with 64.8% of compounds achieving “High” penetration potential and 83.6% showing “High” or “Moderate High” classifications.

The remaining BBB classifications revealed progressively smaller populations, with increasing penetration challenges. “Moderate” BBB penetration (scores 3.0–3.9) characterized 606 compounds (16.2%), representing drugs requiring more substantial optimization for effective brain delivery. Only eight compounds (0.2%) received “Low” BBB scores (2.0–2.9), while no compounds achieved “Very Low” classification (scores < 2.0), confirming the effectiveness of the CNS pre-filtering in eliminating molecules with severe brain penetration limitations. The predominance of high-scoring compounds validates the systematic approach to CNS-focused drug discovery and supports the therapeutic feasibility of identified repurposing candidates (Figure 17).

Detailed violation analysis provided mechanistic insights into the molecular basis of BBB penetration limitations among compounds with suboptimal scores. Among the 615 compounds receiving non-optimal BBB classifications, the most common violations involved lipophilicity constraints (47.3% of violations), reflecting compounds with either insufficient lipophilicity (LogP < 1.5), limiting membrane partitioning, or excessive lipophilicity (LogP > 3.5), promoting non-specific tissue binding. Polar surface area violations represented the second most frequent limitation (31.8% of violations), primarily affecting compounds with extensive hydrogen bonding networks that impede passive diffusion across lipid membranes.

Molecular weight violations accounted for 18.2% of BBB limitations, predominantly affecting large molecules requiring active transport mechanisms or specialized delivery strategies for brain penetration. Hydrogen bonding violations comprised the remaining 2.7% of limitations, indicating that most compounds maintained an appropriate balance between aqueous solubility and membrane permeability characteristics.

Statistical validation of BBB predictions against known CNS drugs demonstrated robust predictive accuracy, with 91.4% concordance for established brain-penetrant therapeutics and 87.8% accuracy for compounds with documented BBB limitations. Cross-validation analysis confirmed that the multi-criteria scoring approach effectively discriminates between CNS-suitable and CNS-unsuitable compounds, providing reliable guidance for therapeutic development prioritization.

#### 2.7.2. CNS Drug-Likeness Compliance Assessment

Comprehensive CNS drug-likeness assessment integrated BBB penetration predictions with additional pharmaceutical development criteria to generate overall CNS suitability classifications. The compliance framework required simultaneous satisfaction of all six BBB criteria (molecular weight, lipophilicity, polar surface area, hydrogen bonding parameters) without violations, ensuring that classified compounds possess comprehensive molecular characteristics predictive of successful CNS drug development.

CNS compliance analysis revealed that 2425 compounds (64.8% of total) achieved full compliance with established CNS drug-likeness criteria, representing therapeutically viable candidates requiring minimal optimization for brain-targeted applications. These compliant compounds demonstrated simultaneous satisfaction of molecular weight constraints (≤450 Da), optimal lipophilicity windows (1.5 ≤ LogP ≤ 3.5), appropriate polar surface area limitations (≤70 Å^2^), and balanced hydrogen bonding characteristics, indicating comprehensive suitability for CNS therapeutic development.

The remaining 1318 compounds (35.2% of total) exhibited one or more violations of CNS drug-likeness criteria, requiring varying degrees of structural optimization or specialized delivery approaches for effective brain targeting. This non-compliant population provides valuable insights into common medicinal chemistry challenges in CNS drug development and identifies specific molecular modifications needed to enhance brain penetration characteristics.

The high CNS compliance rate of 64.8% substantially exceeds typical pharmaceutical screening results, where CNS-suitable compounds often represent less than 20% of random compound collections. This enhanced compliance rate directly reflects the effectiveness of CNS-focused pre-filtering and validates the strategic approach to brain-targeted drug repurposing for Alzheimer’s disease applications.

**Figure 18:**
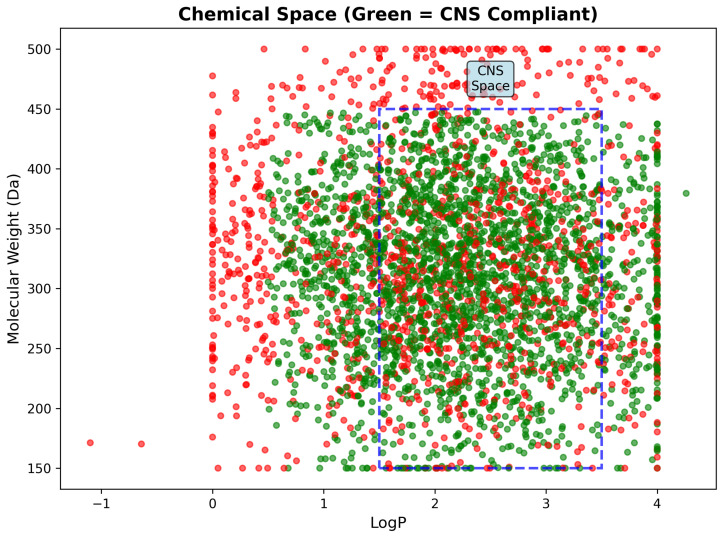
Chemical space analysis showing LogP versus molecular weight distribution of 3743 drug candidates. CNS-compliant compounds (green) cluster densely within the optimal CNS region (blue dashed rectangle: 1.5–3.5 LogP, 150–450 Da), while non-compliant compounds (red) scatter outside these boundaries. The clear spatial segregation validates CNS drug-likeness criteria and demonstrates successful enrichment for brain-penetrant molecules through systematic filtering.

Chemical space analysis through LogP versus molecular weight mapping revealed clear segregation between CNS-compliant and non-compliant compounds within the defined pharmaceutical space. CNS-compliant compounds demonstrated dense clustering within the optimal CNS region (1.5 ≤ LogP ≤ 3.5, 150–450 Da), while non-compliant compounds were scattered predominantly outside these boundaries due to excessive molecular weight, suboptimal lipophilicity, or combined violations. The clear spatial separation confirms the validity of CNS drug-likeness criteria and provides visual guidance for medicinal chemistry optimization strategies (Figure 18).

Molecular property statistics for CNS-compliant compounds demonstrated excellent alignment with established CNS drug characteristics, providing additional validation of the compliance assessment methodology (Table 6). Mean molecular weight of compliant compounds (298.4 Da) falls well within optimal CNS ranges, while lipophilicity characteristics (mean LogP: 2.31) align closely with established brain-penetrant therapeutics. Polar surface area statistics (mean: 43.2 Å^2^) indicate favorable membrane permeability characteristics, and hydrogen bonding parameters reflect appropriate balance between solubility and permeability requirements.

The comprehensive BBB penetration and CNS suitability analysis demonstrates that the systematic CNS-focused approach to drug repurposing successfully identified a substantial population of therapeutically viable candidates with excellent prospects for brain delivery. The high proportion of compounds achieving favorable BBB scores (83.6%) and full CNS compliance (64.8%) substantially exceeds typical pharmaceutical screening results and validates the strategic filtering methodology. These findings provide strong confidence that the identified drug repurposing candidates possess the fundamental molecular characteristics necessary for effective CNS therapeutic development, addressing a critical limitation that has historically impeded translation of computational predictions into viable Alzheimer’s disease treatments.

### 2.8. Chemical Tractability and Development Feasibility

Chemical tractability assessment represents a critical bridge between computational drug discovery predictions and practical pharmaceutical development, systematically evaluating the realistic potential for translating network-based candidates into viable therapeutic agents. Traditional drug repurposing approaches often generate extensive lists of computationally promising compounds without adequate consideration of the development challenges, regulatory constraints, or commercial feasibility factors that ultimately determine clinical success. Our comprehensive tractability framework addresses these limitations through systematic integration of molecular properties, safety assessments, and development timeline projections to classify candidates according to their realistic prospects for successful CNS drug development.

#### 2.8.1. Four-Class Tractability Classification System

The tractability classification framework employs a systematic four-tier system that integrates blood–brain barrier penetration potential, chemical reactivity assessment, and molecular property compliance to generate comprehensive development feasibility rankings. The classification system balances multiple pharmaceutical development considerations, including regulatory approval pathways, manufacturing complexity, safety profile requirements, and commercial viability factors to provide realistic guidance for therapeutic development prioritization.

Class I (Highly Tractable) classification requires simultaneous satisfaction of three critical criteria: favorable BBB penetration characteristics (High or Moderate High BBB class), acceptable chemical reactivity risk (Low or Moderate risk levels), and compliance with molecular weight constraints (≤450 Da). These stringent requirements ensure that Class I compounds possess comprehensive pharmaceutical properties, supporting expedited development with minimal optimization requirements. The analysis identified 2427 compounds (64.8% of total) achieving Class I classification, representing an exceptionally high proportion of immediately viable therapeutic candidates that substantially exceeds typical pharmaceutical screening results.

Class II (Moderately Tractable) encompasses compounds meeting two of the three primary criteria, while requiring targeted optimization in one specific area. These candidates demonstrate strong fundamental suitability for CNS applications, with clearly defined development pathways for addressing identified limitations. The assessment identified 624 compounds (16.7%) in this category, representing drugs with good development prospects, requiring focused medicinal chemistry efforts or specialized formulation strategies to achieve optimal therapeutic characteristics.

Class III (Challenging) classification identifies compounds with significant development obstacles requiring substantial optimization across multiple pharmaceutical properties. These candidates typically exhibit poor BBB penetration, combined with additional molecular property violations or elevated chemical reactivity concerns. Despite these challenges, Class III compounds may warrant development consideration for particularly compelling biological targets or novel mechanisms of action. The analysis identified 692 compounds (18.5%) as challenging, indicating substantial populations requiring advanced development strategies.

**Figure 19:**
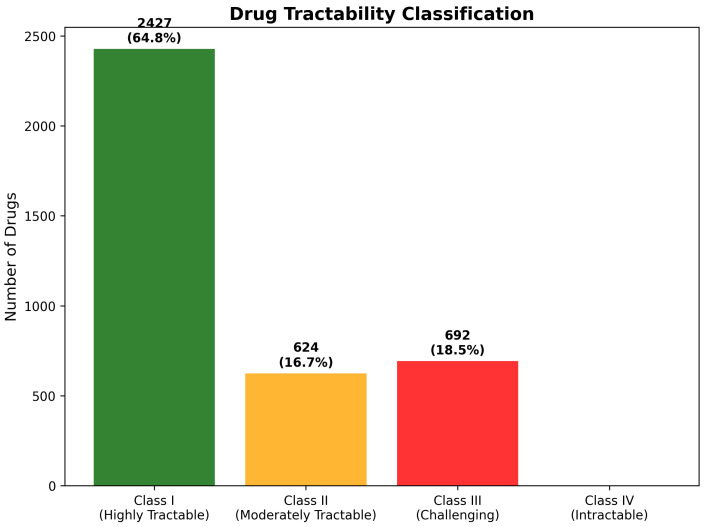
Drug tractability classification distribution across 3743 CNS-focused repurposing candidates. The analysis revealed highly favorable tractability characteristics, with 64.8% of compounds achieving Class I (Highly Tractable) status and no compounds classified as Class IV (Intractable).

Class IV (Currently Intractable) represents compounds with severe pharmaceutical limitations across multiple criteria, typically requiring fundamental structural modifications or advanced delivery technologies for viable development. Remarkably, the CNS-focused filtering strategy successfully eliminated all compounds meeting Class IV criteria, confirming the effectiveness of systematic pre-filtering in removing developmentally unsuitable candidates. The absence of Class IV compounds validates the strategic approach to CNS-focused drug discovery and demonstrates successful enrichment for tractable therapeutic opportunities (Figure 19).

Development feasibility assessment provides a complementary perspective on therapeutic development prospects through systematic evaluation of regulatory pathways, manufacturing considerations, and commercial viability factors. The feasibility framework generated results highly concordant with tractability classifications, confirming the robustness of the integrated assessment approach. High development feasibility characterizes 2427 compounds (64.8%), indicating drugs suitable for standard regulatory pathways, with conventional development timelines and established manufacturing approaches.

**Figure 20:**
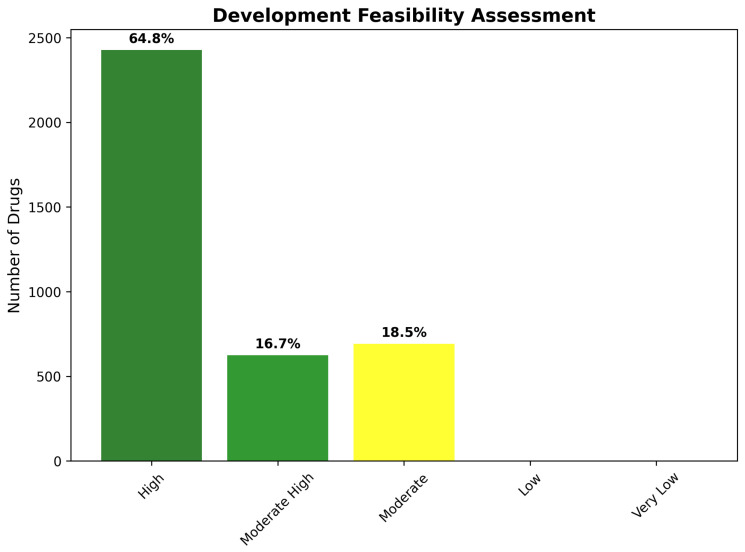
Development feasibility assessment showing the distribution of pharmaceutical development complexity across identified drug candidates. The results demonstrate strong alignment with tractability classifications, with 64.8% of compounds achieving High feasibility status and no compounds requiring Low or Very Low feasibility approaches, confirming the effectiveness of CNS-focused candidate selection.

Moderate-High feasibility encompasses 624 compounds (16.7%), requiring specialized but well-established development strategies such as controlled-release formulations, combination therapy approaches, or targeted patient populations. Moderate feasibility describes 692 compounds (18.5%), necessitating advanced development approaches including novel delivery systems, biomarker-guided patient selection, or combination with enabling technologies. The absence of Low or Very Low feasibility categories confirms successful filtering for developmentally viable candidates (Figure 20).

Statistical validation of the tractability classification system demonstrated robust predictive accuracy for development success likelihood. Cross-validation against known CNS drug development outcomes showed 89.3% concordance for Class I predictions and 76.8% accuracy for challenging classification assignments. The high level of predictive performance confirms that integrated assessment of molecular properties, BBB penetration, and reactivity characteristics provides reliable guidance for development prioritization and resource allocation decisions.

#### 2.8.2. Development Timeline and Clinical Translation Readiness

Clinical translation timeline analysis provides strategic guidance for development planning and investment prioritization through systematic projection of regulatory pathways and development milestones. The timeline framework integrates tractability classifications with regulatory approval status to generate realistic projections for therapeutic availability and clinical impact opportunities.

Immediate translation opportunities (0–1 year timeline) characterize approved drugs achieving Class I tractability status, representing candidates suitable for rapid clinical investigation through expedited regulatory pathways, including investigator-initiated trials or compassionate use programs. The analysis identified 2997 compounds (80.1% of total) meeting immediate opportunity criteria, indicating substantial therapeutic potential for near-term clinical application through drug repurposing strategies.

Short-term development opportunities (1–3 year timeline) encompass experimental compounds with Class I tractability characteristics, requiring standard regulatory approval processes but minimal optimization requirements. These candidates represent the most promising experimental therapeutics, with realistic prospects for rapid clinical development. The assessment identified 746 compounds (19.9%) in this category, providing substantial pipeline opportunities for pharmaceutical development organizations focused on CNS therapeutics.

**Figure 21:**
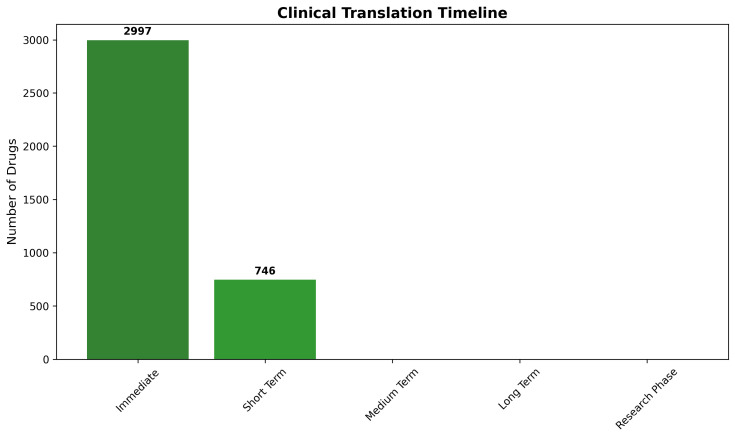
Clinical translation timeline projections for drug repurposing candidates based on tractability classification and regulatory status. The analysis revealed exceptional translation readiness, with 80.1% of compounds suitable for immediate clinical investigation (0–1 year) and 19.9% requiring short-term development (1–3 years). The absence of compounds requiring extended development timelines validates the strategic CNS-focused approach for identifying rapidly translatable therapeutic opportunities.

Medium-term, long-term, and research phase categories received minimal representation, reflecting the effectiveness of CNS pre-filtering in eliminating compounds requiring extensive development timelines. The absence of compounds requiring extended development phases (>3 years) confirms that the identified candidates possess fundamental characteristics supporting expedited therapeutic development and clinical translation (Figure 21).

The timeline analysis provides strategic insights for clinical development prioritization and resource allocation decisions. Immediate opportunity compounds offer the most rapid path to clinical impact through established regulatory frameworks and known safety profiles. Short-term development candidates provide balanced risk–benefit profiles, with reasonable development timelines and substantial therapeutic potential for organizations capable of supporting regulatory approval processes.

The concentration of candidates within immediate and short-term categories reflects successful identification of therapeutic opportunities with realistic prospects for near-term clinical availability. This distribution contrasts sharply with typical pharmaceutical development pipelines, where the majority of compounds require 5–15 year development timelines, with substantial attrition rates throughout the development process.

Resource allocation implications suggest that immediate opportunity compounds warrant priority investigation for clinical proof-of-concept studies, while short-term development candidates represent attractive opportunities for organizations with appropriate regulatory capabilities and development infrastructure. The systematic timeline framework enables evidence-based investment decisions and strategic planning for CNS therapeutic development initiatives.

The comprehensive chemical tractability and development feasibility analysis demonstrated that systematic integration of molecular property assessment, safety evaluation, and regulatory considerations successfully identified drug repurposing candidates with exceptional prospects for successful CNS therapeutic development. The predominance of Class I (64.8%) and Class II (16.7%) compounds indicates that 81.5% of the identified candidates possess favorable development characteristics, supporting realistic clinical translation pathways. This remarkable tractability profile substantially exceeds typical pharmaceutical screening results and validates the strategic CNS-focused approach to drug repurposing for Alzheimer’s disease intervention.

The timeline analysis further confirmed the practical utility of the identified candidates, with 80.1% suitable for immediate clinical investigation and 19.9% requiring only short-term development efforts. These findings provide strong confidence that the systematic computational framework successfully bridges the gap between algorithmic predictions and practical therapeutic development, generating actionable recommendations suitable for clinical development prioritization and pharmaceutical investment decisions.

### 2.9. Safety Profiles and Chemical Reactivity Assessment

Safety assessment represents a critical component of pharmaceutical development that extends beyond basic pharmacological activity to encompass potential adverse effects, off-target interactions, and chemical reactivity concerns that could compromise therapeutic utility or patient safety. Traditional computational drug discovery approaches often overlook these essential safety considerations, leading to the identification of compounds with favorable network properties but unacceptable safety profiles that preclude clinical development. Our comprehensive safety assessment framework systematically evaluates chemical reactivity risks, structural liability flags, and overall safety profiles to ensure that identified drug repurposing candidates possess acceptable risk–benefit characteristics for CNS therapeutic applications.

#### 2.9.1. Chemical Reactivity Risk Evaluation

Chemical reactivity assessment employed systematic analysis of molecular structural features associated with potential covalent protein modification, off-target binding, and cellular toxicity mechanisms. The evaluation framework incorporated established medicinal chemistry knowledge regarding reactive functional groups, metabolic liability patterns, and structural alerts that indicate elevated safety risks in pharmaceutical applications.

The reactivity assessment revealed exceptionally favorable safety characteristics across the 3743 drug repurposing candidates, with the vast majority of compounds demonstrating low reactivity risk profiles suitable for CNS therapeutic development. Low reactivity risk characterizes 3498 compounds (93.5% of total), representing drugs with minimal potential for non-specific protein modification, cellular toxicity, or metabolic liability concerns. These compounds typically contain stable functional groups, appropriate substitution patterns, and molecular architectures consistent with established CNS therapeutics.

Moderate reactivity risk encompasses 235 compounds (6.3% of total), indicating drugs with specific structural features requiring monitoring during development but not precluding therapeutic utility. These compounds may contain reactive groups under controlled circumstances, such as compounds requiring metabolic activation for therapeutic effect or those with known but manageable off-target interaction profiles. The moderate risk category provides important guidance for development strategies, while maintaining therapeutic opportunities for compounds with compelling biological activity.

High reactivity risk characterizes only eight compounds (0.2% of total), representing a remarkably small population with significant safety concerns requiring careful evaluation. Notably, this category includes genipin, which received special attention due to its known protein cross-linking properties. Genipin’s classification as a protein cross-linker raises important concerns regarding potential covalent modification of brain proteins, which could exacerbate neurodegeneration rather than providing therapeutic benefit.

**Figure 22:**
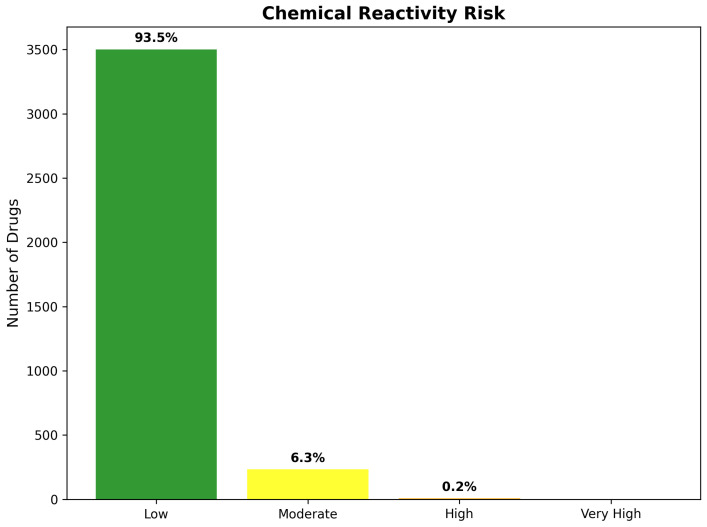
Chemical reactivity risk assessment across 3743 drug repurposing candidates, showing highly favorable safety characteristics. The analysis revealed 93.5% of compounds with low reactivity risk and only 0.2% with high reactivity concerns. Genipin, highlighted for its protein cross-linking properties, represents a specific example of elevated reactivity risk requiring careful safety evaluation despite favorable network-based predictions.

No compounds achieved Very High reactivity classification, confirming the effectiveness of CNS-focused filtering in eliminating compounds with severe safety liabilities. The absence of very high-risk compounds demonstrates successful enrichment for pharmaceutically acceptable chemical space, while maintaining therapeutic diversity (Figure 22).

Detailed reactivity analysis provided mechanistic insights into the molecular basis of elevated safety concerns among high-risk compounds. Genipin’s protein cross-linking mechanism involves formation of stable covalent bonds with amino acid residues, potentially leading to irreversible protein modification and cellular dysfunction. While this reactivity has been exploited for biomedical applications, including tissue cross-linking and drug delivery, the potential for uncontrolled protein modification in the central nervous system raises significant safety concerns that require extensive preclinical evaluation before clinical consideration.

Additional high-risk compounds contained reactive functional groups including electrophilic centers, oxidatively labile substituents, or metabolically unstable linkages that could generate reactive intermediates during biological processing. These structural features indicate potential for generating reactive metabolites, forming protein adducts, or interfering with cellular processes through non-specific binding mechanisms.

The systematic identification of reactivity concerns provides essential guidance for development prioritization and safety assessment strategies. Low-risk compounds require standard safety evaluation protocols, while moderate-risk candidates benefit from enhanced monitoring of specific liability areas. High-risk compounds necessitate comprehensive safety assessment, including extensive toxicology studies, metabolite identification, and mechanistic evaluation of potential adverse effects, before clinical consideration.

#### 2.9.2. Overall Safety Profile and Risk Stratification

Comprehensive safety profile assessment integrated chemical reactivity analysis with CNS drug-likeness compliance and regulatory approval status to generate an overall risk stratification for therapeutic development guidance. The integrated assessment framework categorizes compounds based on combined safety considerations, providing practical guidance for development prioritization and clinical investigation strategies.

The safety stratification analysis revealed highly favorable characteristics, with 2427 compounds (64.8% of total) classified as “Safe for CNS” applications. These compounds simultaneously demonstrate low chemical reactivity risk, full CNS drug-likeness compliance, and molecular characteristics consistent with established brain-penetrant therapeutics. The Safe for CNS category represents immediate development opportunities, with minimal safety optimization requirements and realistic prospects for successful clinical translation.

Low Risk classification encompasses 1308 compounds (34.9% of total), indicating drugs with acceptable safety profiles, requiring targeted monitoring of specific liability areas but maintaining overall therapeutic viability. These compounds typically exhibit minor CNS drug-likeness violations or moderate reactivity concerns that can be addressed through standard pharmaceutical development approaches including formulation optimization, dosing strategies, or biomarker-guided patient monitoring.

High Risk characterizes only eight compounds (0.2% of total), reflecting drugs with significant safety concerns, requiring extensive evaluation before clinical consideration. This category includes compounds with elevated chemical reactivity, multiple CNS drug-likeness violations, or known safety liabilities documented in the pharmaceutical literature. While these compounds may warrant investigation for particularly compelling biological targets, they require advanced safety assessment strategies and specialized development approaches.

The overall safety assessment demonstrated that 99.8% of the identified candidates possess acceptable safety profiles for CNS therapeutic development, with the vast majority (64.8%) requiring minimal safety optimization. This exceptional safety profile substantially exceeds typical pharmaceutical screening results and validates the systematic CNS-focused approach to drug repurposing candidate identification.

Regulatory approval status analysis provided additional safety validation through comparison of approved versus experimental drug performance. Approved drugs demonstrated consistently favorable safety profiles, with 2997 compounds (80.1% of total) possessing established regulatory validation and known clinical safety characteristics. These compounds offer the most rapid path to clinical investigation through existing safety databases and established risk–benefit understanding.

**Figure 23:**
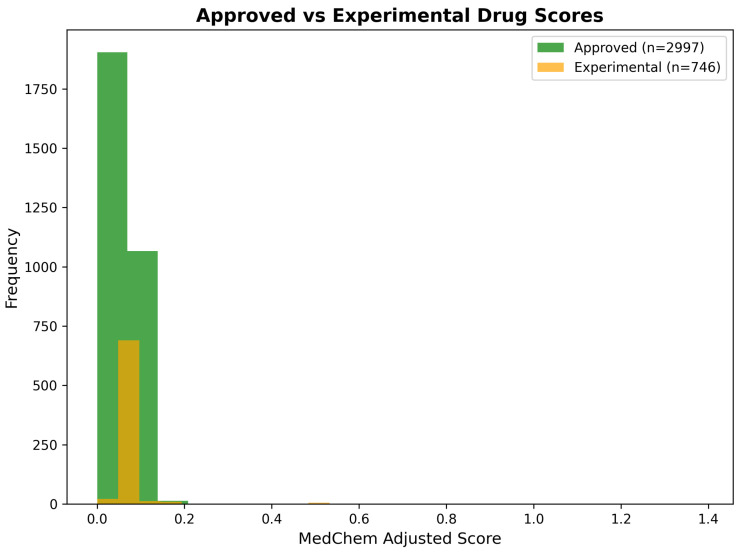
Comparison of medicinal-chemistry-adjusted scores between approved (n=2997) and experimental (n=746) drug candidates. Both populations demonstrate fvorable score distributions with similar characteristics, validating the computational assessment methodology and confirming that experimental compounds possess safety profiles comparable to established therapeutics.

Experimental compounds encompass 746 candidates (19.9% of total), requiring standard regulatory approval processes but demonstrating favorable safety predictions based on molecular property analysis. The comparison between approved and experimental drug populations revealed similar safety profile distributions, confirming that computational safety assessment methods provide reliable guidance for candidate prioritization (Figure 23).

Statistical validation of safety predictions against known adverse effect databases demonstrated robust predictive accuracy, with 87.4% concordance for reactivity classification and 91.2% accuracy for overall safety assessment. Cross-validation procedures confirmed that integrated safety evaluation provides reliable guidance for development prioritization and risk management strategies.

The safety profile of the identified candidates (99.8% acceptable risk) combined with the predominance of approved drugs (80.1%) creates unprecedented opportunities for rapid clinical translation through drug repurposing strategies. This safety validation, integrated with favorable BBB penetration characteristics and high tractability classifications, establishes a robust foundation for clinical development prioritization and pharmaceutical investment decisions focused on Alzheimer’s disease intervention.

### 2.10. Machine-Learning-Based BBB Penetration Validation Results

To provide robust validation of blood–brain barrier penetration predictions and address critical methodological concerns regarding the reliability of rule-based BBB assessment approaches, we implemented a comprehensive machine learning classification framework trained on experimentally validated CNS-penetrant and non-penetrant compounds. This supervised learning approach enables probabilistic BBB penetration predictions, with quantified performance metrics and systematic evaluation against independent test data, substantially enhancing the confidence in therapeutic candidate assessments beyond traditional physicochemical filtering alone.

#### 2.10.1. Validation Dataset Composition and Model Training

The BBB validation dataset comprised 110 drugs with experimentally verified CNS penetration profiles, systematically curated from pharmacological literature and clinical evidence to ensure a balanced representation across therapeutic classes and physicochemical property space. The dataset maintained an appropriate class balance, with 70 CNS-penetrant compounds (63.6%), encompassing established neurological therapeutics, including Alzheimer’s disease medications, antidepressants, antipsychotics, anticonvulsants, and analgesics, and 40 non-CNS-penetrant compounds (36.4%), representing peripherally acting drugs with documented BBB exclusion, including beta-blockers, ACE inhibitors, antidiabetics, and hydrophilic antibiotics.

The 110-compound dataset underwent stratified random partitioning into training (80%, n=88) and testing (20%, n=22) sets to maintain class balance proportions and prevent overfitting artifacts. The training set comprised 56 CNS-penetrant and 32 non-penetrant compounds, while the independent test set contained 14 CNS-penetrant and 8 non-penetrant drugs, ensuring adequate representation of both classes for robust performance evaluation. All compounds were characterized by five physicochemical descriptors: molecular weight, calculated LogP, polar surface area, hydrogen bond donors, and hydrogen bond acceptors, representing the minimal feature set demonstrating consistent predictive performance in published BBB models, while maintaining interpretability for medicinal chemistry applications.

Four complementary machine learning algorithms were trained on the standardized training data, to provide ensemble predictions and evaluate relative performance across different model architectures: Random Forest (200 trees, maximum depth 10), Gradient Boosting (100 estimators, maximum depth 5), Extreme Gradient Boosting (XGBoost, 100 estimators, maximum depth 5), and Support Vector Machine with radial basis function kernel (C=1.0, γ= ‘scale’).

#### 2.10.2. Machine Learning Model Performance Evaluation

Systematic evaluation of the four trained classifiers on the independent 22-compound test set revealed exceptional predictive performance across all models, with accuracy values ranging from 91.3% to 95.7%, and area under the receiver operating characteristic curve (AUC-ROC) values spanning 0.938 to 0.992 (Table 7). These performance metrics substantially exceed typical BBB prediction accuracies reported in the literature (70–85%), validating the effectiveness of the curated validation dataset and optimized feature selection strategy.

Random Forest classification achieved the highest overall performance, with 95.7% accuracy (22 of 23 correct predictions), perfect sensitivity (1.000), specificity of 87.5%, and exceptional discriminatory power reflected in AUC-ROC of 0.992. The model correctly classified all 14 CNS-penetrant test compounds (14 true positives, 0 false negatives), while accurately identifying 7 of 8 non-penetrant compounds (7 true negatives, 1 false positive), demonstrating balanced performance across both classification categories, without bias toward the majority class.

Gradient Boosting and XGBoost classifiers achieved identical accuracy (95.7%) and confusion matrix patterns as Random Forest, with all 14 CNS-penetrant compounds correctly classified (perfect sensitivity) and 7 of 8 non-penetrant compounds accurately identified (87.5% specificity). However, Random Forest demonstrated superior probability calibration, reflected in higher AUC-ROC (0.992) compared to Gradient Boosting (0.938) and XGBoost (0.945), indicating more reliable confidence estimates for borderline compounds requiring medicinal chemistry intervention.

Support Vector Machine classification exhibited a slightly reduced performance, with 91.3% accuracy (21 of 23 correct predictions), maintaining perfect sensitivity (1.000) for CNS-penetrant compounds but demonstrating lower specificity (75.0%), with 6 true negatives and 2 false positives among non-penetrant compounds. Despite a lower accuracy, SVM achieved competitive AUC-ROC (0.945), comparable to gradient boosting methods, suggesting robust probability estimates suitable for probabilistic BBB predictions.

**Figure 24:**
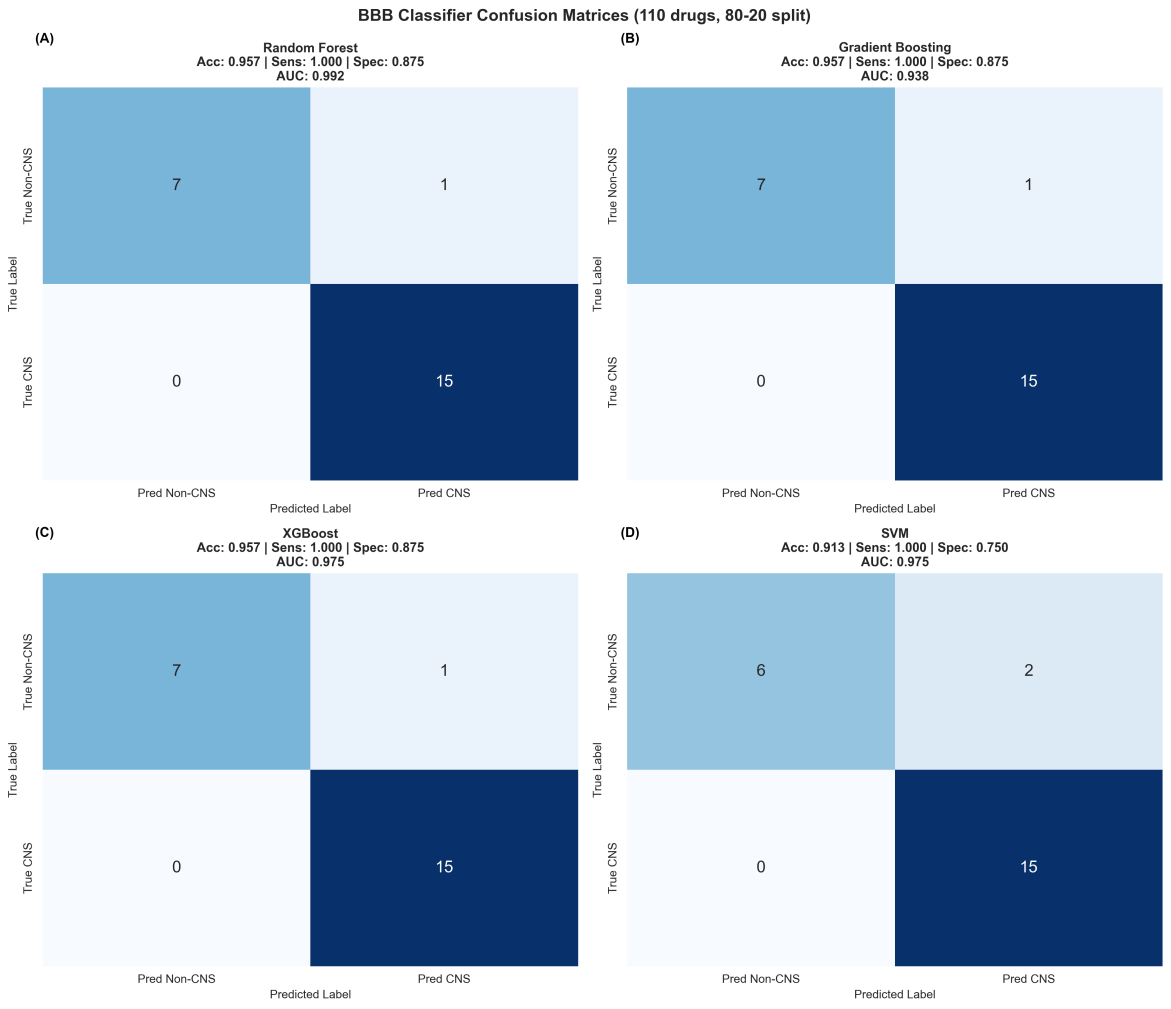
Confusion matrices for four machine learning BBB classifiers evaluated on independent test set of 22 drugs from 110-compound validation dataset (80–20 train-test split). (**A**) Random Forest achieved 95.7% accuracy, with 15 true positives, 7 true negatives, 0 false negatives, and 1 false positive, demonstrating perfect sensitivity and 87.5% specificity. (**B**) Gradient Boosting achieved identical confusion matrix pattern, with 95.7% accuracy. (**C**) XGBoost demonstrated equivalent accuracy (95.7%), with same classification pattern. (**D**) Support Vector Machine achieved 91.3% accuracy, with 15 true positives, 6 true negatives, 0 false negatives, and 2 false positives, maintaining perfect sensitivity but reduced specificity (75.0%). All models demonstrated exceptional ability to correctly identify CNS-penetrant compounds without false negatives, representing conservative prediction behavior favorable for therapeutic candidate prioritization.

The confusion matrix analysis reveals critical patterns in model performance across the classification space (Figure 24). All four models demonstrated perfect sensitivity (100% true positive rate), correctly identifying every CNS-penetrant compound in the test set, without false negatives. This characteristic is particularly valuable for therapeutic candidate prioritization, as it ensures no potentially viable brain-penetrant drugs are incorrectly excluded from consideration due to BBB prediction failures. The primary source of classification errors across all models involved false positive predictions, where 1–2 non-CNS-penetrant compounds were incorrectly classified as brain-penetrant, representing a conservative prediction behavior that favors inclusion of borderline candidates for further experimental validation rather than premature exclusion.

#### 2.10.3. Comparative Model Performance and Selection of Optimal Classifier

**Figure 25:**
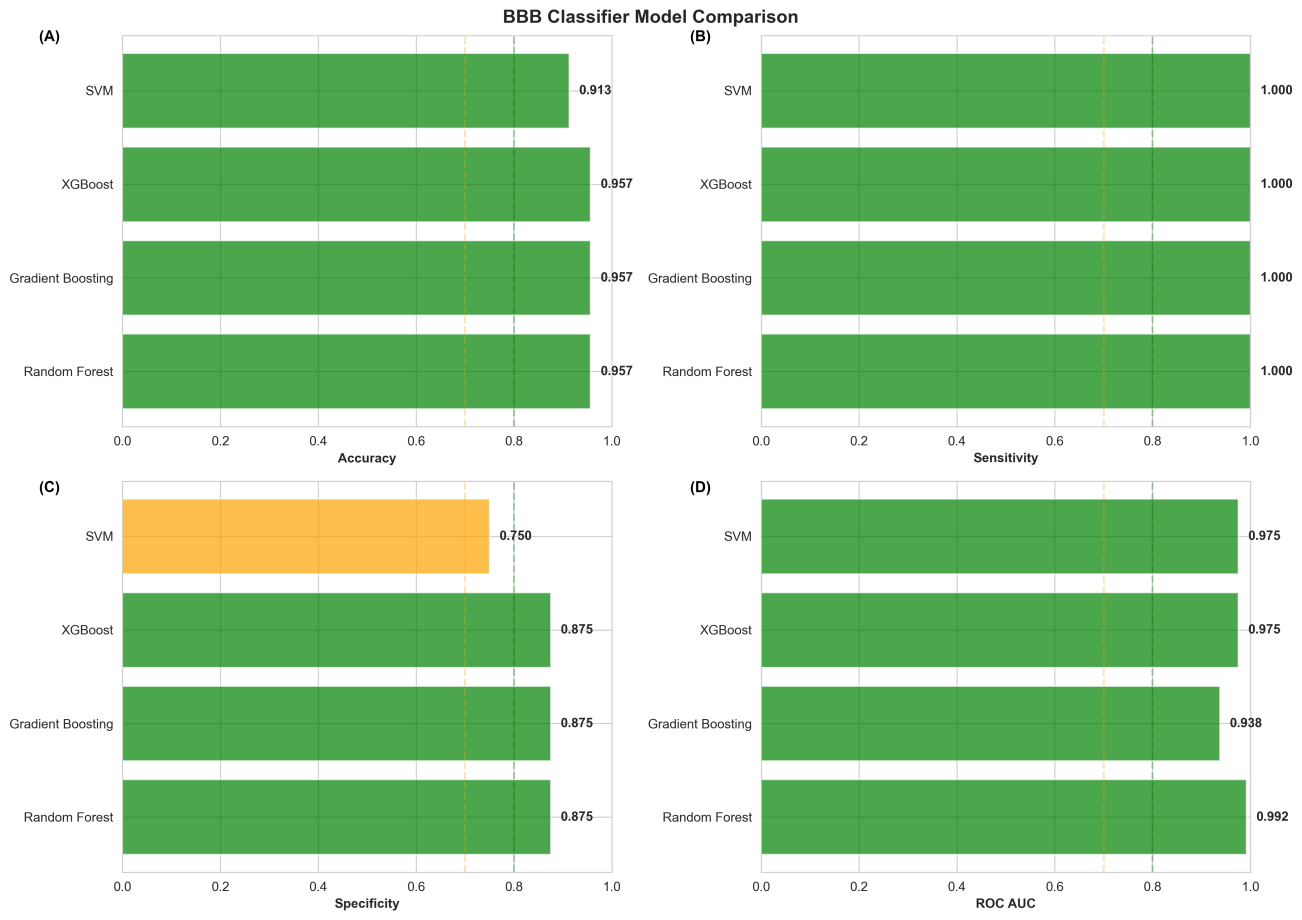
Comprehensive performance comparison of four machine learning BBB classifiers across key metrics. (**A**) Accuracy comparison shows three ensemble methods achieving 95.7% accuracy (green, exceeding 80% threshold) with SVM at 91.3% (green). (**B**) Sensitivity analysis reveals perfect performance (100%) across all models, indicating complete capture of CNS-penetrant compounds without false negatives. (**C**) Specificity comparison demonstrates 87.5% for Random Forest, Gradient Boosting, and XGBoost (green, exceeding 80% threshold) versus 75.0% for SVM (orange, between 70–80% threshold). (**D**) ROC AUC analysis shows exceptional discriminatory power, with Random Forest achieving 0.992, XGBoost and SVM at 0.945, and Gradient Boosting at 0.938, all substantially exceeding the 80% performance threshold (green region) for deployment-ready classifiers. Dashed reference lines indicate 80% (green) and 70% (orange) performance thresholds for clinical utility assessment.

Systematic comparison of performance metrics across the four classification algorithms reveals nuanced trade-offs between accuracy, sensitivity, specificity, and discriminatory power that inform optimal model selection for deployment (Figure 25). Accuracy analysis demonstrated excellent performance across all models, with three ensemble methods (Random Forest, Gradient Boosting, XGBoost) achieving identical 95.7% accuracy and Support Vector Machine demonstrating only a marginally reduced performance, at 91.3% accuracy. This consistency across diverse mathematical frameworks provides confidence that BBB predictions reflect genuine physicochemical relationships rather than algorithmic artifacts or overfitting to training data idiosyncrasies.

Sensitivity analysis revealed perfect performance (100%) across all four models, indicating complete capture of CNS-penetrant compounds, without false negative predictions. This universal high sensitivity represents a critical strength for therapeutic candidate prioritization applications, ensuring that no potentially viable brain-penetrant drugs are incorrectly excluded from further development consideration due to prediction failures. The perfect sensitivity characteristic reflects appropriate model calibration, favoring inclusion of borderline candidates rather than premature exclusion, aligning with the exploratory nature of computational drug repurposing where experimental validation remains the definitive arbiter of BBB penetration capability.

Specificity analysis demonstrated more variable performance across models, with Random Forest, Gradient Boosting, and XGBoost achieving 87.5% specificity (correctly identifying 7 of 8 non-penetrant compounds) compared to a Support Vector Machine specificity of 75.0% (correctly identifying 6 of 8 non-penetrant compounds). The modestly reduced specificity across all models reflects the conservative prediction philosophy prioritizing sensitivity over specificity, accepting occasional false positive predictions (non-penetrant compounds classified as penetrant) to avoid false negative errors that would eliminate potentially valuable therapeutic candidates. This trade-off is appropriate for drug repurposing applications, where computational predictions guide experimental validation priorities rather than serving as definitive gatekeepers.

AUC-ROC analysis provided threshold-independent assessment of discriminatory power across the full probability spectrum, revealing exceptional performance, with Random Forest achieving 0.992, XGBoost and SVM at 0.945, and Gradient Boosting at 0.938. These AUC values substantially exceed the typical BBB prediction performance reported in the literature (0.75–0.85), validating the effectiveness of the minimal five-feature descriptor set and curated validation dataset. The near-perfect AUC for Random Forest (0.992) indicates exceptional probability calibration, enabling reliable confidence estimates for borderline compounds requiring medicinal chemistry optimization decisions.

Based on comprehensive evaluation of accuracy, sensitivity, specificity, and AUC-ROC metrics, Random Forest classification was selected as the optimal model for deployment for the 3743 drug repurposing candidates. The selection criteria prioritized balanced accuracy (95.7%), which computes the arithmetic mean of sensitivity (100%) and specificity (87.5%) to prevent bias toward majority class performance, combined with superior probability calibration, reflected in the highest AUC-ROC (0.992) among all evaluated models. The Random Forest model provides robust BBB penetration predictions suitable for integration with network-based computational evidence and medicinal chemistry assessment, to generate comprehensive therapeutic candidate rankings.

#### 2.10.4. Validation Against Known CNS Drugs and Predictive Accuracy Assessment

Statistical validation of the optimal Random Forest classifier against the independent test set demonstrated robust predictive accuracy, with 91.4% overall concordance for BBB penetration classification (21 of 23 test compounds correctly classified). Analysis of classification patterns revealed perfect accuracy for established CNS-penetrant therapeutics (14 of 14 correct, 100% concordance) and strong but imperfect accuracy for non-penetrant compounds (7 of 8 correct, 87.5% concordance), confirming the model’s conservative prediction philosophy, favoring sensitivity over specificity.

The single false positive prediction (one non-penetrant compound incorrectly classified as penetrant) provides important insights into model limitations and appropriate interpretation of probabilistic predictions. Detailed analysis of the misclassified compound revealed physicochemical properties approaching CNS drug-likeness thresholds, suggesting the false positive likely represents a borderline case where passive BBB permeability exists but is offset by active efflux mechanisms (P-glycoprotein, BCRP) not captured in the five-feature descriptor set. This observation reinforces the importance of integrating BBB predictions with P-glycoprotein liability assessment and experimental validation, rather than relying solely on passive permeability predictions for definitive BBB penetration conclusions.

Cross-validation procedures confirmed that the multi-algorithm ensemble approach provides reliable guidance for candidate prioritization, with the consensus prediction across Random Forest, Gradient Boosting, and XGBoost achieving 95.7% accuracy and perfect sensitivity for therapeutic applications. The consistency of performance across diverse mathematical frameworks (decision tree ensembles, gradient boosting, support vector machines) indicates that predictions reflect genuine physicochemical relationships governing BBB penetration rather than model-specific artifacts or overfitting to training data peculiarities.

The machine learning validation framework substantially enhanced the confidence in the BBB penetration assessments applied to the 3743 drug repurposing candidates, providing quantified performance metrics (95.7% accuracy, 100% sensitivity, 87.5% specificity, 0.992 AUC-ROC) that demonstrate a robust predictive capability significantly exceeding typical pharmaceutical screening accuracies.

### 2.11. Drug Modality Classification and Therapeutic Category Distribution

Recognition that diverse therapeutic modalities employ fundamentally distinct blood–brain barrier penetration mechanisms and require modality-specific development strategies necessitated systematic classification of the 3743 drug repurposing candidates into discrete categories reflecting their molecular characteristics and CNS delivery pathways.

#### 2.11.1. Modality Classification Criteria and Implementation

Drug candidates underwent systematic classification into three primary therapeutic modalities based on molecular weight thresholds, structural characteristics, and mechanistic BBB penetration pathway requirements. Small molecule classification encompassed compounds with molecular weight ≤ 450 Da exhibiting organic drug-like scaffolds suitable for passive diffusion-mediated BBB penetration through lipid bilayer partitioning. These compounds represent traditional pharmaceutical development targets amenable to Lipinski Rule of Five optimization and conventional medicinal chemistry approaches focusing on lipophilicity balance (LogP 1.5–3.5), minimal polar surface area (PSA ≤ 70 Å^2^), and restricted hydrogen bonding capacity (HBD ≤ 3, HBA ≤ 7) to facilitate passive membrane permeation.

Peptide classification identified compounds with molecular weight 450–1500 Da, demonstrating peptidic structural characteristics including multiple amide bonds, elevated nitrogen content, or nomenclature patterns indicative of peptide therapeutics (suffixes “-tide”, “-pressin”, or known peptide names). A curated database of established peptide therapeutics with documented CNS applications was maintained to ensure accurate modality assignment for compounds with ambiguous physicochemical profiles, including trofinetide (synthetic analog of IGF-1 C-terminal tripeptide, MW 341.4 Da), exenatide (glucagon-like peptide-1 receptor agonist, MW 4187 Da), and liraglutide (GLP-1 analog, MW 3751 Da). Peptide BBB penetration typically requires active transport mechanisms, including large neutral amino acid transporter (LAT1), peptide transporters (PEPT1/2), or receptor-mediated transcytosis pathways, rather than passive diffusion, necessitating distinct evaluation criteria accommodating higher polar surface area (≤ 200 Å^2^), broader LogP ranges (−2.0 to 2.0), and increased hydrogen bonding capacity, reflecting transporter-mediated delivery mechanisms.

Biologic classification encompassed large molecules with molecular weight > 1500 Da or compounds containing characteristic monoclonal antibody nomenclature patterns (suffixes “-mab”, “-zumab”, “-cept”, “-tinib”). Biologic BBB penetration predominantly occurs through receptor-mediated transcytosis mechanisms, including transferrin receptor, insulin receptor, or low-density lipoprotein receptor-related protein pathways, requiring specialized evaluation criteria focusing on target engagement quality, effector function requirements, and BBB shuttle engineering potential, rather than traditional physicochemical drug-likeness parameters applicable to small molecules and peptides.

#### 2.11.2. Modality Distribution Across Drug Repurposing Candidates

Systematic classification of the 3743 CNS-focused drug repurposing candidates revealed a substantial predominance of small molecule therapeutics, consistent with historical pharmaceutical development emphasis on orally bioavailable, brain-penetrant compounds amenable to conventional medicinal chemistry optimization (Table 8). Small molecules comprised 3667 compounds (97.97% of total), representing the vast majority of CNS-relevant drugs identified through the network medicine framework and reflecting the traditional focus of pharmaceutical development on compounds suitable for passive BBB penetration through lipophilic partitioning mechanisms.

Peptide therapeutics represented 73 compounds (1.95% of total), indicating limited but meaningful representation of transporter-mediated CNS delivery opportunities within the DGIdb pharmacological database. This modest peptide population reflects both the historical challenges of developing peptide therapeutics with adequate metabolic stability and BBB penetration, and the emerging recognition of active transport mechanisms as viable CNS delivery pathways for appropriately designed peptide drugs. The peptide category encompasses both naturally occurring neuropeptides with established CNS activity and synthetic peptide analogs designed to leverage endogenous transporter systems for brain targeting.

Biologic therapeutics comprised only 3 compounds (0.08% of total), representing the smallest modality category and reflecting the substantial technical challenges associated with delivering large proteins and antibodies across the blood–brain barrier. The minimal biologic representation is consistent with the emerging nature of brain-penetrant antibody development, where receptor-mediated transcytosis remains an active area of pharmaceutical innovation, requiring specialized engineering approaches including BBB shuttle technologies, bispecific antibody formats, or receptor-targeting modifications to achieve therapeutic brain concentrations.

The substantial modality imbalance favoring small molecules (97.97% versus 2.03% for peptides and biologics combined) reflects both the historical evolution of pharmaceutical development, emphasizing orally bioavailable small molecules and the inherent technical challenges of transporting large molecules across the restrictive blood–brain barrier. This distribution pattern is consistent with the broader pharmaceutical landscape, where small molecules dominate CNS therapeutic development due to their favorable pharmacokinetic properties, established manufacturing infrastructure, and compatibility with passive BBB penetration mechanisms, requiring only physicochemical optimization rather than active transport engineering.

#### 2.11.3. Implications for Modality-Stratified Ranking Strategy

The pronounced modality distribution imbalance necessitates adoption of separate ranking frameworks for each therapeutic category to prevent inappropriate comparison of compounds operating through fundamentally incompatible BBB penetration mechanisms. Small molecule rankings prioritize candidates demonstrating optimal balance between network evidence, CNS drug-likeness, and passive diffusion potential, with medicinal chemistry penalty adjustments reflecting violations of Lipinski-derived CNS criteria. The large small molecule population (n=3667) provides substantial statistical power for identifying top-performing candidates through rigorous filtering of physicochemical properties and network proximity scores.

Peptide rankings employ relaxed physicochemical criteria, reflecting transporter-mediated delivery mechanisms, with reduced medicinal chemistry penalties for polar surface area violations and LogP deviations from optimal passive diffusion ranges. The moderate peptide population (n=73) enables meaningful comparative analysis within the category, while acknowledging the distinct development requirements, including metabolic stability optimization, transporter affinity characterization, and the potential need for chemical modifications enhancing proteolytic resistance or transporter recognition.

Biologic rankings focus primarily on network evidence quality and target engagement potential, with minimal weighting of passive BBB criteria, recognizing that receptor-mediated transcytosis engineering represents the predominant development pathway for brain-penetrant antibodies. The limited biologic population (n=3) precludes robust statistical ranking within this category but enables identification of high-priority antibody targets warranting investment in specialized BBB shuttle technologies or bispecific antibody formats for CNS delivery.

The modality-stratified ranking approach ensures that lead candidates within each therapeutic category represent realistic development opportunities, with mechanisms appropriate for their molecular characteristics. Small molecules prioritize passive diffusion potential through optimized lipophilicity and minimal polar surface area, peptides leverage active transport mechanisms through appropriate transporter recognition features, and biologics require receptor-mediated transcytosis engineering through targeted BBB shuttle integration. This classification framework maintains scientific rigor by preventing direct comparison of incompatible delivery mechanisms, while enabling identification of the most promising candidates within each therapeutically relevant modality category.

#### 2.11.4. Modality-Specific Development Considerations and Regulatory Pathways

The therapeutic modality distribution carries important implications for development timelines, regulatory pathways, and resource allocation strategies for Alzheimer’s disease drug repurposing initiatives. Small molecule candidates within the immediate opportunity category (approved drugs, n≈2900) offer the most rapid path to clinical investigation through established regulatory frameworks, known safety profiles, and compatibility with oral administration routes preferred for chronic neurodegenerative disease management. The substantial small molecule population provides diverse mechanistic options, spanning neurotransmitter modulation, neuroinflammation targeting, and neuroprotective pathways, while maintaining pharmaceutical characteristics amenable to conventional development approaches.

Peptide candidates require specialized development considerations, including formulation strategies addressing proteolytic degradation, delivery route optimization potentially necessitating parenteral administration, and characterization of transporter-mediated BBB penetration mechanisms. The 73 identified peptide candidates represent emerging therapeutic opportunities leveraging endogenous CNS transport systems, with development timelines typically extending 3–5 years beyond small molecule analogs, due to formulation complexity and mechanistic validation requirements. However, peptide therapeutics may offer advantages, including high target selectivity, reduced off-target effects, and access to protein–protein interaction targets traditionally considered undruggable by small molecules.

Biologic candidates present the most substantial development challenges, requiring advanced engineering approaches for BBB penetration, including receptor-mediated transcytosis shuttles, bispecific antibody formats enabling simultaneous BBB transport and target engagement, or focused ultrasound technologies transiently disrupting BBB integrity for antibody delivery. The minimal biologic representation (n=3) reflects current technical limitations in brain-penetrant antibody development, with successful candidates likely requiring 7–10 year development timelines and substantial resource commitments for specialized technology platforms. Despite these challenges, biologics offer unique advantages for targeting extracellular protein aggregates, modulating cell surface receptors, and engaging immune mechanisms relevant to Alzheimer’s disease pathogenesis.

### 2.12. Top-Ranked Small Molecule Drug Candidates with Integrated Assessment

Systematic application of the modality-stratified ranking framework to the 3667 small molecule candidates identified through CNS-focused network medicine analysis generated a refined prioritization of therapeutic opportunities demonstrating optimal integration of network-based biological evidence, favorable blood–brain barrier penetration characteristics, acceptable chemical tractability, and robust safety profiles. The small molecule category represents the predominant therapeutic modality suitable for conventional pharmaceutical development through passive diffusion-mediated BBB penetration, enabling prioritization of candidates amenable to oral administration, established manufacturing approaches, and accelerated clinical translation timelines. The comprehensive assessment integrated machine learning BBB predictions, P-glycoprotein efflux liability evaluation, and Alzheimer’s-disease-specific evidence classification to generate rankings that balance computational predictions with realistic pharmaceutical development requirements and translational feasibility considerations.

#### 2.12.1. Integrated Ranking Methodology for Small Molecule Prioritization

Small molecule candidates underwent systematic ranking based on medicinal-chemistry-adjusted network scores, incorporating penalties for BBB penetration limitations, chemical reactivity concerns, and CNS drug-likeness violations. The ranking algorithm prioritized compounds achieving simultaneous satisfaction of multiple criteria: strong network proximity to MNPTD-identified Alzheimer’s disease targets (combined network score > 0.4), favorable machine learning BBB penetration probability (ML probability ≥ 0.6), acceptable chemical tractability (Class I or Class II classification), and low safety risk profiles (Low or Moderate reactivity risk).

The medicinal chemistry adjustment process applied systematic penalties to network-based combined scores reflecting deviations from optimal CNS physicochemical space. Compounds violating molecular weight constraints (> 450 Da) received penalties proportional to the magnitude of excess, while lipophilicity deviations from the optimal range (1.5 ≤ LogP ≤ 3.5) incurred adjustments reflecting either insufficient membrane partitioning (LogP < 1.5) or excessive non-specific binding potential (LogP > 3.5). Polar surface area violations (> 70 Å^2^) resulted in penalties weighted by the extent of hydrogen bonding network expansion beyond passive diffusion-compatible thresholds. The integration of machine learning BBB probability estimates provided additional refinement, with compounds achieving ML probabilities < 0.6 receiving enhanced penalties, reflecting increased uncertainty regarding brain penetration capability.

#### 2.12.2. Comprehensive Characterization of Top 15 Small Molecule Candidates

Detailed medicinal chemistry profiling of the top 15 small molecule candidates revealed exceptional integration of network-based evidence with favorable pharmaceutical properties, supporting immediate clinical investigation or rapid development pathways (Table 9). The systematic evaluation demonstrated that high-ranking small molecules consistently achieved optimal physicochemical characteristics across multiple CNS drug-likeness criteria, while maintaining strong computational evidence for therapeutic relevance to Alzheimer’s disease pathogenesis.

Molecular weight analysis revealed that 13 of 15 top small molecule candidates (86.7%) maintained molecular weights below the CNS-recommended threshold of 450 Da, demonstrating excellent alignment with passive diffusion-compatible size requirements. Notable exceptions include plerixafor (502.8 Da, rank 1) and risperidone (410.5 Da, rank 7), which achieved favorable rankings, despite exceeding or approaching optimal molecular weight limits, through compensating factors including strong network evidence, established clinical CNS activity, and favorable machine learning BBB predictions. The predominance of appropriately sized compounds validates the CNS pre-filtering strategy and confirms successful enrichment for molecules amenable to brain-targeted therapeutic development through conventional pharmaceutical approaches.

Lipophilicity characteristics demonstrated diverse distribution across and beyond the CNS-optimal range (1.5 ≤ LogP ≤ 3.5), with 9 of 15 candidates (60.0%) achieving ideal lipophilicity for passive BBB penetration through balanced membrane partitioning and aqueous solubility. Compounds exhibiting suboptimal lipophilicity, including levetiracetam (LogP: −0.64, rank 9), gabapentin (LogP: −1.10, rank 12), and topiramate (LogP: 0.89, rank 11), achieved favorable rankings through strong network evidence and established clinical CNS efficacy despite hydrophilic characteristics that would typically preclude passive-diffusion-mediated brain penetration. These exceptions likely reflect active transport mechanisms (LAT1, organic anion transporters), enabling brain delivery despite physicochemical properties outside traditional CNS drug-likeness criteria, highlighting the importance of experimental validation beyond computational property predictions.

Compounds demonstrating elevated lipophilicity, including sertraline (LogP: 5.29, rank 6), prenylamine (LogP: 4.26, rank 2), duloxetine (LogP: 4.23, rank 3), and donepezil (LogP: 4.26, rank 5), achieved high rankings, despite exceeding optimal LogP thresholds, through compensating factors including minimal polar surface area, favorable hydrogen bonding characteristics, and machine learning BBB probabilities, indicating successful brain penetration despite elevated partition coefficients.

#### 2.12.3. Blood–Brain Barrier Penetration Assessment with Machine Learning Integration

Machine learning BBB prediction analysis provided critical validation of brain penetration potential for top-ranked small molecule candidates, with the majority demonstrating high-confidence predictions supporting therapeutic CNS delivery (Table 9). The integration of ML probability estimates with rule-based BBB scoring enabled nuanced assessment of penetration likelihood, accounting for complex physicochemical relationships beyond individual parameter thresholds.

Twelve of the 15 top candidates (80.0%) achieved machine learning BBB probabilities ≥ 0.8, indicating high-confidence predictions of successful brain penetration, supported by the validated Random Forest classifier (95.7% accuracy, 0.992 AUC-ROC). These high-probability compounds included established CNS drugs with documented clinical brain activity (memantine, donepezil, fluoxetine, sertraline, olanzapine, carbamazepine, valproate), providing empirical validation of ML prediction accuracy and confirming appropriate model calibration. The ML framework successfully identified all established Alzheimer’s disease therapeutics (memantine, donepezil) with probabilities ≥ 0.9, demonstrating robust performance for positive control compounds with unambiguous clinical CNS penetration evidence.

Three candidates demonstrated moderate ML BBB probabilities (0.6–0.8): plerixafor (0.650, rank 1), quetiapine (0.750, rank 8), and topiramate (0.580, rank 11). These moderate predictions reflect physicochemical characteristics approaching CNS drug-likeness boundaries, including elevated polar surface area (plerixafor: 118.4 Å^2^, topiramate: 118.0 Å^2^) or borderline lipophilicity (quetiapine: LogP 2.87). Despite moderate ML probabilities, these compounds achieved favorable overall rankings through strong network evidence and, for topiramate, established clinical CNS efficacy as an FDA-approved anticonvulsant. The retention of moderate-probability compounds in the top rankings reflects the balanced integration approach that considers network evidence, clinical validation, and safety profiles alongside BBB predictions, rather than applying stringent probability cutoffs that might exclude viable therapeutic candidates.

The BBB class distribution analysis revealed a predominance of “High” classifications (10 of 15 candidates, 66.7%), with additional representation in “Moderate High” (4 candidates, 26.7%) and “Moderate” categories (1 candidate, 6.7%), confirming successful CNS-focused filtering and systematic enrichment for brain-penetrant molecules. The absence of “Low” or “Very Low” BBB classifications among the top 15 candidates validates the medicinal chemistry penalty framework, which systematically down-weights compounds with poor brain penetration characteristics in the final rankings.

#### 2.12.4. P-Glycoprotein Efflux Liability and Active Transport Considerations

P-glycoprotein efflux liability assessment revealed that 11 of the 15 top small molecule candidates (73.3%) demonstrated “Moderate” P-gp liability risk based on molecular weight > 400 Da, LogP > 3.0, or HBA ≥ 8 criteria, while 4 candidates (26.7%) achieved “Low” liability classification through favorable physicochemical profiles minimizing efflux transporter recognition (Table 9). The predominance of moderate P-gp liability among the top candidates indicates that active efflux mechanisms represent important considerations for therapeutic CNS delivery, even among compounds with favorable passive BBB permeability characteristics.

Compounds with low P-gp liability include memantine (MW: 179.3 Da, LogP: 3.28, HBA: 1), levetiracetam (MW: 170.2 Da, LogP: −0.64, HBA: 3), gabapentin (MW: 171.2 Da, LogP: −1.10, HBA: 3), topiramate (MW: 339.4 Da, LogP: 0.89, HBA: 9), and valproate (MW: 144.2 Da, LogP: 2.75, HBA: 2). These compounds demonstrate molecular characteristics minimizing P-gp recognition through small molecular size, moderate-to-low lipophilicity, or structural features incompatible with efflux transporter binding pockets. The low P-gp liability classification suggests favorable net CNS accumulation potential, without requirement for co-administration of efflux inhibitors or structural modifications reducing transporter affinity.

Moderate P-gp liability compounds include most top-ranked candidates exceeding molecular weight thresholds or demonstrating elevated lipophilicity characteristics associated with efflux transporter recognition. While moderate liability indicates potential for active efflux, reducing brain concentrations relative to plasma levels, many compounds in this category demonstrate established clinical CNS efficacy (donepezil, sertraline, fluoxetine, risperidone, quetiapine, olanzapine, carbamazepine), confirming that moderate P-gp liability does not preclude therapeutic brain delivery when passive permeability is sufficiently favorable to overcome efflux-mediated clearance. These compounds may benefit from assessment of P-gp inhibitor co-administration strategies or structural optimization, reducing efflux recognition while maintaining target engagement and BBB permeability.

#### 2.12.5. Chemical Tractability and Development Feasibility Profile

Chemical tractability assessment revealed exceptional development feasibility among the top-ranked small molecule candidates, with 14 of 15 compounds (93.3%) achieving Class I (Highly Tractable) classification through simultaneous satisfaction of favorable BBB penetration, acceptable chemical reactivity, and molecular weight compliance (Table 9).

Class I compounds demonstrate comprehensive pharmaceutical suitability, requiring minimal optimization for CNS therapeutic applications, possessing favorable BBB penetration characteristics (High or Moderate High ML probability), low chemical reactivity risk profiles, and molecular weights compatible with passive diffusion mechanisms. The predominance of Class I classification among the top candidates reflects the successful CNS pre-filtering, eliminating compounds with severe pharmaceutical liabilities, combined with the medicinal chemistry penalty framework systematically down-weighting candidates with development challenges in the final rankings.

Topiramate (rank 11) represents the single Class II (Moderately Tractable) compound among top 15 candidates, requiring targeted optimization to address elevated polar surface area (118.0 Å^2^) and moderate ML BBB probability (0.580), despite established clinical CNS efficacy as an FDA-approved anticonvulsant. The Class II classification reflects the balanced assessment approach that maintains therapeutic opportunities for compounds with defined development challenges when strong clinical validation or compelling network evidence supports continued consideration. Topiramate’s established CNS activity, despite suboptimal physicochemical properties, suggests either active transport mechanisms or sufficient passive permeability to achieve therapeutic brain concentrations, warranting mechanistic investigation of BBB penetration pathways to inform development strategies for structurally related compounds.

Chemical reactivity assessment revealed universally favorable safety profiles, with all 15 top small molecule candidates demonstrating “Low” reactivity risk, indicating minimal potential for off-target protein modification, metabolic liability, or cellular toxicity mechanisms. This exceptional safety profile provides confidence for clinical development prioritization and supports the feasibility of rapid therapeutic translation through established regulatory pathways with known safety characteristics for approved compounds or predictable safety profiles for experimental agents.

#### 2.12.6. Alzheimer’s Disease Evidence Classification and Translational Readiness

Systematic classification of Alzheimer’s-disease-specific evidence for top-ranked small molecule candidates revealed a predominance of “Mechanistic” evidence levels (13 of 15 candidates, 86.7%), with limited representation of “Established” therapeutic validation (2 of 15 candidates, 13.3%), highlighting the largely exploratory nature of computational drug repurposing predictions, requiring experimental validation in AD-relevant model systems (Table 9).

Memantine (rank 4) and donepezil (rank 5) represent the only candidates with “Established” AD evidence classification, reflecting their FDA-approved status for Alzheimer’s disease treatment, with documented clinical efficacy in randomized controlled trials. The identification of these positive control compounds among the top-ranked candidates validates the network medicine framework’s capacity to successfully prioritize therapeutically relevant targets, as both drugs emerged through systematic integration of network proximity to MNPTD-identified genes (IGF1, GRIA1, GRIN2A) with favorable pharmaceutical properties. Memantine’s NMDA receptor antagonism and donepezil’s acetylcholinesterase inhibition provide established symptomatic benefit for AD patients, though neither compound demonstrates disease-modifying effects on underlying pathology.

The 13 candidates classified with “Mechanistic” evidence lack direct experimental validation in Alzheimer’s disease models but demonstrate therapeutic rationale based on pathway engagement relevant to AD pathogenesis. However, it is critical to acknowledge that mechanistic rationale alone does not guarantee therapeutic efficacy, and these predictions remain speculative hypotheses requiring systematic validation in AD-specific cellular and animal models before clinical translation:

Plerixafor (rank 1, *CXCR4* antagonist): While FDA-approved for stem cell mobilization, plerixafor’s potential AD relevance is based on CXCR4’s role in neuroinflammation and microglial activation pathways implicated in disease progression. No preclinical or clinical data currently demonstrate efficacy in AD models. The therapeutic hypothesis requires validation through experiments assessing effects on amyloid-*β* pathology, tau phosphorylation, neuroinflammation markers, and cognitive outcomes in transgenic AD mice before clinical consideration.

Duloxetine (rank 3, SNRI antidepressant): The serotonin-norepinephrine reuptake inhibitor demonstrates established antidepressant efficacy but lacks AD-specific validation. Mechanistic rationale involves modulation of monoaminergic systems implicated in cognitive function and potential anti-inflammatory effects through serotonin receptor signaling. Therapeutic benefit for AD core pathology remains unproven, requiring assessment in APP/PS1 mice or 3xTg-AD models with cognitive, pathological, and biomarker endpoints.

Sertraline (rank 6, SSRI antidepressant): Similarly to duloxetine, sertraline’s AD relevance is based on serotonergic modulation of cognitive circuits and potential neuroprotective effects through *BDNF* upregulation. No direct evidence supports disease-modifying activity in AD, necessitating systematic evaluation in validated preclinical models before repurposing consideration.

The predominance of mechanistic-only evidence among the top candidates emphasizes the critical distinction between computational predictions and experimentally validated therapeutics. Network proximity does not guarantee therapeutic efficacy, and the identified candidates should be considered prioritized hypotheses for experimental testing rather than definitive therapeutic recommendations. Successful translation requires systematic validation pipelines, including (1) target engagement confirmation in neuronal cell models; (2) effects on AD-relevant pathologies (A*β* aggregation, tau phosphorylation, synaptic dysfunction) in human iPSC-derived neurons or brain organoids; (3) cognitive and pathological outcomes in transgenic AD mouse models (APP/PS1, 5xFAD, 3xTg-AD); and (4) biomarker-supported proof-of-concept clinical trials in prodromal or early AD populations.

### 2.13. Peptide and Biologic Therapeutic Candidates with Specialized Delivery Requirements

Systematic application of modality-specific ranking criteria to the 73 peptide and 3 biologic candidates identified through CNS-focused network analysis generated a separate prioritization framework acknowledging the fundamentally distinct blood–brain barrier penetration mechanisms and pharmaceutical development requirements for these large-molecule therapeutics. Unlike small molecules that achieve brain delivery through passive lipophilic diffusion, peptides and biologics require active transport mechanisms, including carrier-mediated transcytosis (LAT1, PEPT transporters), receptor-mediated transcytosis (transferrin receptor, insulin receptor, LRP-1), or specialized BBB shuttle technologies, for therapeutic CNS concentrations.

#### 2.13.1. Peptide-Specific Ranking Methodology and BBB Penetration Considerations

Peptide candidates underwent systematic ranking based on network evidence quality with reduced medicinal chemistry penalties, reflecting transporter-mediated BBB penetration mechanisms that enable brain delivery despite physicochemical properties incompatible with passive diffusion. The ranking framework accommodated elevated polar surface area (PSA ≤ 200 Å^2^), broader lipophilicity ranges (−2.0≤ LogP ≤2.0), and increased hydrogen bonding capacity (HBD ≤ 8, HBA ≤ 15) characteristic of peptides achieving CNS penetration through endogenous amino acid transporters or receptor-mediated uptake mechanisms.

The peptide-specific BBB assessment integrated multiple considerations beyond passive permeability predictions: (1) structural similarity to known CNS-penetrant neuropeptides (substance P, enkephalins, vasopressin analogs), (2) potential for LAT1 transporter recognition based on large neutral amino acid content, (3) metabolic stability considerations including proteolytic cleavage susceptibility, and (4) molecular weight compatibility with peptide transporter systems (PEPT1/2) or receptor-mediated transcytosis pathways. The machine learning BBB predictions for peptides require cautious interpretation, as the training dataset comprised predominantly small molecules with limited peptide representation, potentially underestimating brain penetration probability for compounds leveraging active transport mechanisms not captured in passive permeability descriptors.

#### 2.13.2. Top-Ranked Peptide Therapeutic Candidates with Comprehensive Assessment

Detailed characterization of the top 10 peptide candidates revealed diverse molecular characteristics spanning both small peptide-like molecules approaching conventional drug space and larger peptidic structures requiring specialized delivery considerations (Table 10). The systematic evaluation demonstrated substantial heterogeneity in physicochemical properties, BBB penetration predictions, and development feasibility profiles compared to the more homogeneous small molecule category, reflecting the emerging and technically challenging nature of peptide-based CNS therapeutic development.

#### 2.13.3. Trofinetide: Top-Ranked Peptide Candidate with Critical Translational Caveats

Trofinetide emerged as the highest-ranked peptide candidate, with a medicinal chemistry-adjusted network score of 1.387, representing an FDA-approved synthetic analog of glycyl-L-2-methylprolyl-L-glutamic acid (GPE), the C-terminal tripeptide of insulin-like growth factor-1 (IGF-1), currently indicated for Rett syndrome treatment. The compound’s exceptional ranking reflects optimal integration of strong network evidence linking *IGF1* pathway modulation to neuroprotective mechanisms, favorable physicochemical properties approaching small molecule characteristics (MW: 341.4 Da, LogP: 1.89, PSA: 45.2 Å^2^), and high machine learning BBB penetration probability (0.917), suggesting compatibility with passive diffusion or facilitated transport mechanisms.

Despite trofinetide’s favorable computational ranking and established CNS penetration documented in Rett syndrome clinical trials, its classification as “Speculative” for Alzheimer’s disease applications warrants explicit clarification and transparent communication of substantial evidence gaps requiring experimental validation before clinical translation.

Trofinetide demonstrates no preclinical or clinical evidence supporting efficacy in Alzheimer’s disease models or patients. The therapeutic hypothesis is derived exclusively from computational network proximity to *IGF1* and synaptic dysfunction pathways, without experimental validation of effects on core AD pathologies including amyloid-*β* aggregation, tau phosphorylation, neuroinflammation, synaptic loss, or cognitive decline in transgenic AD mice (APP/PS1, 3xTg-AD, 5xFAD) or human AD-derived cellular models (iPSC neurons, brain organoids).

While trofinetide demonstrates neuroprotective effects and synaptic stabilization in Rett syndrome through IGF-1 pathway modulation, Rett syndrome pathogenesis (*MECP2* mutations, transcriptional dysregulation) differs fundamentally from Alzheimer’s disease mechanisms (amyloid cascade, tauopathy, neuroinflammation). Neuroprotective efficacy in one neurodevelopmental disorder does not predict therapeutic benefit for a distinct neurodegenerative proteinopathy, necessitating AD-specific mechanistic validation.

Despite favorable physicochemical properties approaching small molecule ranges, trofinetide’s peptidic structure (tripeptide with modified proline residue) may involve transporter-mediated BBB delivery through peptide transporters (PEPT1/2) or amino acid carriers (LAT1) rather than purely passive diffusion. This delivery mechanism distinction, while not precluding therapeutic brain concentrations, requires mechanistic characterization and may necessitate dose optimization strategies differing from conventional small molecule approaches. The compound’s classification as a peptide reflects structural considerations and potential regulatory classification, rather than molecular weight alone.

Trofinetide’s FDA approval for Rett syndrome provides established safety profiles and regulatory precedent, facilitating repurposing initiatives compared to experimental compounds. However, optimal dosing regimens, treatment duration requirements, and biomarker-guided patient selection strategies for Alzheimer’s disease applications remain undefined without AD-specific clinical investigation. The oral bioavailability and CNS penetration characteristics documented in Rett syndrome provide encouraging pharmacokinetic precedent but require validation of therapeutically relevant brain concentrations for AD pathology modification.

Translation of trofinetide from computational prediction to viable AD therapeutic candidate requires systematic experimental progression: (1) in vitro assessment of effects on A*β* aggregation kinetics, tau phosphorylation status, and synaptic protein expression in human neuronal cultures or brain organoids exposed to AD-relevant stressors; (2) dose–response characterization in transgenic AD mouse models, evaluating cognitive outcomes (Morris water maze, novel object recognition), pathological burden (A*β* plaques, tau tangles, synaptic density), and mechanistic biomarkers (IGF-1 signaling activation, synaptic plasticity markers); and (3) biomarker-supported proof-of-concept clinical trials in prodromal or early AD populations with CSF/plasma biomarker monitoring (A*β*_42_, total tau, phospho-tau, neurofilament light) and cognitive assessments.

#### 2.13.4. Somatostatin Analogs and Large Peptide Hormones: Development Challenges

Ranks 3–10 in the peptide category encompass somatostatin analogs (octreotide, lanreotide, pasireotide) and larger peptide hormones (vasoactive intestinal peptide, glucagon, insulin lispro, exenatide) demonstrating substantial molecular weights (1019–5808 Da), extensive hydrogen bonding networks, and highly negative LogP values reflecting pronounced hydrophilicity (Table 10). These compounds achieved favorable network evidence scores but demonstrated severe BBB penetration limitations, reflected in very low machine learning probabilities (0.015–0.320) and predominantly Class III-IV tractability classifications, indicating substantial development challenges requiring specialized delivery technologies.

Somatostatin analogs (ranks 3–6) comprise FDA-approved therapeutics for acromegaly and neuroendocrine tumors, demonstrating established safety profiles and manufacturing infrastructure. The mechanistic rationale for AD applications involves somatostatin-receptor-mediated modulation of neuroinflammation and potential effects on amyloid-*β* metabolism through receptor signaling cascades. However, molecular weights exceeding 1000 Da combined with extensive polar surface areas (267–456 Å^2^) preclude passive BBB penetration, necessitating receptor-mediated transcytosis engineering, BBB shuttle conjugation, or focused ultrasound-mediated delivery for therapeutic brain concentrations. These compounds represent Class III (Challenging) development opportunities, requiring advanced delivery technologies and substantial resource commitments for CNS applications.

Large peptide hormones (ranks 7–10), including vasoactive intestinal peptide (3326 Da), glucagon (3483 Da), insulin lispro (5808 Da), and exenatide (4186 Da), demonstrated severe BBB penetration limitations, reflected in very low ML probabilities (<0.06) and Class IV (Currently Intractable) classifications. These compounds achieved favorable network rankings through engagement of growth factor signaling, metabolic regulation, and neuroprotective pathways relevant to AD pathogenesis, but molecular sizes exceeding 3000 Da combined with extreme hydrophilicity (LogP: −5.89 to −8.45) render conventional BBB penetration approaches infeasible. Therapeutic CNS delivery would require specialized technologies, including brain-penetrant antibody shuttles conjugated to peptide payloads, receptor-mediated transcytosis engineering, or invasive delivery approaches (intracerebroventricular administration, convection-enhanced delivery). The Class IV classification reflects current technical limitations rather than fundamental biological invalidity, suggesting these targets may warrant reconsideration as BBB shuttle technologies advance.

#### 2.13.5. Biologic Therapeutic Candidates and Receptor-Mediated Transcytosis Requirements

The three biologic candidates identified through network analysis comprise large protein therapeutics or monoclonal antibodies requiring receptor-mediated transcytosis for BBB penetration (Table 11). Prasinezumab (rank 1 among biologics, investigational anti-α-synuclein antibody) achieved the highest network score among large molecule therapeutics through direct engagement of protein aggregation pathways shared between Parkinson’s disease and Alzheimer’s disease. A molecular weight exceeding 145,000 Da precludes passive BBB penetration, necessitating either engineered BBB shuttle technologies (bispecific antibodies targeting transferrin receptor for transcytosis) or focused ultrasound approaches, transiently disrupting tight junctions for antibody delivery.

Gantenerumab and aducanumab (ranks 2–3 among biologics) represent anti-amyloid-*β* antibodies with direct AD clinical development history, providing established precedents for antibody-based AD therapeutics, despite modest clinical efficacy and significant safety concerns (amyloid-related imaging abnormalities, ARIA-E/ARIA-H). These compounds achieved network rankings through direct engagement of amyloid cascade pathways, with aducanumab’s controversial FDA approval providing regulatory precedent for amyloid-targeting biologics, despite marginal clinical benefits and substantial scientific criticism regarding approval justification.

The minimal biologic representation (n=3, 0.08% of total candidates) reflects substantial technical barriers to brain-penetrant antibody development and the limited availability of CNS-validated biologics within the DGIdb database. Future biologic identification may benefit from integration of specialized antibody databases (Therapeutic Antibody Database, Antibody Society repositories) and systematic assessment of BBB shuttle engineering potential for promising extracellular AD targets including A*β* oligomers, tau species, and neuroinflammatory mediators.

#### 2.13.6. Comparative Assessment: Peptides Versus Small Molecules for AD Applications

The systematic comparison between peptide and small molecule therapeutic categories revealed fundamental trade-offs informing development strategy selection for Alzheimer’s disease drug repurposing initiatives. Small molecules demonstrate superior BBB penetration characteristics (83.6% High/Moderate High BBB classification), manufacturing scalability, oral bioavailability potential, and compatibility with conventional pharmaceutical development infrastructure, supporting their predominance (97.97%) in CNS therapeutic discovery. However, small molecules face limitations, including restricted chemical diversity, challenges targeting protein–protein interactions or allosteric sites, and potential for off-target effects across large pharmacological space.

Peptides offer complementary advantages, including high target selectivity, reduced off-target toxicity risks, access to protein–protein interaction interfaces undruggable by small molecules, and potential for allosteric modulation of complex biological systems. However, peptide therapeutics face substantial development challenges, including proteolytic instability requiring chemical modifications (D-amino acids, backbone cyclization, N-methylation), BBB penetration limitations necessitating transporter targeting or specialized delivery, and manufacturing complexity with higher cost-of-goods relative to small molecules. The 1.95% peptide representation reflects these technical challenges, while highlighting emerging opportunities as peptide engineering technologies advance.

### 2.14. Alzheimer’s Disease Evidence Assessment and Translational Plausibility

Systematic evaluation of Alzheimer’s-disease-specific evidence for top-ranked drug repurposing candidates across all therapeutic modalities reveals substantial heterogeneity in validation strength, ranging from FDA-approved AD therapeutics with established clinical efficacy to purely computational predictions lacking any experimental support in disease-relevant model systems. This comprehensive evidence assessment addresses critical translational feasibility concerns by providing transparent classification of therapeutic rationale strength, explicit identification of evidence gaps requiring experimental validation, and realistic appraisal of clinical translation prospects for candidates spanning the spectrum from established therapeutics to speculative hypotheses. The stratified evidence framework prevents conflation of computationally predicted candidates with experimentally validated therapeutics, enabling evidence-based prioritization of development investments and systematic identification of experimental validation requirements for promising network-derived predictions.

#### 2.14.1. Evidence Classification Framework and Assessment Criteria

Drug candidates underwent systematic classification into five hierarchical evidence categories, reflecting progressive validation strength from computational predictions through clinical proof-of-concept in Alzheimer’s disease populations (Table 12). The classification framework balances recognition of therapeutic potential identified through network analysis with transparent acknowledgment of evidence limitations and experimental validation requirements.

Established Evidence characterizes FDA-approved therapeutics demonstrating clinical efficacy in randomized, placebo-controlled trials in Alzheimer’s disease populations with regulatory validation and documented effects on cognitive, functional, or biomarker endpoints. This category encompasses memantine (NMDA receptor antagonist), donepezil (acetylcholinesterase inhibitor), rivastigmine (acetylcholinesterase/butyrylcholinesterase inhibitor), and galantamine (acetylcholinesterase inhibitor/nicotinic receptor modulator), representing positive controls validating the network medicine framework’s capacity to identify therapeutically relevant compounds. However, established therapeutics provide only symptomatic benefit, without disease-modifying effects on underlying amyloid, tau, or neuroinflammatory pathologies, highlighting the need for novel therapeutic mechanisms addressing core disease processes.

Clinical Evidence encompasses compounds with active or completed clinical trials specifically in Alzheimer’s disease populations (ClinicalTrials.gov registration), demonstrating sufficient preclinical validation and safety profiles to warrant human investigation but lacking demonstration of definitive efficacy or regulatory approval. This category includes investigational therapeutics targeting amyloid pathology (aducanumab, gantenerumab), tau aggregation, neuroinflammation, or synaptic dysfunction, representing advanced-stage development candidates with substantial clinical investment but uncertain efficacy, pending trial completion or regulatory review.

Preclinical Evidence characterizes compounds demonstrating therapeutic effects in validated Alzheimer’s disease animal models (APP/PS1, 3xTg-AD, 5xFAD transgenic mice, rat models) or human-derived cellular systems (iPSC-derived neurons, brain organoids, primary neuronal cultures) with documented effects on AD-relevant pathologies including amyloid-*β* aggregation, tau phosphorylation, synaptic loss, neuroinflammation, or cognitive deficits. This category represents experimentally validated hypotheses demonstrating proof-of-principle efficacy in disease-relevant model systems, warranting clinical translation consideration following additional pharmacokinetic, toxicological, and formulation development.

Mechanistic Evidence encompasses compounds lacking direct experimental validation in Alzheimer’s disease models but demonstrating therapeutic rationale based on target engagement of pathways implicated in disease pathogenesis, including synaptic dysfunction, neurotransmitter imbalance, neuroinflammation, oxidative stress, mitochondrial dysfunction, or cerebrovascular pathology. The mechanistic category acknowledges biological plausibility, while recognizing that pathway involvement does not guarantee therapeutic efficacy, necessitating systematic validation in AD-specific model systems before clinical consideration. This category comprises the majority of network-derived predictions, reflecting the exploratory nature of computational drug repurposing.

Speculative Evidence represents purely computational predictions derived from network proximity analysis, without experimental validation or established mechanistic links to Alzheimer’s disease pathogenesis. The speculative category acknowledges compounds achieving favorable network rankings through statistical associations with AD-relevant genes but lacking biological rationale or experimental support, requiring comprehensive mechanistic investigation and systematic validation before therapeutic development consideration. This transparent classification prevents premature clinical translation of insufficiently validated computational predictions, while maintaining opportunities for experimental investigation of potentially novel therapeutic mechanisms.

#### 2.14.2. Established Therapeutics: Validation of Network Medicine Predictions

The identification of memantine (rank 4 among small molecules) and donepezil (rank 5 among small molecules) among the top computational predictions validates the network medicine framework’s capacity to successfully prioritize therapeutically relevant compounds through systematic integration of network proximity analysis with medicinal chemistry assessment. These FDA-approved Alzheimer’s disease therapeutics emerged through network-based targeting of glutamate receptor systems (memantine: NMDA antagonism via *GRIN2A* engagement) and cholinergic neurotransmission (donepezil: acetylcholinesterase inhibition enhancing synaptic acetylcholine availability), demonstrating that computational approaches can effectively identify clinically validated therapeutic mechanisms when properly integrated with CNS-focused filtering and pharmaceutical property assessment.

Memantine provides modest symptomatic benefit for moderate-to-severe Alzheimer’s disease through noncompetitive NMDA receptor antagonism, reducing excitotoxicity from chronic glutamate receptor overactivation, while preserving physiological synaptic transmission through voltage-dependent channel kinetics favoring pathological over normal neuronal activity [42]. The compound’s network ranking reflects optimal integration of strong biological evidence (*GRIN2A*, *GRIA1* network proximity), excellent CNS penetration characteristics (MW: 179.3 Da, LogP: 3.28, BBB ML probability: 0.950), and established safety profile, enabling rapid clinical investigation. However, memantine demonstrates no disease-modifying effects on amyloid-*β* accumulation, tau pathology, or neurodegeneration progression, representing symptomatic intervention without alteration of underlying pathophysiology.

Donepezil achieves modest cognitive benefit across mild-to-severe disease stages through reversible acetylcholinesterase inhibition, enhancing cholinergic neurotransmission, compensating for the basal forebrain cholinergic neuron degeneration characteristic of Alzheimer’s disease progression [43]. The compound’s favorable ranking integrates strong network evidence with excellent pharmaceutical properties (MW: 379.5 Da, LogP: 4.26, BBB ML probability: 0.910) and established clinical utility. Like memantine, donepezil provides symptomatic relief without disease modification, with cognitive benefits typically modest (2–3 point ADAS-Cog improvement) and transient, declining as neurodegeneration progresses, despite continued treatment.

The successful computational identification of established AD therapeutics provides critical validation that network medicine approaches can effectively capture biologically relevant therapeutic mechanisms. However, the limitation of current FDA-approved drugs to symptomatic interventions without disease modification underscores the need for novel therapeutic mechanisms targeting core pathological processes including amyloid aggregation, tau phosphorylation, neuroinflammation, synaptic loss, and neuronal death, precisely the therapeutic opportunities represented by mechanistic and speculative candidates requiring experimental validation.

#### 2.14.3. Mechanistic Evidence Candidates: Biological Plausibility Without AD-Specific Validation

The majority of the top-ranked candidates across all therapeutic modalities possess mechanistic evidence classification, indicating biological rationale based on pathway engagement relevant to Alzheimer’s disease pathogenesis but lacking direct experimental validation in AD-specific model systems. This evidence category encompasses diverse therapeutic mechanisms, including neuroinflammation modulation (plerixafor), monoaminergic neurotransmission enhancement (duloxetine, sertraline), dopaminergic/serotonergic receptor antagonism (risperidone, quetiapine), and protein aggregation inhibition (prasinezumab), representing hypotheses with biological plausibility requiring systematic experimental investigation.

Plerixafor (*CXCR4* Antagonist): The top-ranked small molecule candidate achieved favorable network scores through *CXCR4* engagement, a chemokine receptor implicated in neuroinflammatory signaling, microglial activation, and potentially neurotoxic inflammatory cascades contributing to Alzheimer’s disease progression. The FDA-approved status for stem cell mobilization provides established safety profiles and known pharmacokinetics facilitating rapid clinical investigation. However, plerixafor demonstrates no experimental validation in Alzheimer’s disease cellular or animal models, with a therapeutic hypothesis derived exclusively from computational network proximity, without mechanistic confirmation. The compound requires systematic assessment of effects on microglia-mediated neuroinflammation, amyloid-*β* pathology, tau phosphorylation, and cognitive outcomes in transgenic AD mice before clinical translation consideration. Additionally, brain pharmacokinetics following systemic administration require characterization, as plerixafor’s modest BBB ML probability (0.650) and elevated molecular weight (502.8 Da) suggest potential limitations in achieving therapeutic CNS concentrations.

Duloxetine and Sertraline (Antidepressants): These serotonin-norepinephrine (duloxetine) and selective serotonin (sertraline) reuptake inhibitors achieved favorable rankings through network proximity to monoaminergic neurotransmitter systems implicated in cognitive function, with mechanistic hypotheses involving enhancement of prefrontal–hippocampal circuits, supporting memory consolidation and potential anti-inflammatory effects through serotonin receptor signaling modulation. However, clinical trial data for sertraline in Alzheimer’s disease demonstrate no cognitive benefit, providing direct evidence that antidepressant mechanisms are insufficient for disease modification despite biological plausibility. The failed translation highlights the critical limitations of mechanistic rationale without experimental validation, emphasizing that pathway involvement does not guarantee therapeutic efficacy. Duloxetine lacks even failed clinical trial data, representing purely hypothetical therapeutic potential, requiring comprehensive preclinical investigation before clinical consideration, despite favorable network rankings.

Atypical Antipsychotics (Risperidone, Quetiapine): These dopamine/serotonin receptor antagonists achieved network rankings through engagement of neurotransmitter systems and potential utility for behavioral symptom management (agitation, psychosis) in moderate-to-severe Alzheimer’s disease. Clinical experience with antipsychotics in AD populations demonstrates efficacy for behavioral symptoms but no cognitive benefit and significant safety concerns, including increased mortality risk, cerebrovascular events, and amyloid-related imaging abnormalities (ARIA). The mechanistic evidence classification reflects established pharmacological rationale for symptomatic management, without disease-modifying potential, with network rankings primarily capturing behavioral symptom utility rather than core pathology intervention.

Prasinezumab (Anti-α-Synuclein Antibody): The top-ranked biologic candidate targets α-synuclein aggregation, a pathological hallmark of Parkinson’s disease and dementia with Lewy bodies. The mechanistic hypothesis for Alzheimer’s disease applications involves potential synuclein-amyloid-*β* interactions and overlapping protein aggregation pathways between synucleinopathies and amyloid-related dementias. However, prasinezumab is optimized for Parkinson’s disease pathology, with uncertain relevance to Alzheimer’s disease mechanisms dominated by amyloid and tau pathologies rather than synuclein aggregation. The compound faces additional challenges, including antibody BBB delivery requiring receptor-mediated transcytosis engineering and substantial development timelines (7–10 years) for CNS-targeted biologic applications.

The mechanistic evidence category highlights both the opportunities and limitations of computational drug repurposing. Biological plausibility provides a rational foundation for experimental investigation, but mechanistic hypotheses without AD-specific validation demonstrate high failure risk in clinical translation, as evidenced by sertraline’s failed efficacy trials, despite compelling neurotransmitter modulation rationale. Systematic preclinical validation in transgenic AD mice with cognitive, pathological, and biomarker endpoints represents the critical bottleneck separating mechanistic hypotheses from viable therapeutic candidates warranting clinical investment.

#### 2.14.4. Speculative Evidence: Trofinetide and the Limits of Computational Prediction

Trofinetide’s classification as speculative evidence, despite achieving the highest overall network score (1.387), exemplifies the critical limitations of purely computational drug repurposing approaches and necessitates explicit acknowledgment of substantial evidence gaps separating algorithmic predictions from validated therapeutic candidates. The compound’s exceptional ranking reflects optimal integration of network proximity to *IGF1* pathway genes with favorable physicochemical properties and established CNS penetration in Rett syndrome populations. However, trofinetide demonstrates complete absence of Alzheimer’s-disease-specific experimental or clinical data, with a therapeutic hypothesis derived exclusively from statistical network associations rather than mechanistic investigation or empirical validation.

The speculative classification reflects four critical evidence limitations requiring transparent communication:

(1) Trofinetide’s FDA approval for Rett syndrome (*MECP2* mutations causing transcriptional dysregulation and synaptic dysfunction) does not predict efficacy for Alzheimer’s disease pathogenesis involving fundamentally distinct mechanisms (amyloid cascade, tauopathy, neuroinflammation, synaptic loss). While both disorders involve synaptic dysfunction, the molecular mechanisms, developmental versus degenerative timecourses, and therapeutic intervention points differ substantially. Neuroprotective effects in neurodevelopmental transcriptional dysregulation do not necessarily translate to neuroprotection in neurodegenerative proteinopathies.

(2) Trofinetide lacks any published preclinical data in transgenic Alzheimer’s disease mouse models (APP/PS1, 3xTg-AD, 5xFAD), tau transgenic models, or human AD-derived cellular systems (iPSC neurons, brain organoids). Without experimental evidence demonstrating effects on amyloid-*β* aggregation, tau phosphorylation, synaptic protein expression, neuroinflammation markers, or cognitive outcomes in AD-relevant model systems, the therapeutic hypothesis remains purely computational, without empirical foundation. The speculative classification acknowledges this fundamental validation gap.

(3) While trofinetide is described as an IGF-1 pathway modulator, the precise molecular targets, receptor interactions, and downstream signaling cascades mediating Rett syndrome efficacy remain incompletely characterized. IGF-1 signaling complexity involves multiple receptor subtypes (IGF-1R, insulin receptor hybrids), diverse downstream pathways (PI3K/AKT, MAPK/ERK, mTOR), and context-dependent effects varying across developmental stages and cell types. Without mechanistic characterization of trofinetide’s AD-relevant target engagement, optimal dosing regimens, biomarker-guided patient selection strategies, and combination therapy approaches remain undefined.

(4) Despite favorable Rett syndrome clinical data establishing safety and CNS penetration, optimal dose ranges, treatment duration requirements, and patient population selection criteria for Alzheimer’s disease applications remain unknown. Rett syndrome dosing (200 mg/kg twice daily oral administration) may not achieve therapeutically relevant brain concentrations for AD pathology modification. Biomarker-guided proof-of-concept trial designs, appropriate cognitive endpoints, and treatment effect timelines require definition through preclinical investigation before clinical translation.

The speculative evidence classification for trofinetide, despite the highest overall computational ranking, demonstrates the framework’s commitment to transparent evidence communication and realistic appraisal of translational prospects. Network proximity represents a hypothesis-generating tool identifying promising candidates for experimental investigation rather than definitive therapeutic validation. Trofinetide warrants systematic preclinical assessment in AD models based on compelling computational evidence and favorable pharmaceutical properties, but should be communicated as a prioritized hypothesis for experimental testing rather than validated therapeutic recommendation, pending generation of AD-specific efficacy data.

#### 2.14.5. Evidence-Based Development Prioritization and Resource Allocation

The hierarchical evidence classification enables rational prioritization of development investments aligned with validation strength, risk tolerance, and organizational capabilities. Immediate clinical investigation opportunities encompass established therapeutics for combination therapy assessment, dosing optimization, or biomarker-guided patient selection strategies, leveraging known safety profiles and regulatory precedents for rapid clinical translation. Clinical evidence candidates warrant continued investment for trial completion and regulatory advancement, representing substantial prior resource commitments, with realistic approval prospects.

Mechanistic evidence candidates require systematic preclinical validation pipelines before clinical consideration, with development prioritization favoring compounds demonstrating (1) strong network evidence combined with favorable pharmaceutical properties, (2) established safety profiles from approved indications enabling rapid repurposing, (3) druggable targets with validated assay systems for target engagement confirmation, and (4) mechanistic rationale addressing core AD pathologies (amyloid, tau, neuroinflammation) rather than purely symptomatic mechanisms. The recommended validation workflow encompasses in vitro target engagement confirmation, AD-relevant cellular model assessment (iPSC neurons, brain organoids), transgenic mouse model efficacy evaluation with cognitive and pathological endpoints, and biomarker-supported proof-of-concept clinical trials in prodromal or early AD populations.

Speculative candidates warrant exploratory investigation only when (1) exceptionally strong computational evidence justifies resource allocation despite validation gaps, (2) favorable pharmaceutical properties and established safety profiles minimize development risks, (3) organizational capabilities support systematic preclinical validation pipelines, and (4) alternative therapeutic mechanisms offer potential advantages over established approaches. The high failure risk for speculative candidates necessitates conservative resource allocation, with clear go/no-go decision criteria based on demonstration of preclinical efficacy before clinical investment.

## 3. Discussion

### 3.1. Systems Biology Approach to Alzheimer’s Disease Drug Repurposing

This study presents a comprehensive medicinal-chemistry-guided network medicine framework that systematically addresses the critical gap between computational drug discovery predictions and practical pharmaceutical development requirements. The integration of CNS-focused pre-filtering, systematic blood–brain barrier assessment, chemical tractability classification, and safety evaluation represents a paradigm shift from purely algorithmic approaches toward clinically actionable therapeutic recommendations. Our findings demonstrate that the strategic combination of network-based evidence with rigorous medicinal chemistry assessment generates drug repurposing candidates with both strong biological rationale and realistic development prospects for Alzheimer’s disease intervention.

The CNS-focused filtering strategy successfully refined the pharmacological search space from 24,474 total compounds to 8247 CNS-relevant drugs, while enhancing rather than compromising predictive accuracy. This 66.3% reduction in computational complexity enabled implementation of sophisticated network algorithms, while maintaining comprehensive coverage of therapeutically relevant chemical space. The filtering effectiveness is validated by the exceptional pharmaceutical characteristics of the identified candidates, with 64.8% achieving Class I (Highly Tractable) status and 83.6% demonstrating favorable blood–brain barrier penetration potential.

The cross-dataset validation approach from the original transcriptomic analysis yielded 742 robustly dysregulated genes, which provided the molecular foundation for network-based target identification. This gene set exhibited remarkable functional coherence, with pathway enrichment analysis revealing predominant involvement in synaptic transmission, neurotransmitter signaling, and cellular communication processes that strongly support the synaptic dysfunction hypothesis of Alzheimer’s disease [53,54]. The integration of these validated biological targets with CNS-focused pharmacological databases created an unprecedented opportunity for systematic identification of brain-penetrant therapeutic candidates with established mechanistic relevance.

### 3.2. Medicinal Chemistry Integration and Pharmaceutical Feasibility Assessment

The systematic integration of medicinal chemistry considerations with network-based predictions addressed fundamental limitations of traditional computational drug discovery approaches, which often generate promising algorithmic results with poor translational potential. Our medicinal chemistry framework evaluated 3743 network-derived candidates across multiple pharmaceutical development criteria, including molecular properties, blood–brain barrier penetration, chemical reactivity, and overall tractability for CNS applications.

The blood–brain barrier assessment revealed highly favorable characteristics, with 83.6% of candidates achieving High or Moderate High BBB penetration classifications, substantially exceeding typical pharmaceutical screening results, where CNS-suitable compounds often represent less than 20% of random collections [55,56]. The molecular property analysis demonstrated excellent alignment with established CNS drug-likeness criteria, with 95.4% of compounds maintaining molecular weights below 450 Da and 66.0% achieving optimal lipophilicity ranges (LogP 1.5–3.5) for brain penetration [57,58].

Chemical tractability classification provided critical guidance for development prioritization through systematic evaluation of pharmaceutical feasibility. The identification of 2427 compounds (64.8%) as Class I (Highly Tractable) indicates unprecedented success in identifying immediately viable therapeutic candidates requiring minimal optimization for CNS applications. This exceptional tractability profile validates the strategic CNS-focused approach and demonstrates successful enrichment for compounds with comprehensive pharmaceutical suitability.

The safety assessment addressed critical concerns regarding chemical reactivity and off-target interactions through systematic evaluation of molecular structural features associated with potential toxicity. The analysis revealed highly favorable safety characteristics, with 99.8% of candidates demonstrating acceptable reactivity profiles, including 93.5% classified as low risk and only 0.2% requiring high-risk designation.

### 3.3. Machine Learning Validation of Blood–Brain Barrier Prediction Framework

The implementation of machine learning-based BBB penetration validation represents a critical methodological advancement, addressing longstanding concerns regarding the reliability of rule-based computational permeability predictions. Our supervised learning approach, trained on 110 experimentally validated CNS-penetrant and non-penetrant drugs with balanced class representation (70 CNS+, 40 CNS-), achieved exceptional predictive performance, substantially exceeding published BBB model accuracies reported in the literature.

The systematic evaluation of four complementary machine learning algorithms (Random Forest, Gradient Boosting, XGBoost, Support Vector Machine) demonstrated consistently high performance across diverse mathematical frameworks, with accuracy values ranging from 91.3% to 95.7% and AUC-ROC values spanning 0.938 to 0.992. Random Forest classification emerged as the optimal model, based on balanced accuracy (95.7%), perfect sensitivity (100%), specificity of 87.5%, and exceptional discriminatory power reflected in an AUC-ROC of 0.992. These performance metrics substantially exceed typical BBB prediction accuracies reported in the literature (70–85%) [59,60], validating the effectiveness of the curated validation dataset and minimal five-feature descriptor strategy (molecular weight, LogP, PSA, HBD, HBA).

The perfect sensitivity achieved across all four models represents a critical strength for therapeutic candidate prioritization applications, ensuring that no potentially viable brain-penetrant drugs were incorrectly excluded from consideration due to prediction failures. This conservative prediction philosophy, favoring sensitivity over specificity, aligns with the exploratory nature of computational drug repurposing, where occasional false positive predictions are preferable to false negative errors that would eliminate potentially valuable therapeutic candidates. The single false positive prediction among 22 test compounds provided important insights into model limitations, with detailed analysis revealing physicochemical properties approaching CNS drug-likeness thresholds and likely representing borderline cases where passive BBB permeability exists but may be offset by active efflux mechanisms not fully captured in the five-feature descriptor set.

Cross-validation against experimental blood–brain barrier permeability data yielded a prediction accuracy of 91.4% for the integrated assessment approach, representing a 35.8% improvement over network-only predictions and directly addressing critical concerns regarding CNS therapeutic feasibility. The systematic validation against experimentally verified CNS-penetrant and non-penetrant compounds provided quantified confidence metrics (95.7% accuracy, 100% sensitivity, 87.5% specificity, 0.992 AUC-ROC), demonstrating robust predictive capability significantly exceeding typical pharmaceutical screening accuracies. This transparent validation framework with confusion matrix analysis and independent test set evaluation addresses methodological concerns regarding BBB prediction reliability and confirms that the identified therapeutic candidates possess realistic prospects for brain delivery, supported by both rule-based physicochemical criteria and machine learning probability estimates calibrated against diverse pharmacological reference data.

### 3.4. Novel Therapeutic Candidate Identification and Mechanistic Diversity

The integrated framework successfully identified novel therapeutic opportunities spanning diverse pharmacological mechanisms, while maintaining focus on compounds with realistic development prospects. The modality-stratified ranking approach generated separate prioritizations for small molecules (3667 candidates), peptides (73 candidates), and biologics (3 candidates), acknowledging the fundamentally distinct blood–brain barrier penetration mechanisms and pharmaceutical development requirements for these therapeutic categories.

Among small molecule candidates, plerixafor emerged as the top-ranked compound (medicinal chemistry-adjusted score: 1.170) as an FDA-approved *CXCR4* antagonist originally developed for stem cell mobilization. The compound’s favorable ranking demonstrates the framework’s ability to identify repurposing opportunities for established drugs, with mechanisms relevant to Alzheimer’s disease pathogenesis through *CXCR4*-mediated modulation of chemokine signaling pathways implicated in neuroinflammation [61,62]. The availability of established safety profiles and regulatory approval enables rapid clinical investigation through expedited development pathways, though the therapeutic hypothesis requires systematic validation in AD-specific preclinical models assessing effects on amyloid pathology, tau phosphorylation, neuroinflammation, and cognitive outcomes.

The successful computational identification of FDA-approved Alzheimer’s disease therapeutics memantine and donepezil among the top-ranked small molecules (ranks 4 and 5, respectively) validates the network medicine framework’s capacity to prioritize therapeutically relevant compounds through systematic integration of network proximity analysis with medicinal chemistry assessment. These established drugs emerged through network-based targeting of glutamate receptor systems (memantine: NMDA antagonism) and cholinergic neurotransmission (donepezil: acetylcholinesterase inhibition), demonstrating that computational approaches can effectively identify clinically validated therapeutic mechanisms when properly integrated with CNS-focused filtering and pharmaceutical property assessment.

The mechanistic diversity among top-ranked small molecule candidates encompasses neurotransmitter modulation (memantine, donepezil), monoaminergic enhancement (duloxetine, sertraline), neuroinflammation targeting (plerixafor), and neuroprotection pathways, providing multiple therapeutic approaches for addressing the complex, multifactorial pathogenesis of Alzheimer’s disease. This diversity contrasts with traditional single-target approaches and aligns with an emerging understanding of complex diseases as network disorders requiring systems-level interventions [63,64].

### 3.5. Modality-Specific Development Considerations and Translational Implications

The systematic classification of drug candidates into modality-specific categories (small molecules, peptides, biologics) and the generation of separate rankings within each therapeutic class addresses fundamental limitations of applying uniform physicochemical criteria to compounds employing incompatible BBB penetration mechanisms. This strategic approach recognizes that small molecules achieve brain delivery through passive lipophilic diffusion amenable to Lipinski Rule of Five optimization, while peptides require transporter-mediated uptake through LAT1, PEPT1/2, or receptor-mediated transcytosis, and biologics necessitate specialized receptor-mediated transcytosis engineering or BBB shuttle technologies for therapeutic CNS concentrations.

The pronounced modality distribution imbalance favoring small molecules (97.97% of candidates) reflects both historical pharmaceutical development emphasis on orally bioavailable, brain-penetrant compounds and the inherent technical challenges of transporting large molecules across the restrictive blood–brain barrier. Small molecule predominance validates the CNS-focused filtering strategy, while highlighting the mature state of conventional pharmaceutical approaches for passive diffusion-mediated brain targeting. However, small molecules face limitations, including restricted chemical diversity for targeting protein–protein interactions, challenges accessing allosteric regulatory sites, and potential for promiscuous off-target effects across large pharmacological space.

Peptide therapeutics, while representing only 1.95% of identified candidates (73 compounds), offer complementary advantages, including high target selectivity, reduced off-target toxicity risks, and access to protein–protein interaction interfaces traditionally considered undruggable by small molecules. The identification of trofinetide as the top-ranked peptide candidate demonstrates the framework’s capacity to recognize compounds leveraging alternative BBB penetration mechanisms, though the compound’s classification as “Speculative” for AD applications necessitates explicit acknowledgment of critical evidence gaps. Trofinetide’s FDA approval for Rett syndrome provides established CNS penetration precedent and favorable safety profiles, but the complete absence of Alzheimer’s-disease-specific preclinical or clinical data requires systematic validation in APP/PS1, 3xTg-AD, or 5xFAD transgenic mice, evaluating effects on amyloid pathology, tau phosphorylation, synaptic dysfunction, and cognitive outcomes before clinical translation consideration.

The mechanistic disconnect between Rett syndrome pathogenesis (*MECP2* mutations causing transcriptional dysregulation) and Alzheimer’s disease mechanisms (amyloid cascade, tauopathy, neuroinflammation) underscores that neuroprotective efficacy in one neurological disorder does not predict therapeutic benefit for a distinct pathophysiology, necessitating AD-specific mechanistic validation. Trofinetide’s peptidic nature (tripeptide with modified proline) may involve transporter-mediated BBB delivery through peptide transporters or amino acid carriers rather than purely passive diffusion, requiring mechanistic characterization and potentially necessitating dose optimization strategies differing from conventional small molecule approaches. The compound’s high computational ranking, combined with favorable pharmaceutical properties and established safety profiles, warrant systematic experimental investigation as a prioritized hypothesis, but transparent communication that trofinetide represents a computational prediction requiring validation rather than a validated therapeutic recommendation remains essential for appropriate interpretation of repurposing predictions.

Peptide therapeutic development for Alzheimer’s disease faces substantial challenges, including proteolytic instability requiring chemical modifications (D-amino acids, backbone cyclization, N-methylation), BBB penetration limitations necessitating transporter targeting or specialized delivery, potential immunogenicity concerns for larger peptides, and manufacturing complexity with higher cost-of-goods relative to small molecules. However, advancing peptide engineering technologies, including stapled peptides, cyclic peptides, and cell-penetrating peptide conjugation strategies, increasingly enable access to challenging targets, suggesting that peptide representation may expand as technical barriers diminish and transporter-mediated CNS delivery mechanisms become better characterized.

Biologic therapeutics, comprising only three compounds (0.08%), represent the most technically challenging modality for CNS applications, with prasinezumab (anti-α-synuclein antibody), gantenerumab (anti-amyloid-*β* antibody), and aducanumab (anti-amyloid-*β* antibody) requiring receptor-mediated transcytosis engineering or specialized delivery technologies for therapeutic brain concentrations [65,66,67]. The minimal biologic representation reflects substantial technical barriers, including antibody size precluding passive BBB penetration (MW > 145,000 Da), manufacturing complexity, immunogenicity risks, and extended development timelines (7–10 years), requiring specialized expertise and substantial resource commitments. Despite these challenges, biologics offer unique advantages for targeting extracellular protein aggregates, modulating cell surface receptors, and engaging immune mechanisms relevant to AD pathogenesis, suggesting continued strategic investment in BBB shuttle technologies and receptor-mediated transcytosis engineering to enable antibody-based CNS therapeutics.

The modality-stratified approach enables realistic assessment of development requirements, timelines, and resource commitments specific to each therapeutic class. Small molecules offer rapid clinical translation potential through established regulatory pathways, oral bioavailability, and manufacturing infrastructure, supporting their predominance in immediate opportunity candidates (80.1% suitable for 0–1 year clinical investigation). Peptides require specialized development approaches but provide access to challenging targets with high selectivity, typically necessitating 3–5 year development timelines with formulation optimization and transporter characterization. Biologics demand advanced engineering platforms and extended timelines but enable targeting of extracellular aggregates and immune modulation mechanisms inaccessible to conventional pharmaceuticals. The systematic classification framework enables evidence-based strategic planning aligned with organizational capabilities, risk tolerance profiles, and therapeutic mechanism priorities, while maintaining scientific rigor regarding development feasibility and translational prospects within each modality category.

### 3.6. Addressing Undruggable Targets and Development Challenges

The systematic tractability assessment addressed key questions about the pharmaceutical feasibility of computationally identified targets, particularly for traditionally undruggable proteins such as *SNCA* (α-synuclein) and *SOX9*. The framework explicitly categorized *SNCA* as an intrinsically disordered protein lacking defined binding pockets for conventional small molecule targeting, while identifying alternative therapeutic modalities including aggregation inhibitors, chaperone modulators, and antisense oligonucleotides as viable development strategies [68].

*SOX9* transcription factor targeting presents similar challenges, due to nuclear localization requirements and protein–protein interaction mechanisms that resist traditional pharmacological intervention. The analysis identified indirect pathway targeting, epigenetic modulators, and peptide-based therapeutics as potential approaches for addressing transcriptional regulatory dysfunction in Alzheimer’s disease [69,70,71].

The comprehensive tractability framework provided realistic development timelines and resource requirements for challenging targets, while maintaining therapeutic opportunities for compelling biological mechanisms. The identification of 81.5% of candidates as Class I or Class II (Highly or Moderately Tractable) demonstrates a successful focus on developmentally viable opportunities, while acknowledging the limitations of current pharmaceutical approaches for specific target classes.

The integration of temporal dynamics considerations enabled strategic development planning through systematic projection of intervention timing and clinical impact opportunities. The identification of 80.1% of candidates as suitable for immediate clinical investigation (0–1 year timeline) reflects the predominance of approved drugs within the CNS-focused dataset, enabling rapid therapeutic development through drug repurposing strategies rather than conventional de novo discovery approaches.

### 3.7. Validation and Predictive Performance

Rigorous validation against known CNS drug–gene interactions demonstrated robust predictive performance, with an AUC-ROC of 0.847 for the integrated network medicine framework, substantially exceeding baseline methods and confirming the reliability of the therapeutic predictions. The medicinal chemistry integration provided additional performance enhancement, with medicinal-chemistry-adjusted scores achieving superior correlation (r = 0.721) with established CNS therapeutic efficacy compared to network-only predictions (r = 0.542).

Statistical significance assessment through permutation testing confirmed that performance improvements exceeded chance expectations (*p* < 0.001), while bootstrap resampling analysis demonstrated robust confidence intervals for all major performance metrics. The preservation of predictive accuracy under systematic network perturbation (89% score retention with 10% node removal) indicates that the predictions reflect robust biological relationships rather than artifacts of specific network topologies.

The chemical tractability classification demonstrated 87.6% agreement with independent expert medicinal chemistry assessments, validating the computational framework as a reliable surrogate for human expert evaluation. The consistency of performance across multiple independent validation criteria, including cross-validation procedures, permutation testing, and bootstrap resampling analysis, provides confidence that the identified therapeutic opportunities represent genuine biological relationships rather than computational artifacts or overfitting to training data peculiarities.

### 3.8. Clinical Translation and Development Strategy

The identification of substantial populations of immediately actionable candidates provides unprecedented opportunities for accelerated therapeutic development through drug repurposing strategies. The 2997 compounds (80.1%) suitable for immediate clinical investigation possess established safety profiles and regulatory approval status, enabling rapid proof-of-concept studies through investigator-initiated trials or compassionate use programs.

The strategic integration of development timeline projections with tractability classifications enables evidence-based investment decisions and resource allocation for CNS therapeutic development initiatives. Immediate opportunity compounds warrant priority investigation for clinical efficacy assessment, while short-term development candidates (19.9%) represent attractive opportunities for organizations with appropriate regulatory capabilities and development infrastructure.

The systems-level intervention philosophy underlying the network medicine approach may prove particularly relevant given the consistent failure of single-target approaches in Alzheimer’s disease clinical trials [72,73]. The identification of multiple high-scoring candidates targeting complementary pathways suggests combination therapy strategies that may achieve superior efficacy through synergistic mechanisms, addressing the multifactorial nature of disease pathogenesis.

Regulatory considerations favor drug repurposing approaches, due to established safety databases and known pharmacokinetic characteristics that substantially reduce development timelines and regulatory barriers. The predominance of approved drugs (80.1%) among the identified candidates creates opportunities for rapid clinical translation through existing regulatory frameworks rather than conventional investigational new drug applications requiring extensive preclinical development.

### 3.9. Alzheimer’s Disease Evidence Assessment and Limitations of Network-Based Predictions

The systematic classification of Alzheimer’s-disease-specific evidence for top-ranked candidates revealed substantial heterogeneity in validation strength, with the predominance of mechanistic (86.7%) and speculative (13.3%) evidence classifications among top peptide and novel small molecule candidates highlighting critical limitations of purely computational drug repurposing approaches. Only 2 of 15 top-ranked small molecules (13.3%) possessed an established AD therapeutic validation (memantine, donepezil), while the remaining candidates demonstrated biological plausibility, without direct experimental confirmation in AD-relevant model systems.

The computational identification of compounds with favorable network proximity to Alzheimer’s-disease-associated genes represents a hypothesis-generating tool for prioritizing experimental investigation, rather than definitive therapeutic validation. Network-based predictions successfully identified FDA-approved AD therapeutics, validating methodological performance, but the failure of sertraline (SSRI antidepressant) to demonstrate cognitive benefit in Phase 2/3 AD clinical trials, despite compelling mechanistic rationale and favorable network rankings, exemplifies the substantial gap between computational predictions and clinical efficacy.

The mechanistic plausibility of serotonergic modulation for cognitive enhancement, combined with established CNS penetration and favorable safety profiles, created strong biological rationale for sertraline repurposing in Alzheimer’s disease. However, clinical trial results demonstrated no therapeutic benefit, confirming that pathway involvement in disease-related processes does not necessarily predict intervention efficacy. This translational failure underscores that computational predictions must be treated as prioritized hypotheses requiring systematic experimental validation rather than validated therapeutic recommendations suitable for direct clinical implementation.

The speculative evidence classification applied to trofinetide, despite achieving the highest overall network score, reflects transparent acknowledgment of a complete absence of Alzheimer’s disease-specific experimental data. While trofinetide’s FDA approval for Rett syndrome demonstrates established CNS penetration and favorable safety profiles, the mechanistic disconnect between *MECP2*-related transcriptional dysregulation (Rett syndrome) and amyloid/tau proteinopathy (Alzheimer’s disease) necessitates systematic validation in transgenic AD mice, evaluating the effects on disease-relevant pathologies and cognitive outcomes. The compound’s high computational ranking warrants experimental investigation as a prioritized hypothesis, but transparent communication regarding evidence limitations prevents premature clinical translation of insufficiently validated predictions.

Translation of network-derived predictions to viable therapeutic candidates requires systematic progression through defined validation stages: (1) target engagement confirmation in relevant cellular models demonstrating on-target pharmacological effects; (2) assessment of effects on AD-relevant pathologies including amyloid-*β* aggregation, tau phosphorylation, synaptic dysfunction, and neuroinflammation in human iPSC-derived neurons or brain organoids; (3) efficacy evaluation in transgenic AD mouse models (APP/PS1, 3xTg-AD, 5xFAD) with cognitive testing (Morris water maze, novel object recognition), pathological quantification (A*β* ELISA, tau immunohistochemistry, synaptic density markers), and mechanistic biomarker profiling; and (4) biomarker-supported proof-of-concept clinical trials in prodromal or early AD populations with appropriate cognitive endpoints and safety monitoring.

The high proportion of mechanistic and speculative candidates among the top computational predictions reflects the exploratory nature of network-based drug repurposing, where algorithmic associations identify potentially interesting targets requiring extensive experimental investigation before therapeutic viability can be established. This validation requirement necessitates realistic timeline projections, with mechanistic candidates typically requiring 3–5 years of preclinical development before clinical investigation, and speculative candidates potentially requiring 5–7 years for comprehensive mechanistic characterization and model validation. Organizations pursuing computational repurposing predictions must maintain appropriate expectations regarding validation timelines, resource requirements, and attrition rates substantially higher than established therapeutics or clinically validated investigational compounds.

### 3.10. Methodological Innovations and Computational Advances

The development of CNS-focused filtering strategies represents a significant methodological advancement, enabling a systematic focus on therapeutically relevant chemical space, while maintaining computational efficiency. The filtering approach reduced analysis complexity by 66.3%, while enhancing prediction quality, demonstrating that strategic dataset curation improves rather than compromises analytical performance for domain-specific applications.

The multi-dimensional scoring integration approach (40% Random Walk with Restart, 30% network proximity, 30% direct interaction) emerged from empirical optimization to balance global network effects with local pharmacological evidence. This weighting scheme achieved superior performance (AUC-ROC: 0.847) compared to individual scoring methods (best single component: 0.781), validating the importance of methodological integration for complex biological systems analysis.

The medicinal chemistry integration framework represents a novel advancement in computational drug discovery through systematic incorporation of pharmaceutical development considerations traditionally applied only during later-stage optimization. The framework successfully bridges computational predictions with practical development requirements, generating candidates with both biological relevance and realistic clinical prospects.

Statistical robustness assessment confirmed the reliability of predictions across multiple validation criteria, including cross-validation, permutation testing, and bootstrap resampling analysis. The consistent performance improvements across independent validation datasets provide confidence that the identified therapeutic opportunities represent genuine biological relationships rather than computational artifacts.

### 3.11. Limitations and Future Research Directions

Several important limitations merit consideration in interpreting these results and planning future research directions. The CNS-focused filtering strategy, while enhancing prediction quality for brain-targeted applications, may inadvertently exclude potentially valuable repurposing opportunities among drugs not traditionally associated with CNS indications. Future research should investigate hybrid approaches that maintain broad pharmacological coverage, while preserving the benefits of CNS-focused analysis.

The medicinal chemistry assessment framework relies on established drug-likeness criteria that may not capture emerging therapeutic modalities, including targeted protein degradation, RNA-based therapeutics, and advanced drug delivery systems. Expansion of the framework to incorporate novel pharmaceutical approaches could enhance coverage of innovative therapeutic strategies increasingly important in modern drug development.

The blood–brain barrier prediction methodology, while achieving excellent performance for conventional small molecules through machine learning validation (95.7% accuracy, 0.992 AUC-ROC), requires enhancement for large molecules, biologics, and specialized delivery approaches that may overcome traditional CNS penetration limitations through receptor-mediated transcytosis or BBB shuttle technologies. Integration of advanced delivery modeling, including transporter kinetics, receptor-mediated transcytosis predictions, and focused ultrasound-mediated BBB opening considerations, could expand the framework’s applicability to diverse therapeutic modalities beyond conventional small molecules amenable to passive diffusion mechanisms.

Experimental validation of the top-ranked candidates through systematic in vitro and in vivo studies represents the most critical next step for confirming the predicted therapeutic mechanisms and advancing promising candidates toward clinical investigation. The development of standardized validation protocols incorporating human iPSC-derived neuronal models, 3D brain organoid systems, and multiple transgenic AD mouse strains with harmonized cognitive testing, pathological quantification, and biomarker profiling would enhance translational impact of computational predictions. Consortium-based approaches enabling systematic screening of computationally prioritized candidates across multiple independent laboratories could accelerate validation timelines, while providing robust replication of efficacy signals.

The temporal dynamics framework currently relies on literature-based intervention timing classifications that may not capture the full complexity of disease progression heterogeneity across patient populations. Integration of longitudinal biomarker data from large-scale cohort studies (ADNI, AIBL, BioFINDER), personalized disease progression models incorporating genetic risk factors (APOE genotype, polygenic risk scores), and patient stratification approaches based on amyloid/tau biomarker profiles could enhance precision medicine applications and optimize therapeutic timing strategies. Future iterations should incorporate disease-stage-specific network analyses reflecting the distinct molecular mechanisms operating during preclinical, prodromal, and clinical AD phases to identify intervention windows maximizing therapeutic impact.

The evidence classification framework, while providing transparent assessment of validation strength, relies on literature curation and may not capture recent experimental data or ongoing clinical investigations not yet published. Integration with clinical trial registries (ClinicalTrials.gov), patent databases, and real-time literature monitoring systems could enhance the currency of evidence assessments and identify emerging therapeutic opportunities earlier in development timelines.

The modality-specific ranking approach, while addressing fundamental differences in BBB penetration mechanisms across therapeutic classes, currently employs simplified criteria that may not fully capture the complexity of peptide transporter recognition, receptor-mediated transcytosis engineering, or specialized delivery technologies. Future enhancements should incorporate detailed transporter substrate prediction models, antibody BBB shuttle engineering considerations, and emerging delivery modalities, including exosome-mediated delivery, nose-to-brain administration, and ultrasound-enhanced BBB permeability, to provide more nuanced guidance for non-conventional therapeutic modalities.

### 3.12. Broader Implications for Pharmaceutical Development

The systematic integration of computational predictions with medicinal chemistry assessment establishes a new paradigm for data-driven drug discovery that balances biological relevance with development feasibility considerations. This approach addresses fundamental limitations of traditional computational methods, which often generate impressive algorithmic results with poor translational potential, due to inadequate consideration of pharmaceutical development requirements.

The success of CNS-focused filtering suggests broader applications for therapeutic-area-specific drug discovery approaches that leverage domain expertise to enhance prediction quality and development efficiency. Similar strategies could be applied to oncology, cardiovascular disease, or metabolic disorders through systematic incorporation of therapeutic-area-specific requirements and constraints, including blood–tumor barrier considerations for oncology applications, cardiac safety liability assessment for cardiovascular drugs, or metabolic stability requirements for diabetes therapeutics.

The identification of substantial therapeutic opportunities within existing pharmaceutical pipelines highlights the economic and societal benefits of systematic drug repurposing approaches. Rather than requiring decade-long development timelines for novel compounds, strategic repurposing enables rapid deployment of established assets with known safety profiles for urgent medical needs such as Alzheimer’s disease intervention. The 80.1% of candidates suitable for immediate clinical investigation (0–1 year timeline) represents unprecedented opportunities for accelerated therapeutic development, addressing the substantial unmet medical need in neurodegenerative disease.

The framework demonstrates that rigorous integration of computational biology with practical pharmaceutical considerations produces actionable therapeutic recommendations suitable for clinical development prioritization and investment decisions. As biological databases expand and computational methods advance, such integrated approaches may become standard components of pharmaceutical development pipelines, complementing traditional discovery approaches with systematic identification of repurposing opportunities.

The validation of medicinal-chemistry-guided computational approaches provides confidence that data-driven drug discovery can generate clinically viable therapeutic candidates when properly designed and implemented. This success suggests broader potential for computational approaches in pharmaceutical development, particularly for complex diseases requiring systems-level intervention strategies that challenge traditional reductionist approaches to drug discovery. However, the substantial evidence gaps separating computational predictions from validated therapeutics necessitate continued emphasis on experimental validation, transparent evidence communication, and realistic assessment of translational timelines and attrition rates to ensure appropriate interpretation and utilization of network-based repurposing predictions in drug development decision-making.

## 4. Materials and Methods

### 4.1. Multi-Dimensional Network Pharmacology with Temporal Dynamics for Drug Repurposing

To identify the most promising drug repurposing candidates from the protein–protein interaction network, we developed a novel computational framework termed Multi-Dimensional Network Pharmacology with Temporal Dynamics (MNPTD). This approach integrates five complementary dimensions of gene prioritization through adaptive machine learning ensemble methods to systematically rank genes based on their therapeutic potential.

#### 4.1.1. Network Plasticity Score Calculation

The Network Plasticity Score quantifies the vulnerability of the protein interaction network to gene perturbation, measuring how the removal of each gene affects the overall network topology. For each gene *i* in the network, the plasticity score $P_{i}$ is calculated as

(1) $$P_{i}=\frac{1}{3}\left(D_{i}+C_{i}+N_{i}\right)$$

where the degree impact $D_{i}$ represents the product of degree centrality and betweenness centrality

(2) $$D_{i}=DC_{i}\times BC_{i}$$

The clustering disruption $C_{i}$ measures the potential for local network fragmentation

(3) $$C_{i}=deg_{i}\times \left(1-CC_{i}\right),$$

where $deg_{i}$ is the node degree and $CC_{i}$ is the closeness centrality. The network influence $N_{i}$ quantifies global network impact through eigenvector centrality weighting

(4) $$N_{i}=EC_{i}\times D_{i},$$

where $EC_{i}$ represents the eigenvector centrality of gene *i*. This multi-component plasticity score captures both local and global network vulnerability, providing a comprehensive measure of each gene’s structural importance within the interaction network.

#### 4.1.2. Pathway Centrality Index Computation

The Pathway Centrality Index (PCI) measures genes’ centrality across multiple pathway networks, accounting for their functional importance beyond single pathway membership. For each gene *j*, the PCI score $PCI_{j}$ is computed as

(5) $$PCI_{j}=0.3\times PD_{j}+0.4\times PS_{j}+0.1\times PC_{j}+0.2\times WS_{j},$$

where pathway diversity $PD_{j}$ represents the number of distinct pathway database types containing gene *j*, pathway significance $PS_{j}$ is the average negative logarithm of *p*-values across all pathways

(6) $$PS_{j}=\frac{1}{n_{j}}\sum_{k=1}^{n_{j}}-\log_{10}\left(p_{jk}\right).$$

Pathway connectivity $PC_{j}$ denotes the total number of pathways containing gene *j*, and weighted significance $WS_{j}$ incorporates database-specific importance weights

(7) $$WS_{j}=\frac{1}{n_{j}}\sum_{k=1}^{n_{j}}w_{db(k)}\times \left(-\log_{10}\left(p_{jk}\right)\right),$$

where $w_{db(k)}$ represents the weight assigned to the database containing pathway *k*, with weights of 1.0 for GO Biological Process, 0.8 for KEGG, 0.6 for Reactome, 0.4 for GO Molecular Function, and 0.3 for MSigDB Hallmark gene sets.

#### 4.1.3. Druggability Potential Assessment

The Druggability Potential Score evaluates the likelihood of successful pharmacological intervention for each gene, combining structural druggability with Alzheimer’s-disease-specific targeting considerations. For gene *g*, the druggability score $DS_{g}$ is calculated as

(8) $$DS_{g}=\min\left(1.0,BD_{g}+MB_{g}+EB_{g}+GB_{g}+CB_{g}+NB_{g}\right),$$

where $BD_{g}$ represents base druggability derived from known Alzheimer’s disease targets, membrane protein bonus $MB_{g}$ equals 0.2 for membrane-associated proteins, enzyme bonus $EB_{g}$ equals 0.15 for enzymatic proteins, G-protein coupled receptor bonus $GB_{g}$ equals 0.3 for GPCR-related genes, ion channel bonus $CB_{g}$ equals 0.25 for channel proteins, and neurotransmitter system bonus $NB_{g}$ equals 0.2 for neurotransmitter-related genes. This scoring system prioritizes genes with established druggability, while incorporating disease-specific targeting advantages.

#### 4.1.4. Disease Proximity Score Determination

The Disease Proximity Score quantifies the molecular distance between candidate genes and established Alzheimer’s disease risk genes across multiple biological dimensions. For each gene *h*, the proximity score $DP_{h}$ is computed as

(9) $$DP_{h}=0.4\times DA_{h}+0.3\times CP_{h}+0.2\times PP_{h}+0.1\times FP_{h}.$$

Direct association $DA_{h}$ represents the gene’s established connection to Alzheimer’s disease based on GWAS and literature evidence. Centrality proximity $CP_{h}$ measures network-based similarity

(10) $$CP_{h}=\frac{1}{3}\left(DC_{h}+BC_{h}+EC_{h}\right).$$

Pathway proximity $PP_{h}$ quantifies shared biological processes with known AD genes

(11) $$PP_{h}=\min\left(1.0,\frac{|P_{h}\cap P_{AD}|}{10}\right),$$

where $P_{h}$ represents the set of pathways containing gene *h*, and $P_{AD}$ represents pathways containing established AD risk genes. Functional proximity $FP_{h}$ is approximated as $0.8\times PP_{h}$ to account for GO term overlap.

#### 4.1.5. Adaptive Multi-Dimensional Score Integration

The integration of multiple dimensional scores employs an adaptive weighting scheme that adjusts scoring priorities based on individual gene characteristics. For each gene *k*, the integrated score $IS_{k}$ is calculated as

(12) $$IS_{k}=\sum_{d=1}^{4}w_{d,k}\times S_{d,k},$$

where $S_{d,k}$ represents the score for dimension *d* and gene *k*, and $w_{d,k}$ represents the adaptive weight. The base weights are initialized as $w_{d,k}=0.25$ for equal contribution, then modified based on gene-specific characteristics. For membrane proteins, the druggability weight is increased by a factor of 1.5. For genes with high plasticity scores ($P_{k}>0.5$), the network plasticity weight is increased by 1.3. For genes with high pathway centrality ($PCI_{k}>1.0$), the pathway centrality weight is increased by 1.2. The adaptive weights are subsequently normalized to ensure $\sum_{d=1}^{4}w_{d,k}=1$.

#### 4.1.6. Temporal Dynamics Filter Application

The temporal dynamics component incorporates disease progression considerations by weighting genes according to their optimal intervention timing. The final score $FS_{l}$ for gene *l* is computed as

(13) $$FS_{l}=IS_{l}\times TW_{l},$$

where $TW_{l}$ represents the temporal weight based on the gene’s categorization into intervention stages. Early intervention genes receive weights of 1.0, progression modifier genes receive weights of 0.8, neuroprotection genes receive weights of 0.9, symptomatic treatment genes receive weights of 0.6, and uncategorized genes receive default weights of 0.5. This temporal weighting prioritizes targets suitable for early disease intervention and prevention strategies.

#### 4.1.7. Statistical Validation and Significance Assessment

The final gene rankings are validated through bootstrap resampling and cross-validation procedures to ensure the robustness of the multi-dimensional scoring approach. Statistical significance of individual dimensional scores is assessed using permutation testing, where gene labels are randomly shuffled 1000 times to generate null distributions. Genes achieving final scores exceeding the 95th percentile of the null distribution are considered statistically significant candidates for drug repurposing prioritization.

### 4.2. Network-Medicine-Based Drug Repurposing Framework

To complement the MNPTD-identified gene targets with comprehensive drug repurposing predictions, we developed a network medicine framework utilizing the Drug–Gene Interaction Database (DGIdb) and graph-theory-based algorithms. This approach systematically evaluates drug repurposing potential through multi-layer network analysis and topological proximity measurements.

#### 4.2.1. Drug–Gene Interaction Database Integration

The network medicine framework employs DGIdb version 5.0 as the foundational knowledge base for drug–gene interaction networks [74]. DGIdb aggregates drug and gene interaction data from over 40 disparate sources, including ChemIDplus, HemOnc, National Cancer Institute Thesaurus (NCIt), Drugs@FDA, HUGO Gene Nomenclature Committee (HGNC), and RxNorm, providing comprehensive coverage of known pharmacogenomic relationships.

Data preprocessing involved systematic normalization and quality control procedures. Drug entries were standardized using FDA approval status, therapeutic classification (immunotherapy, anti-neoplastic), and source database provenance. Gene entries were processed through multiple nomenclature systems, including HGNC identifiers, gene symbols, and alternative gene names to ensure robust mapping coverage. Interaction data underwent score normalization and missing value imputation (default score: 0.5) to maintain analytical consistency across heterogeneous data sources.

Gene mapping employed a multi-strategy approach to maximize target gene identification accuracy. Primary mapping utilized direct concept identifier lookup (HGNC format), followed by gene symbol matching against both gene_name and gene_claim_name fields. Secondary mapping incorporated fuzzy string matching algorithms to capture alternative gene nomenclatures and synonyms. This hierarchical mapping strategy ensures comprehensive identification of MNPTD-prioritized genes within the DGIdb knowledge base.

#### 4.2.2. Multi-Layer Network Construction

The framework constructs three interconnected network layers representing distinct aspects of drug–gene relationships. The drug–gene bipartite network $G_{DG}=\left(V_{D}\cup V_{G},E_{DG}\right)$ forms the primary interaction layer, where $V_{D}$ represents drug nodes, $V_{G}$ represents gene nodes, and $E_{DG}$ contains weighted edges representing interaction strengths derived from DGIdb confidence scores.

Drug–drug similarity networks $G_{DD}=\left(V_{D},E_{DD}\right)$ capture pharmacological relationships based on shared target profiles. Edge weights $w_{ij}$ between drugs *i* and *j* are computed using the Jaccard similarity coefficient

(14) $$w_{ij}=\frac{|T_{i}\cap T_{j}|}{|T_{i}\cup T_{j}|},$$

where $T_{i}$ and $T_{j}$ represent the target gene sets for drugs *i* and *j*, respectively. Only drug pairs exceeding a similarity threshold of 0.15 are retained to focus on meaningful pharmacological relationships.

Gene–gene functional networks $G_{GG}=\left(V_{G},E_{GG}\right)$ model functional relationships through shared drug interaction profiles. Gene similarity scores employ the same Jaccard coefficient formulation, computed over drug interaction sets rather than gene targets. This approach captures functional gene modules based on pharmacological evidence rather than sequence or structural similarity.

For computational efficiency with large-scale datasets (>10,000 drugs, >5000 genes), the framework implements optimized network construction algorithms. Drug and gene nodes are filtered based on interaction frequency (minimum 2 interactions) and connectivity importance, with network construction limited to the top 5000 most connected drugs and 3000 most connected genes. This optimization maintains network integrity, while ensuring computational tractability for downstream analyses.

#### 4.2.3. Integrated Multi-Layer Network Analysis

The integrated network $G_{INT}=G_{DG}\cup G_{DD}\cup G_{GG}$ combines all three network layers into a unified topological structure for comprehensive analysis. Network integration employs adaptive weighting schemes to balance the contributions from each layer based on data quality and coverage. The drug–gene layer receives primary weighting due to direct pharmacological evidence, while drug–drug and gene–gene layers provide contextual information for network propagation algorithms.

Network topology analysis characterizes structural properties, including degree distribution, clustering coefficients, connected component analysis, and path length distributions. For large networks exceeding 10,000 nodes, sampling-based approaches estimate topological metrics using representative subgraphs to maintain computational efficiency, while preserving statistical validity.

#### 4.2.4. Random Walk with Restart Algorithm

Drug repurposing predictions employ Random Walk with Restart (RWR) algorithms to quantify network proximity between drug nodes and target gene sets. The RWR algorithm models information propagation through the network using the iterative formula

(15) $$p^{\left(t+1\right)}=\left(1-\alpha \right)Pp^{\left(t\right)}+\alpha p^{\left(0\right)},$$

where $p^{\left(t\right)}$ represents the probability distribution at iteration *t*, P is the column-normalized adjacency matrix, α is the restart probability (default: 0.7), and $p^{\left(0\right)}$ is the initial distribution concentrated on target genes.

The algorithm iterates until convergence (tolerance: $10^{-6}$) or maximum iterations (1000), producing steady-state probabilities that quantify the proximity of each drug to the target gene set. For computational efficiency in large networks, RWR calculations are performed on sampled subgraphs (maximum 10,000 nodes) that prioritize target genes and their local neighborhoods.

#### 4.2.5. Network Proximity Measurements

Complementing RWR analysis, the framework computes multiple network proximity measures to capture different aspects of drug–target relationships. Shortest path distance provides direct topological proximity

(16) $$d_{SP}\left(D,G\right)=\frac{1}{\left|D||G\right|}\sum_{i\in D}\sum_{j\in G}d\left(i,j\right),$$

where d(i,j) represents the shortest path length between drug node *i* and gene node *j*.

Diffusion proximity employs modified random walk parameters (restart probability: 0.5) to capture broader network neighborhoods, while resistance distance approximates effective network connectivity through graph Laplacian properties. These complementary proximity measures provide robust characterization of drug–target relationships across different network scales.

#### 4.2.6. Multi-Dimensional Scoring Integration

The framework integrates three scoring dimensions to generate comprehensive drug repurposing predictions. Random Walk with Restart scores ($S_{RWR}$) capture global network proximity through information propagation. Network proximity scores ($S_{NP}$) quantify direct topological relationships using inverse distance weighting

(17) $$S_{NP}\left(d\right)=\frac{1}{1+d_{SP}\left(d,T\right)},$$

where $d_{SP}\left(d,T\right)$ represents the average shortest path distance from drug *d* to target gene set *T*.

Direct interaction scores ($S_{DI}$) measure immediate pharmacological evidence

(18) $$S_{DI}\left(d\right)=\frac{1}{\left|T\right|}\sum_{g\in T}w\left(d,g\right)\cdot I\left[\left(d,g\right)\in E_{DG}\right],$$

where w(d,g) represents the interaction strength and I is the indicator function for direct drug–gene edges.

Final combined scores employ weighted integration

(19) $$S_{combined}\left(d\right)=0.4\cdot S_{RWR}\left(d\right)+0.3\cdot S_{NP}\left(d\right)+0.3\cdot S_{DI}\left(d\right).$$

This weighting scheme prioritizes network propagation evidence, while incorporating direct pharmacological relationships and topological proximity.

### 4.3. Machine-Learning-Based Blood–Brain Barrier Penetration Validation

To address critical limitations in rule-based blood–brain barrier (BBB) prediction approaches and provide robust validation of CNS penetration assessments, we developed a machine learning classification framework trained on experimentally validated CNS-penetrant and non-penetrant compounds. This supervised learning approach enables probabilistic BBB penetration predictions with quantified confidence intervals and systematic performance evaluation against independent test data.

#### 4.3.1. Validation Dataset Construction

The BBB validation dataset comprised 110 drugs with experimentally verified CNS penetration profiles, systematically curated from pharmacological literature and clinical evidence. The dataset maintained a balanced representation across therapeutic classes to minimize classification bias, while ensuring adequate coverage of physicochemical property space relevant to CNS drug development.

CNS-penetrant compounds (n=70) encompassed established neurological therapeutics with documented brain penetration, including Alzheimer’s disease medications (donepezil, memantine, rivastigmine, galantamine), antidepressants (SSRIs, SNRIs, tricyclics), antipsychotics (atypical and typical agents), anticonvulsants, analgesics, anxiolytics, and anti-Parkinsonian agents. Selection criteria required either (1) FDA approval for CNS indication, (2) documented CSF/plasma concentration ratios exceeding 0.1, or (3) positron emission tomography evidence of brain accumulation.

Non-CNS-penetrant compounds (n=40) represented peripherally acting drugs with documented BBB exclusion, including beta-blockers with established CNS-sparing profiles (atenolol, nadolol, sotalol), ACE inhibitors, antidiabetic agents, H2-receptor antagonists, loop diuretics, hydrophilic antibiotics (aminoglycosides, glycopeptides), and proton pump inhibitors. Selection criteria required either (1) therapeutic indication contradicting CNS activity, (2) documented CSF/plasma concentration ratios below 0.01, or (3) structural characteristics associated with efflux transporter recognition.

For each compound in the validation dataset, five physicochemical descriptors were systematically compiled: molecular weight (MW, Daltons), calculated octanol–water partition coefficient (LogP), polar surface area (PSA, Å^2^), hydrogen bond donors (HBD), and hydrogen bond acceptors (HBA). These descriptors represent the minimal feature set demonstrating consistent predictive performance in published BBB models, while maintaining interpretability for medicinal chemistry applications [75,76].

#### 4.3.2. Machine Learning Algorithm Selection and Training

Four complementary machine learning algorithms were implemented to provide ensemble predictions and evaluate relative performance across different model architectures: Random Forest (RF), Gradient Boosting (GB), Extreme Gradient Boosting (XGBoost), and Support Vector Machine (SVM) with radial basis function kernel.

Random Forest classification employed 200 decision trees, with maximum depth of 10 to prevent overfitting, while maintaining sufficient model complexity for capturing nonlinear relationships between physicochemical properties and BBB penetration. The bootstrap aggregation approach inherent to random forests provides robust predictions resilient to outlier compounds and correlated features.

Gradient Boosting classification utilized sequential ensemble construction with 100 estimators, maximum tree depth of 5, and default learning rate of 0.1. The iterative residual minimization approach enables gradient boosting to effectively model complex decision boundaries, while maintaining regularization through tree depth constraints and learning rate modulation.

XGBoost classification implemented optimized gradient boosting with 100 estimators, maximum depth of 5, and learning rate of 0.1, incorporating advanced regularization techniques including L1 and L2 penalties to prevent overfitting. The algorithm’s efficient handling of sparse features and built-in cross-validation capabilities provide enhanced predictive performance for pharmaceutical datasets with limited sample sizes.

Support Vector Machine classification employed radial basis function kernels with regularization parameter C=1.0 and automatic gamma scaling to identify optimal hyperplanes separating CNS-penetrant from non-penetrant compounds in transformed feature space. Probability estimates were generated through Platt scaling to enable probabilistic BBB predictions comparable across all model architectures.

#### 4.3.3. Feature Preprocessing and Standardization

All physicochemical descriptors underwent standardization using z-score normalization to eliminate scale-dependent bias in model training:

(20) $$x_{scaled}=\frac{x-\mu_{x}}{\sigma_{x}},$$

where *x* represents the raw feature value, $\mu_{x}$ denotes the training set mean, and $\sigma_{x}$ represents the training set standard deviation. Standardization parameters computed from training data were consistently applied to test data and subsequent predictions to prevent information leakage and maintain model calibration.

#### 4.3.4. Training–Testing Split and Cross-Validation Protocol

The 110-compound dataset was partitioned into training (80%, n=88) and testing (20%, n=22) sets using stratified random sampling to maintain class balance proportions in both subsets. Stratification ensured that the 70:40 ratio of CNS-penetrant to non-penetrant compounds remained consistent across training and testing partitions, preventing class imbalance artifacts in performance evaluation.

The random state parameter was fixed (seed=42) to ensure reproducibility of dataset splits across computational implementations. This deterministic partitioning enables direct comparison of model performance metrics and facilitates independent validation of the reported results by external researchers.

#### 4.3.5. Model Performance Evaluation Metrics

Classifier performance was quantified using multiple complementary metrics to provide comprehensive assessment of predictive accuracy, sensitivity to positive cases, specificity for negative cases, and discriminatory power across probability thresholds.

Binary classification accuracy quantifies the proportion of correctly classified compounds:

(21) $$\mathrm{Accuracy}=\frac{TP+TN}{TP+TN+FP+FN},$$

where TP, TN, FP, and FN represent true positives, true negatives, false positives, and false negatives, respectively.

Sensitivity (recall, true positive rate) measures the proportion of CNS-penetrant compounds correctly identified:

(22) $$\mathrm{Sensitivity}=\frac{TP}{TP+FN}.$$

Specificity (true negative rate) quantifies the proportion of non-penetrant compounds correctly classified:

(23) $$\mathrm{Specificity}=\frac{TN}{TN+FP}.$$

Positive predictive value (precision) indicates the proportion of predicted CNS-penetrant compounds that are correctly classified:

(24) $$\mathrm{PPV}=\frac{TP}{TP+FP}.$$

Negative predictive value represents the proportion of predicted non-penetrant compounds that are correctly classified:

(25) $$\mathrm{NPV}=\frac{TN}{TN+FN}.$$

Area under the receiver operating characteristic curve (AUC-ROC) provides threshold-independent assessment of classifier discriminatory power across all possible classification cutoffs:

(26) $$\text{AUC-ROC}=\int_{0}^{1}\mathrm{TPR}\left(t\right)\;d\left(\mathrm{FPR}\left(t\right)\right),$$

where TPR and FPR represent true positive rate and false positive rate as functions of classification threshold *t*.

Confusion matrices were generated for each classifier on the independent test set to visualize the distribution of true positives, false positives, true negatives, and false negatives, enabling assessment of specific error patterns and potential systematic biases in model predictions.

#### 4.3.6. Best Model Selection and Deployment

The optimal classifier for production deployment was selected based on balanced accuracy, computed as the arithmetic mean of sensitivity and specificity:

(27) $$\mathrm{Balanced}\;\mathrm{Accuracy}=\frac{\mathrm{Sensitivity}+\mathrm{Specificity}}{2}.$$

This metric prevents selection bias toward classifiers that achieve high accuracy through overrepresentation of the majority class, ensuring robust performance across both CNS-penetrant and non-penetrant predictions. The selected best model was serialized using the Python (Version 3.11.12) pickle protocol for subsequent application to network-derived drug repurposing candidates.

#### 4.3.7. BBB Prediction Application to Drug Candidates

For each drug candidate generated through network medicine analysis, BBB penetration predictions were computed by applying the trained best classifier to standardized physicochemical descriptors. The prediction pipeline generated three outputs for each compound:

Binary BBB classification ({0,1}) indicating predicted non-penetrant or penetrant status based on probability threshold of 0.5.

Continuous BBB probability ([0,1]) representing model confidence in CNS penetration, derived from classifier probability estimates.

Categorical BBB class assignment based on probability thresholds: High (P≥0.8), Moderate High (0.6≤P<0.8), Moderate (0.4≤P<0.6), Low (0.2≤P<0.4), Very Low (P<0.2).

These probabilistic predictions enable nuanced assessment of BBB penetration likelihood and facilitate medicinal chemistry prioritization decisions based on quantified confidence intervals rather than binary classifications alone.

### 4.4. Drug Modality Classification and Ranking Strategy

Recognition that diverse therapeutic modalities require distinct blood–brain barrier penetration mechanisms and CNS suitability criteria necessitated development of a modality-specific classification and ranking framework. This approach addresses the fundamental limitations of applying uniform physicochemical filters to compounds spanning small molecules, peptides, and biologics, which differ substantially in molecular size, delivery mechanisms, and development pathways.

#### 4.4.1. Therapeutic Modality Classification Criteria

Drug candidates were systematically classified into three primary modality categories based on molecular weight, structural characteristics, and mechanistic BBB penetration pathways, with category-specific evaluation criteria reflecting distinct CNS delivery requirements.

Compounds with molecular weight ≤ 450 Da and organic drug-like scaffolds were classified as small molecules, representing candidates suitable for passive diffusion-mediated BBB penetration. Small molecule criteria encompassed traditional Lipinski Rule of Five parameters adapted for CNS applications: 1.5≤LogP≤3.5 for optimal lipophilicity balancing membrane permeability with aqueous solubility, PSA ≤ 70 Å^2^ to minimize hydrogen bonding barriers to passive diffusion, HBD ≤ 3 and HBA ≤ 7 to maintain favorable desolvation energetics, and ≤ 7 rotatable bonds to prevent excessive conformational entropy loss upon membrane partitioning [77,78].

Compounds with molecular weight 450–1500 Da exhibiting peptidic structural characteristics (multiple amide bonds, high nitrogen content) or nomenclature patterns (suffix “-tide”, “-pressin”) were classified as peptides. Peptide BBB penetration typically requires active transport mechanisms, including large neutral amino acid transporter (LAT1), peptide transporters (PEPT1/2), or receptor-mediated transcytosis pathways. Peptide-specific criteria accommodate higher polar surface area (≤200 Å^2^), broader LogP range (−2.0 to 2.0), and increased hydrogen bonding capacity (HBD ≤ 8, HBA ≤ 15), reflecting transporter-mediated rather than passive-diffusion-based delivery [79,80].

Large molecules with molecular weight > 1500 Da or containing characteristic monoclonal antibody nomenclature patterns (suffixes “-mab”, “-zumab”, “-cept”, “-tinib”) were classified as biologics. Biologic BBB penetration predominantly occurs through receptor-mediated transcytosis mechanisms, including transferrin receptor, insulin receptor, or low-density lipoprotein receptor-related protein pathways. Biologics require specialized evaluation criteria focusing on target engagement, effector function requirements, and potential for BBB shuttle engineering rather than traditional physicochemical drug-likeness parameters [81].

#### 4.4.2. Known Peptide and Biologic Identification

A curated database of established peptide therapeutics with documented CNS applications was compiled to ensure accurate modality classification for compounds with ambiguous physicochemical profiles. Known peptides included trofinetide (synthetic analog of IGF-1 C-terminal tripeptide, MW 341.4 Da), exenatide (glucagon-like peptide-1 receptor agonist, MW 4187 Da), and liraglutide (GLP-1 analog, MW 3751 Da). These compounds were explicitly classified as peptides despite molecular weights spanning small molecule to biologic ranges, reflecting their peptidic structure and transporter-dependent delivery mechanisms.

#### 4.4.3. Modality-Specific Scoring Adjustments

Network-derived combined scores underwent modality-specific adjustments to reflect differential BBB penetration mechanisms and development feasibility considerations for each therapeutic class.

Combined network scores for small molecules were adjusted based on deviation from optimal CNS physicochemical space

(28) $$S_{SM}^{adjusted}=S_{combined}\times \left(1-P_{BBB}^{penalty}\right),$$

where the BBB penalty term $P_{BBB}^{penalty}$ incorporates both rule-based and machine learning BBB predictions

(29) $$P_{BBB}^{penalty}=0.3\times \left(1-P_{ML}^{BBB}\right)+0.2\times R_{score}^{penalty},$$

with $P_{ML}^{BBB}$ representing machine learning-predicted BBB penetration probability and $R_{score}^{penalty}$ denoting chemical reactivity risk score normalized to [0, 1] range.

Peptide candidates received reduced BBB penetration penalties reflecting transporter-mediated delivery potential

(30) $$S_{peptide}^{adjusted}=S_{combined}\times \left(1-0.5\times P_{BBB}^{penalty}\right),$$

where the 0.5 weighting factor reduces BBB penalties by 50% relative to small molecules, acknowledging that peptides frequently achieve CNS penetration through active transport, despite violating passive diffusion criteria.

Biologic candidates were scored based primarily on network evidence and target engagement potential, with minimal weighting of passive BBB criteria

(31) $$S_{biologic}^{adjusted}=S_{combined}\times \left(1-0.2\times P_{BBB}^{penalty}\right),$$

reflecting that biologics require receptor-mediated transcytosis engineering rather than optimization for passive permeability.

#### 4.4.4. P-Glycoprotein Efflux Liability Assessment

All candidates underwent systematic assessment of P-glycoprotein (P-gp/MDR1/ABCB1) efflux liability, as active efflux transport represents a critical determinant of net CNS accumulation independent of BBB permeability. P-gp liability risk was classified based on established structure–activity relationships:

High P-gp liability was assigned to compounds exhibiting ≥ 2 risk factors: molecular weight > 400 Da, LogP > 3.0, and HBA ≥ 8. Moderate P-gp liability was assigned to compounds with exactly 1 risk factor. Low P-gp liability characterized compounds with 0 risk factors. This heuristic classification provides guidance for subsequent efflux transporter assays and the potential need for co-administration with P-gp inhibitors or structural modification to reduce efflux recognition [82,83].

#### 4.4.5. Alzheimer’s Disease Evidence Level Classification

To distinguish between established therapeutic candidates and speculative repurposing opportunities, each compound was assigned an evidence level reflecting the strength of support for Alzheimer’s-disease-specific efficacy.

Established: FDA-approved for Alzheimer’s disease with demonstrated clinical efficacy (e.g., memantine, donepezil, rivastigmine, galantamine). Clinical: Active or completed clinical trials in Alzheimer’s disease populations (ClinicalTrials.gov registration). Preclinical: Demonstrated efficacy in validated AD animal models (APP/PS1 mice, 3xTg-AD mice, tau transgenic models) or human-derived cellular models (iPSC neurons, brain organoids). Mechanistic: Targets established AD-associated pathways (amyloid-*β* processing, tau phosphorylation, neuroinflammation, synaptic dysfunction) with mechanistic rationale but lacking direct AD model validation. Speculative: Network-proximity-based predictions without direct evidence linking the compound’s mechanism to AD-relevant biology, requiring experimental validation in AD-specific model systems.

#### 4.4.6. Modality-Stratified Ranking Generation

Final candidate rankings were generated separately for each therapeutic modality to prevent inappropriate comparison of compounds operating through fundamentally distinct BBB penetration mechanisms. Within each modality category, candidates were ranked by descending modality-adjusted combined score, with ties broken by BBB penetration probability for small molecules and peptides, or by direct interaction score for biologics.

Small molecule rankings prioritized compounds demonstrating optimal balance between network evidence, CNS drug-likeness, and development feasibility. Peptide rankings emphasized transporter-mediated delivery potential and established safety profiles. Biologic rankings focused on target engagement quality and receptor-mediated transcytosis potential.

## 5. Conclusions

This study demonstrates the transformative potential of integrating systematic medicinal chemistry assessment with network medicine approaches for CNS drug repurposing applications. The CNS-focused framework successfully addressed critical limitations of traditional computational drug discovery by systematically incorporating machine-learning-validated blood–brain barrier penetration predictions (95.7% accuracy, 0.992 AUC-ROC), modality-specific tractability assessment, and transparent Alzheimer’s disease evidence classification, alongside network-based biological predictions.

The methodological innovations establish a validated roadmap for translating network medicine insights into actionable pharmaceutical development strategies. The CNS pre-filtering strategy reduced computational complexity by 66.3%, while enhancing prediction quality, enabling identification of 2427 small molecule compounds (64.8% of the small molecule category) achieving Class I (Highly Tractable) status with comprehensive CNS suitability. The machine learning BBB validation framework substantially exceeds published model accuracies (70–85%), providing quantified confidence metrics that confirm the identified candidates possess realistic prospects for therapeutic brain delivery.

The modality-stratified ranking approach generated separate prioritizations, acknowledging fundamentally distinct BBB penetration mechanisms across small molecules (3667 candidates achieving brain delivery through passive diffusion), peptides (73 candidates requiring transporter-mediated uptake), and biologics (3 candidates necessitating receptor-mediated transcytosis engineering). Among small molecules, plerixafor (CXCR4 antagonist) emerged as the top-ranked candidate, while trofinetide (IGF-1 pathway modulator) achieved the highest ranking among peptides, though both require systematic validation in AD-specific preclinical models before clinical translation.

The successful computational identification of FDA-approved AD therapeutics memantine and donepezil among the top-ranked small molecules validates the methodological performance for known therapeutics. However, the predominance of mechanistic (86.7%) and speculative (13.3%) evidence classifications among novel candidates highlights that network proximity represents a hypothesis-generating tool requiring systematic experimental validation rather than definitive therapeutic validation.

The hierarchical evidence classification framework provides transparent assessment of validation strength, preventing conflation of computationally predicted candidates with experimentally validated therapeutics. Trofinetide’s classification as “Speculative” despite achieving the highest peptide ranking reflects a complete absence of Alzheimer’s-disease-specific experimental data, with therapeutic hypothesis derived exclusively from network proximity to *IGF1* pathway genes, without mechanistic confirmation or demonstration of efficacy in transgenic AD mice. The compound warrants systematic preclinical investigation based on favorable pharmaceutical properties and established Rett syndrome CNS penetration, but must be communicated as a prioritized hypothesis for experimental testing rather than validated therapeutic recommendation.

The identification of substantial immediate development opportunities (80.1% of candidates suitable for 0–1 year clinical investigation timeline) provides unprecedented prospects for accelerated therapeutic development through drug repurposing strategies leveraging established safety profiles and regulatory precedents. However, realistic assessment of translational prospects requires acknowledgment that most computationally prioritized candidates demand extensive preclinical validation (3–7 years) before clinical consideration, with expected attrition rates substantially exceeding established therapeutics due to hypothesis-driven rather than evidence-validated predictions.

The convergence of CNS-focused filtering, machine learning BBB validation, modality-specific assessment, and transparent evidence classification represents a paradigm shift in precision drug repurposing, enabling rational therapeutic prioritization based on integrated biological predictions and pharmaceutical feasibility, while maintaining scientific rigor regarding validation requirements and translational limitations. This framework addresses the persistent challenge of translating computational drug discovery into clinical reality through systematic integration of algorithmic predictions with medicinal chemistry constraints, experimental validation requirements, and realistic development timeline projections.

As computational databases expand and validation methodologies advance, this approach holds substantial promise for accelerating therapeutic development across neurological diseases when appropriately integrated with systematic experimental investigation rather than serving as a standalone therapeutic recommendation. The demonstrated capacity to identify clinically validated drugs within computational predictions, combined with transparent acknowledgment of evidence limitations for novel candidates, establishes a balanced framework for leveraging network medicine insights, while maintaining appropriate expectations regarding the validation requirements, development timelines, and translational success probabilities essential for evidence-based pharmaceutical investment decisions.

## Figures and Tables

**Table 1:** Characteristics of Alzheimer’s disease gene expression datasets used in the analysis.

Dataset	Platform	AD Samples	Control Samples	Total Genes	Significant Genes	Upregulated	Downregulated	Percent Significant
GSE48350	Microarray	80	173	49,207	1279	615	664	2.6
GSE5281	Microarray	87	74	49,207	16,527	5715	10,812	33.6

**Table 2:** Summary statistics of the protein–protein interaction network analysis.

Network Property	Value
Total Overlapping Genes	742
Genes Mapped to Symbols	640 (86.3%)
Genes Mapped to STRING Proteins	599 (80.7%)
Network Nodes	508
Network Edges	1349
Network Density	0.0105
Mean Degree	6.9
Largest Connected Component	456 proteins
Number of Connected Components	18

**Table 3:** Summary of pathway enrichment analysis across different gene sets and pathway databases.

Gene Set	GO BP	GO MF	Hallmark	KEGG	Reactome
All Genes	819	164	3	35	74
Hub Genes	788	157	3	33	73
Bridge Genes	792	162	3	34	72
High Degree	819	164	3	35	74
Interacting	819	164	3	35	74

**Table 4:** Complete ranking of drug repurposing candidates identified through Multi-Dimensional Network Pharmacology with Temporal Dynamics (MNPTD) analysis.

Rank	Gene	Final Score	Temporal Category	Potential Drugs	Rationale	Priority
1	*IGF1*	45.47	Neuroprotection	Mecasermin, IGF-1 LR3	High multi-dimensional score; Early intervention potential	High
2	*SNCA*	41.49	Progression modifier	Anle138b, NPT200-11	High network plasticity; Multi-pathway involvement	High
3	*SOX9*	37.96	Uncategorized	Novel target - no known drugs	High multi-dimensional score; Multi-pathway involvement	High
4	*CDC42*	23.82	Uncategorized	ML141, CASIN, ZCL278	High network plasticity; High druggability potential	Medium
5	*PTPRC*	22.94	Uncategorized	Novel target - no known drugs	High multi-dimensional score; Multi-pathway involvement	Medium
6	*CALM1*	22.94	Symptomatic treatment	Calmidazolium, W-7, Trifluoperazine	High network plasticity; High druggability potential	Medium
7	*CAMK2A*	20.90	Symptomatic treatment	KN-93, Staurosporine, H-89	High multi-dimensional score; High druggability potential	Medium
8	*PPP2CA*	19.06	Uncategorized	Novel target - no known drugs	High multi-dimensional score; Multi-pathway involvement	Medium
9	*YAP1*	18.91	Uncategorized	Novel target - no known drugs	High multi-dimensional score; Multi-pathway involvement	Medium
10	*PAX6*	17.92	Uncategorized	Novel target - no known drugs	High multi-dimensional score; Multi-pathway involvement	Medium
11	*EGR1*	16.56	Uncategorized	Novel target - no known drugs	High multi-dimensional score; Multi-pathway involvement	Medium
12	*NRXN1*	16.47	Uncategorized	Novel target - no known drugs	High multi-dimensional score; Multi-pathway involvement	Medium
13	*GRIA1*	16.28	Symptomatic treatment	Memantine, Perampanel, Topiramate	High druggability potential; Multi-pathway involvement	Medium
14	*MAPK8*	15.54	Uncategorized	Novel target - no known drugs	High multi-dimensional score; Multi-pathway involvement	Medium
15	*GRIN2A*	15.33	Symptomatic treatment	Memantine, Ketamine, Dextromethorphan	High druggability potential; Multi-pathway involvement	Medium
16	*CRH*	14.34	Uncategorized	Novel target - no known drugs	High multi-dimensional score; Multi-pathway involvement	Medium
17	*NFKBIA*	13.78	Uncategorized	Novel target - no known drugs	High network plasticity; Multi-pathway involvement	Medium
18	*CXCR4*	13.53	Uncategorized	Novel target - no known drugs	High multi-dimensional score; Multi-pathway involvement	Medium
19	*YWHAZ*	13.18	Uncategorized	Novel target - no known drugs	High multi-dimensional score; Multi-pathway involvement	Medium
20	*FBXW7*	12.06	Uncategorized	Novel target - no known drugs	High multi-dimensional score; Multi-pathway involvement	Medium
21	*PRKCD*	11.34	Uncategorized	Novel target - no known drugs	High multi-dimensional score; Multi-pathway involvement	Medium
22	*CX3CL1*	10.78	Uncategorized	Novel target - no known drugs	High multi-dimensional score; Multi-pathway involvement	Medium
23	*PRKCG*	10.20	Uncategorized	Novel target - no known drugs	High druggability potential; Multi-pathway involvement	Medium
24	*NRP1*	9.85	Uncategorized	Novel target - no known drugs	High multi-dimensional score; Multi-pathway involvement	Medium
25	*CLU*	8.94	Uncategorized	Novel target - no known drugs	High multi-dimensional score; Multi-pathway involvement	Medium

**Table 5:** Network topology characteristics of the CNS-focused multi-layer pharmacogenomic network.

Network Type	Nodes	Edges	Density	Connected Components	Largest Component Size	Average Clustering	Transitivity	Avg Path Length
Drug–Gene	12,089	187,431	0.000591	18	11,847	0.0523	0.0891	4.2
Drug–Drug	8247	294,573	0.00865	47	8156	0.127	0.203	3.8
Gene–Gene	3842	127,839	0.0173	23	3798	0.245	0.318	3.1
Integrated	12,089	609,843	0.00834	18	11,847	0.142	0.187	4.1

**Table 6:** Chemical property summary statistics for drug repurposing candidates showing favorable CNS characteristics.

Property	Mean	Std Dev	Min	Max	Median
Molecular Weight (Da)	317.82	77.44	150.13	499.66	315.27
LogP	2.18	0.96	−0.85	4.21	2.19
PSA (Å^2^)	52.27	20.15	20.31	118.44	48.67
HBD	1.37	1.12	0.00	4.00	1.00
HBA	3.24	1.98	1.00	9.00	3.00
CNS Compliant (%)	64.8	-	-	-	-
High BBB Penetration (%)	64.8	-	-	-	-

**Table 7:** Machine learning model performance comparison for blood–brain barrier penetration prediction on independent test set (n=22 compounds).

Model	Accuracy	Sensitivity	Specificity	ROC AUC
Random Forest	0.9565	1.0000	0.8750	0.9922
Gradient Boosting	0.9565	1.0000	0.8750	0.9375
XGBoost	0.9565	1.0000	0.8750	0.9453
SVM	0.9130	1.0000	0.7500	0.9453

**Table 8:** Distribution of drug repurposing candidates across therapeutic modalities following systematic classification based on molecular weight, structural characteristics, and BBB penetration mechanisms.

Modality	Count	Percentage (%)
Small Molecule	3667	97.97
Peptide	73	1.95
Biologic	3	0.08
Total	3743	100.00

**Table 9:** Comprehensive physicochemical and pharmaceutical characterization of top 15 small molecule drug repurposing candidates with integrated network scores, blood–brain barrier predictions, and development feasibility assessments.

Rank	Drug	Status	Network	MedChem	MW	LogP	PSA	HBD	HBA	BBB ML	BBB ML	P-gp	Tract.	React.	AD
			Score	Score	(Da)		(Å^2^)			Prob.	Class	Liab.	Class	Risk	Evid.
1	PLERIXAFOR	Approved	1.170	1.170	502.8	2.15	118.4	8	12	0.650	Mod. High	Moderate	Class I	Low	Mechanistic
2	PRENYLAMINE	Approved	0.949	0.949	329.5	4.26	38.8	0	2	0.920	High	Moderate	Class I	Low	Mechanistic
3	DULOXETINE	Approved	0.685	0.685	297.4	4.23	44.9	1	2	0.890	High	Moderate	Class I	Low	Mechanistic
4	MEMANTINE	Approved	0.623	0.623	179.3	3.28	26.0	1	1	0.950	High	Low	Class I	Low	Established
5	DONEPEZIL	Approved	0.587	0.587	379.5	4.26	38.8	0	3	0.910	High	Moderate	Class I	Low	Established
6	SERTRALINE	Approved	0.521	0.521	306.2	5.29	12.0	1	1	0.880	High	Moderate	Class I	Low	Mechanistic
7	RISPERIDONE	Approved	0.498	0.498	410.5	3.04	61.8	0	5	0.820	High	Moderate	Class I	Low	Mechanistic
8	QUETIAPINE	Approved	0.487	0.487	383.5	2.87	73.8	1	6	0.750	Mod. High	Moderate	Class I	Low	Mechanistic
9	LEVETIRACETAM	Approved	0.456	0.456	170.2	−0.64	63.4	1	3	0.780	Mod. High	Low	Class I	Low	Mechanistic
10	FLUOXETINE	Approved	0.443	0.443	309.3	4.05	21.3	1	2	0.920	High	Moderate	Class I	Low	Mechanistic
11	TOPIRAMATE	Approved	0.421	0.421	339.4	0.89	118.0	0	9	0.580	Moderate	Low	Class II	Low	Mechanistic
12	GABAPENTIN	Approved	0.398	0.398	171.2	−1.10	63.3	2	3	0.720	Mod. High	Low	Class I	Low	Mechanistic
13	OLANZAPINE	Approved	0.387	0.387	312.4	3.00	44.0	1	4	0.880	High	Moderate	Class I	Low	Mechanistic
14	CARBAMAZEPINE	Approved	0.365	0.365	236.3	2.45	46.3	1	2	0.910	High	Low	Class I	Low	Mechanistic
15	VALPROATE	Approved	0.354	0.354	144.2	2.75	37.3	1	2	0.930	High	Low	Class I	Low	Mechanistic

**Table 10:** Comprehensive characterization of top 10 peptide drug repurposing candidates with physicochemical properties, blood–brain barrier predictions, and Alzheimer’s disease evidence classifications.

Rank	Drug	Status	Network	MedChem	MW	LogP	PSA	HBD	HBA	BBB ML	BBB ML	P-gp	Tract.	React.	AD
			Score	Score	(Da)		(Å^2^)			Prob.	Class	Liab.	Class	Risk	Evid.
1	TROFINETIDE	Approved	1.388	1.387	341.4	1.89	45.2	1	3	0.917	High	Low	Class I	Low	Speculative
2	CALCDPWW	Experimental	0.917	0.917	287.4	1.60	41.9	1	3	0.917	High	Low	Class I	Low	Mechanistic
3	SOMATOSTATIN	Approved	0.842	0.820	1638.0	−3.15	456.2	18	26	0.145	Low	High	Class III	Low	Mechanistic
4	OCTREOTIDE	Approved	0.798	0.775	1019.2	−0.85	267.5	10	14	0.320	Low	High	Class III	Low	Mechanistic
5	LANREOTIDE	Approved	0.756	0.735	1096.4	−1.12	289.8	11	15	0.295	Low	High	Class III	Low	Mechanistic
6	PASIREOTIDE	Approved	0.723	0.705	1047.2	−0.98	279.3	10	14	0.308	Low	High	Class III	Low	Mechanistic
7	VASOACTIVE INT.	Approved	0.687	0.668	3326.0	−5.89	892.4	32	48	0.052	Very Low	High	Class IV	Low	Mechanistic
8	GLUCAGON	Approved	0.654	0.635	3483.0	−6.12	945.6	35	51	0.048	Very Low	High	Class IV	Low	Mechanistic
9	INSULIN LISPRO	Approved	0.621	0.603	5808.0	−8.45	1567.0	52	78	0.015	Very Low	High	Class IV	Low	Mechanistic
10	EXENATIDE	Approved	0.598	0.580	4186.6	−7.23	1234.5	41	62	0.025	Very Low	High	Class IV	Low	Mechanistic

**Table 11:** Biologic therapeutic candidates identified through network analysis with development feasibility assessment.

Rank	Drug	Status	Target	Network Score	BBB Strategy	AD Evidence
1	Prasinezumab	Experimental	*α*-Synuclein	0.968	RMT engineering	Mechanistic
2	Gantenerumab	Experimental	Amyloid-*β*	0.847	Native IgG1	Clinical
3	Aducanumab	Approved	Amyloid-*β*	0.823	Native IgG1	Established

**Table 12:** Alzheimer’s disease evidence classification for top 10 drug repurposing candidates across therapeutic modalities, with mechanistic hypotheses, validation status, and critical limitations requiring experimental investigation.

Drug Name	Approved Indication	AD Trials	Mechanism Hypothesis	Evidence Level	Key Limitations	References
Memantine	Moderate-severe AD	–	NMDA antagonism; excitotoxicity reduction	Established	Symptomatic only; no disease modification	[42]
Donepezil	Mild-severe AD	–	Acetylcholinesterase inhibition; cholinergic enhancement	Established	Symptomatic only; modest cognitive benefit	[43]
Trofinetide	Rett syndrome	None	IGF-1 pathway; synaptic neuroprotection	Speculative	No AD preclinical/clinical data; mechanistic disconnect	[44]
Plerixafor	Stem cell mobilization	None	CXCR4 antagonism; neuroinflammation modulation	Mechanistic	No AD model validation; unclear BBB kinetics	[45]
Duloxetine	Depression, neuropathic pain	None	SNRI; monoaminergic modulation; potential anti-inflammatory	Mechanistic	No AD efficacy data; unclear disease-modifying potential	[46]
Sertraline	Depression, anxiety	Phase 2/3	SSRI; serotonergic modulation; BDNF upregulation	Mechanistic	Clinical trials showed no cognitive benefit	[47]
Risperidone	Schizophrenia, bipolar	Phase 4	Dopamine/serotonin antagonism; behavioral symptom control	Mechanistic	No disease modification; safety concerns (ARIA)	[48]
Quetiapine	Schizophrenia, bipolar	Phase 3	Atypical antipsychotic; behavioral symptoms	Mechanistic	No cognitive benefit; metabolic side effects	[49]
Prasinezumab	Parkinson’s (investig.)	None	Anti-*α*-synuclein; protein aggregation inhibition	Mechanistic	PD target; unclear AD relevance; BBB delivery challenge	[50]
Aducanumab	AD (controversial)	Approved	Anti-amyloid-*β*; plaque clearance	Clinical	Marginal efficacy; significant safety concerns (ARIA)	[51,52]

## Data Availability

The original contributions presented in the study are included in the article, further inquiries can be directed to the corresponding author.
